# Metabolomic profiling of shade response and in silico analysis of PAL homologs imply the potential presence of bifunctional ammonia lyases in conifers

**DOI:** 10.1111/ppl.70175

**Published:** 2025-03-27

**Authors:** Sonali Sachin Ranade, María Rosario García‐Gil

**Affiliations:** ^1^ Umeå Plant Science Centre (UPSC), Department of Forest Genetics and Plant Physiology Swedish University of Agricultural Sciences Umeå Sweden

## Abstract

Norway spruce and Scots pine show enhanced lignin synthesis under shade, along with differential expression of defense‐related genes that render disease resilience. In general, phenylalanine (Phe) is the precursor for lignin synthesis in plants, and tyrosine (Tyr) forms an additional lignin precursor specifically in grasses. Phenylalanine ammonia‐lyase (PAL) and tyrosine ammonia‐lyase (TAL) from the lignin biosynthesis pathway use either Phe or Tyr as precursors for lignin production, respectively. Grasses possess a bifunctional phenylalanine/tyrosine ammonia‐lyase (PTAL) that potentially can use both Phe and Tyr for lignin biosynthesis. Metabolomic profiles of seedlings revealed higher levels of Phe and Tyr under shade in Scots pine, while Norway spruce showed differential regulation of only Tyr under shade. Sequence analysis and phylogeny of PAL homologs in the two conifers, coupled with correlation of up‐regulation of precursors for lignin synthesis (Phe/Tyr) and enhanced lignin synthesis along with differential expression of PAL homologs under shade, suggest the potential presence of a bifunctional ammonia‐lyases (BAL) in conifers. This finding is novel and comparable to PTALs in grasses. Exome sequence analysis revealed a latitudinal variation in allele frequencies of SNPs from coding regions of putative PAL and BAL in Norway spruce, which may impact enzyme activity affecting lignin synthesis. Metabolomic analysis additionally identified metabolites involved in plant immunity, defense and stress response.

## 1 INTRODUCTION

Light is one of the essential environmental factors that plays a vital role in the regulation of plant growth and development. Shade comprises of a low red (R):far‐red (FR) ratio and is a stressful condition for plants (Hussain et al., 2019). Shade in general, is defined as a condition with a low R:FR ratio or a condition with FR‐enriched light. Twilight is similar to shade or shade‐like conditions which have a low R:FR ratio (Nilsen 1985). Under the leaf canopy, most of the R light is absorbed by the plant pigments while FR light is reflected leading to a low R:FR ratio comparable to shade (Ballare et al., 1987). Plants perceive shade as a decrease in R:FR ratio having higher FR light. Norway spruce (*Picea abies* (L.) H. Karst.) and Scots pine (*Pinus sylvestris* L.), which are economically important conifer species for the Swedish forest industry, have a contrasting response to shade or a low R:FR ratio: Norway spruce is shade‐tolerant while Scots pine is a shade‐intolerant species (Ranade et al., 2019). Norway spruce can grow, survive and thrive under shade as compared to Scots pine, which is shade‐intolerant and requires full sunlight (Grebner et al., 2021). Shade‐tolerant species have a slow relative growth rate, and a strong defense strategy compared to the shade‐intolerant species, which exhibit rapid growth and a reduced defense response (Martinez‐Garcia and Rodriguez‐Concepcion 2023). However, shade is perceived as stress and an unfavourable condition in both species. Scots pine displays shade avoidance syndrome (SAS) and Norway spruce exhibits a shade tolerance response (STR) in response to low R:FR or shade conditions (Ranade et al., 2019).

During the growth season, the northern forests in Sweden daily receive more hours of FR‐enriched light/twilight or shade‐like conditions (low R:FR) as compared to southern forests due to Sweden's geographical location. Although Norway spruce and Scots pine show contrasting responses to shade, they have adapted to latitudinal variation in twilight characterized by a northward increase in FR requirement to maintain growth (Clapham et al., 1998; Clapham et al., 2002; Ranade and García‐Gil 2013, Ranade et al., 2022a). In Norway spruce, recently, we identified a latitudinal cline in SNPs that belong to the coding regions of phytochrome, which are the central regulators of the light pathway (Ranade and García‐Gil 2023). These clinal variations in SNPs correlate with the latitudinal gradient in response to variable light quality and are proposed to represent signs of local adaptation to the light quality in Norway spruce by this study.

**FIGURE 1:**
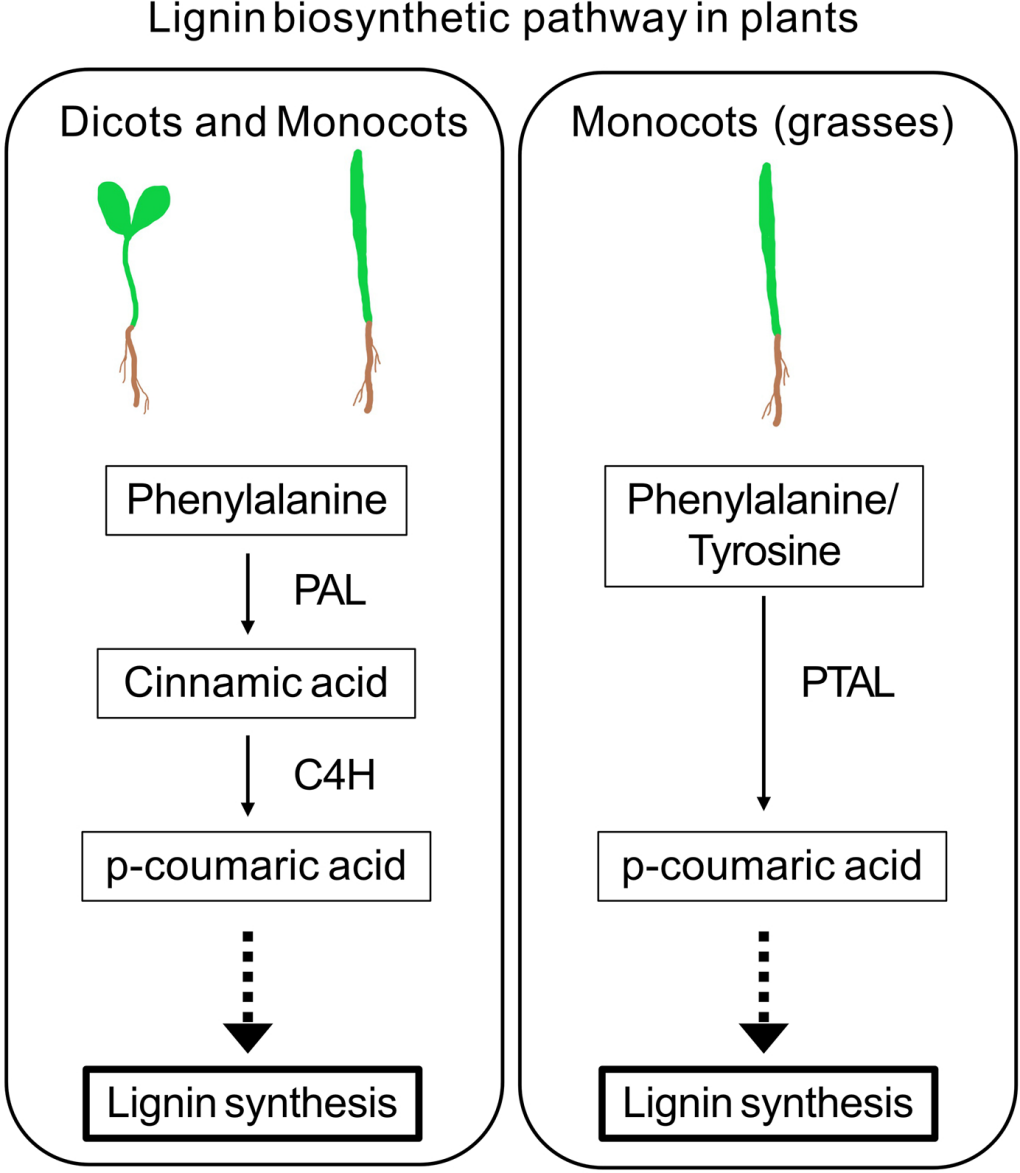
Simplified lignin biosynthetic pathway in plants showing initial steps. The enzymes that catalyse the first couple of steps in this pathway include phenylalanine ammonia‐lyase (PAL), cinnamate 4‐hydroxylase (C4H) in dicots and monocots where phenylalanine is first deaminated by the enzyme PAL to form cinnamate and then hydroxylated by cinnamate‐4‐hydroxylase (C4H) to p‐coumarate. Monocots (grasses) additionally contain the bifunctional phenylalanine/tyrosine ammonia‐lyase (PTAL) that catalyses the deamination of Phe/Tyr leading to the formation of p‐coumarate directly.

Lignin is the second most abundant polymer in the secondary cell wall that renders mechanical strength, protection against pathogens and enables transport of solutes in plants (Lee et al., 2019). Lignin is synthesized in plant cells through the phenylpropanoid metabolic pathway. Phenylalanine (Phe) and tyrosine (Tyr) are the two key amino acid precursors for lignin biosynthesis, thus defining two ways of lignification in plants (Liu et al., 2018; Figure 1). Phenylalanine ammonia‐lyase (PAL) is the first enzyme of the general phenylpropanoid pathway in most plants, where Phe is the precursor of lignin synthesis. Phe is first deaminated by the enzyme PAL and then hydroxylated by cinnamate‐4‐hydroxylase (C4H), leading to the formation of p‐coumarate that subsequently enters the later steps of the phenylpropanoid pathway, ultimately resulting in the synthesis of lignin monomers (Figure 1). The presence of a bifunctional phenylalanine/tyrosine ammonia‐lyase (PTAL) that can use both Phe and Tyr for the biosynthesis of lignin has been reported only in grasses (monocots), among plants (Barros et al., 2016). PTAL from grasses facilitates the deamination of Phe/Tyr, leading to the formation of p‐coumarate directly (Figure 1). The enzymatic active site of PAL/PTAL contains a highly conserved Ala‐Ser‐Gly – MIO (4‐methylidene‐imidazole‐5‐one) electrophilic group (Peng et al., 2022).

Light is one of the factors that regulate the synthesis of lignin. There is a decrease in lignin synthesis under shade conditions (low R:FR) in most angiosperms, which makes the plants weak and susceptible to diseases (Wu et al., 2017; Hussain et al., 2019). However, in contrast, there is enhanced lignin synthesis under shade in the two conifers species ‐ Norway spruce and Scots pine, irrespective of their contrasting shade tolerance responses (Ranade et al., 2022a, 2022b). In addition, a clinal variation in the regulation of defense gene expressions was observed in these two species; the northern populations of both conifers in Sweden showed a higher number of defense‐related genes being expressed in response to shade as compared to the southern populations. These investigations suggested that the northern populations of both conifers may be disease‐resilient as compared to the southern ones. Furthermore, the same studies proposed that these variations could be attributed as one of the underlying factors responsible for adapting to local environmental conditions, in this case, it being the adaptation to the light quality (twilight or FR‐enriched light). Adapting to local environmental conditions renders higher mean fitness to the plants. However, comprehension of the local adaptation strategies and detection of the underlying phenomenon is challenging in forest trees. In the current study, seedlings from southern and northern populations of Norway spruce and Scots pine in Sweden were grown under continuous shade conditions in growth cabinets. Metabolomic profiling of these seedlings was carried out to reveal the metabolites involved in enhanced lignin synthesis and differential defense response under shade in the southern and northern populations of both conifer species.

Moreover, we reconfirmed the enhanced lignin synthesis in both conifer species in response to shade with pyrolysis in agreement with earlier investigations (Ranade et al., 2022a, 2022b). Since all the PTAL homologs from conifers in the current work are reported based on sequence analysis, phylogeny, expression data, and results from metabolomic analyses, these are, therefore, putative genes and designated as bifunctional ammonia‐lyases (BALs) rather than the standard PTAL genes. Sequence analysis and phylogenetic analysis of the putative PAL and BAL homologs were carried out in addition to analysis of the differential expression of these genes in both conifer species in response to shade conditions. We also analyzed the latitudinal variation in the SNPs from putative PAL/BAL homologs in the different Norway spruce populations with the exome sequencing technology.

In summary, the goal of the work was to perform the metabolomic profiling of shade response in conifers, with a view to explore the lignin synthesis pathway as conifers show enhanced lignin synthesis under shade unlike the angiosperms.

## 2 MATERIALS AND METHODS

### 2.1 Seedling growth and sampling

Seeds of Norway spruce and Scots pine were collected and grown as described previously (Ranade et al., 2022a, 2022b). Briefly, seeds were collected from natural populations in Sweden from unrelated trees. Northern Norway spruce seeds were collected from Pellonhuhta (67°2′N) and the seeds for the southern population were collected between the latitudes 56°N and 58°N. Scots pine seeds were sampled from Kaunisvaara (67°5′N) and Lammhult (56°2′N), referred to as northern and southern populations, respectively. Seeds were germinated and grown in Percival growth cabinets (LED‐30 Elite series) under continuous Shade (R:FR ratio of 0.2 and a total light intensity of 36 μmol m^−2^ s^−1^) and Sun conditions (R:FR ratio equal to 1.2 and a total light intensity of 65 μmol m^−2^ s^−1^) at a temperature of 22°C on moist vermiculite (Ranade et al., 2019). These light conditions were used as they were able to trigger the shade responses in Scots pine and Norway spruce as described in our earlier work. R and FR light qualities are the two main responsible elements that plants use to determine the shade conditions and respond accordingly (Ranade et al., 2019). Seedlings were harvested at the same developmental stage when the hypocotyl is fully developed i.e., when the seed drops off and cotyledons are set free (Ranade et al., 2019). The number of days from sowing of the seeds to fully developed hypocotyls was approximately 17 ± 2 days for Norway spruce while it was 14 ± 2 days for Scots pine, under both light treatments. Harvested seedlings were collected in liquid nitrogen and stored at −80°C until further processing. Whole seedlings were used for metabolomics analysis and pyrolysis.

### 2.2 Metabolite extraction and LC–MS/GC–MS

Metabolomic analyses including liquid chromatography–mass spectrometry (LC–MS) and gas chromatography–mass spectrometry (GC–MS) were performed on whole seedlings. Eight seedlings per population, per light treatment, from Norway spruce and Scots pine were used for metabolomics. The untargeted metabolomic approach was followed and the identification of the compounds was carried out by referring to the SMC library of authentic standards (https://www.swedishmetabolomicscentre.se/). The seedlings were ground into fine powder in frozen conditions using liquid nitrogen. 10–15 mg sample per seedling was used for metabolite extraction. Detailed information regarding sample preparation, mass spectrometry and data processing is included in supplementary data (Supplementary file1).

### 2.3 Multivariate data analysis and pathway analysis of metabolomic data

Principal component analysis (PCA) was performed to create an overview of the data, investigate data integrity, identify potential outliers and explore possible trends and groupings of the samples (Jolliffe and Cadima 2016). Orthogonal projections to latent structures discriminant analysis (OPLS‐DA; Trygg and Wold 2002) were used to investigate differences in the metabolic profiles between the studied groups. A 1 + 0 component model (predictive + orthogonal) was used to avoid the risk of over‐fitting (Trygg and Wold 2002). The significance of a metabolite for classification in the OPLS‐DA model was specified by calculating the 95% confidence interval for the loadings using jackknifing (Efron and Gong 1983). All data was centered and scaled to unit variance. The OPLS‐DA model was validated with a seven‐fold cross‐validation (Wold 1978), and ANOVA of the cross‐validated models was used to define the model significance (Eriksson et al., 2008). All multivariate data analysis and model plots were performed in SIMCA 16.0 (Sartorius Stedim Data Analytics AB). Pathway analysis of the significant metabolites detected by LC–MS and GC–MS was performed using MetaboAnalyst 6.0 (Pang et al., 2024).

### 2.4 Measurement of total lignin content

Lignin, which is present in the secondary cell wall, provides mechanical strength, enables the transport of solutes in plants, and protects the plant from pathogen invasion (Lee et al., 2019). Measurement of total lignin content was performed in young seedlings (17 ± 2 days old in Norway spruce, 14 ± 2 days old in Scots pine) grown under different light treatments using Pyrolysis‐Gas Chromatography–Mass Spectrometry (Py‐GC–MS). Py‐GC–MS was performed using two approaches, one by preparing alcohol‐insoluble residue of the seedling powder (AIR1/AIR2) and the other by using crude seedling powder. Norway spruce and Scots pine seedlings grown under the Sun and Shade conditions were dried and ball‐milled into fine powder. Five seedlings per species, per population and per light treatment, were used for each pyrolysis method. Total lignin was also measured in trees ranging from 12–24 years of age from field trials by using near infrared spectroscopy (NIR).

For the AIR1/AIR2 method, firstly, ball‐milled fine powder of the seedling was washed sequentially in 80% ethanol (v:v in water) for 30 min at 95°C, 70% ethanol (v:v in water) for 30 min at 95°C, 95% MeOH (v:v in water) for 10 min at room temperature, methanol:chloroform 1:1(v:v) for 10 min at room temperature and twice with acetone. The residue was dried in vacuum‐desiccator overnight to obtain alcohol insoluble residue 1 (AIR1). Starch was removed from AIR1 by treating with *α*‐amylase from pig pancreas (100 units per 100 mg of AIR1) in 0.1 M potassium phosphate buffer, pH 7.0 containing 10 mM NaCl overnight at 37°C. The residue 2 (AIR2) was washed with 0.1 M potassium phosphate buffer, water, followed by acetone. It was dried in a vacuum desiccator overnight. 50 μg (± 10 μg) of the dried residue was applied to a pyrolyzer equipped with an autosampler (PY‐2020iD and AS‐1020E, Frontier Lab) connected to a GC/MS (7890A/5975C; Agilent Technologies AB). The pyrolysate was separated and analyzed according to Gerber et al. (2012).

To perform Py‐GC–MS with the crude seedling powder, 50 μg (± 10 μg) of ball‐milled fine powder of the seedlings was directly applied to the pyrolyzer connected to GC/MS and the pyrolysate was separated and analyzed according to Gerber et al. (2012), similar to the AIR1/AIR2 method.

For NIR, 12–24 year old Norway spruce trees from two populations maintained by Skogforsk were included – Sävar (63.89°N) and Höreda (57°N). Both populations comprised unrelated individuals, including 782 trees from Sävar (clonal archive of unrelated elite genotypes) and 1244 trees from Höreda (progeny trial of half‐sib families, one tree per family has been sampled; Morales et al., 2024). Family in this context refers to the open‐pollinated progenies per tree, i.e. seeds extracted from cones for a single tree represent one open‐pollinated progeny/family. The detailed procedure for NIR is included in the supplementary data (Supplementary file2).

### 2.5 Sequence analysis and phylogeny of putative PAL/BAL homologs in conifers

PAL and PTAL are the first enzymes of the phenylpropanoid pathway. PAL is present in most plants that use Phe as the precursor for lignin synthesis, while PTAL can use both Phe and Tyr for the biosynthesis of lignin, which is reported only in grasses (Barros et al., 2016). The PAL/PTAL protein sequences of monocots and dicots were retrieved from GenBank (https://www.ncbi.nlm.nih.gov/genbank/; Benson et al., 2013) and The Arabidopsis Information Resource (TAIR, https://www.arabidopsis.org/). The protein sequences of putative PAL/BAL for the conifer species, were retrieved from Gymno PLAZA, 1.0 (https://bioinformatics.psb.ugent.be/plaza/versions/gymno-plaza/; Proost et al., 2015) using BLAST searches performed with the characterized PAL from *Arabidopsis thaliana* and PTAL from *Brachypodium distachyon*. Multiple sequence alignment was performed using CLUSTAL O (1.2.4; Sievers and Higgins 2018). The phylogenetic tree was constructed using Phylogeny.fr with the default settings (https://www.phylogeny.fr/; Dereeper et al., 2008). In brief, alignments were created using MUSCLE (Edgar 2004), phylogeny was done using PhyML (maximum‐likelihood principle; Guindon et al., 2010) and TreeDyn (Chevenet et al., 2006) was used to construct the tree. The choice of species for this analysis was based on the literature (Xue et al., 2007; Hsieh et al., 2010; Barros et al., 2016; Jun et al., 2018; Feduraev et al., 2020). *Arabidopsis thaliana* PAL was included in the sequence alignment and phylogenetic tree as it is the most well‐studied model plant species apart from being a flowering plant; *Petroselinum crispum* PAL was included as a representative from flowering plants; *Populus trichocarpa* PAL was included as a representative from the tree species; *Brachypodium distachyon, Bambusa oldhamii*, *Oryza sativa* and *Zea mays* were included, as these grass species possess both PAL and PTAL; *Picea abies*, *Pseudotsuga menziesii*, *Picea sitchensis*, *Pinus sylvestris* and *Pinus taeda* were included in the analysis as they represent the conifer species.

### 2.6 Detection of SNP variations in putative PAL/BAL genes in Norway spruce

The exome capture dataset was recruited from the previous study described in Ranade and Garcia‐Gil, 2023 (Ranade and García‐Gil 2023). In short, 1654 individuals, unrelated parents from natural forests, originating from different latitudes across Sweden, were included in this study. The trees were divided into six populations following the same rationality described in Ranade and García‐Gil, 2023 (Ranade and García‐Gil 2023), considering the latitude‐wise variation in the amount of FR light across Sweden – the northern latitudes receive an extended period of FR light as compared with the southern ones. Trees were divided into six populations, S1‐S6 as described previously (Ranade and García‐Gil 2023): S1 comprised 245 trees from latitudes 55–57, S2–213 trees from latitude 58, S3–187 trees from latitudes 59–60, S4–213 trees from latitudes 61–62, S5–573 trees from latitudes 63–64 and S6–223 trees from latitudes 65–67. Exome capture details have been described in Baison et al., (2019). Variant calling was performed using GATK HAPLOTYPECALLER v.3.6 (Van der Auwera et al., 2013) and SNPs were annotated using SNPEFF 4 (Cingolani et al., 2012). Only bi‐allelic SNPs were included in this study. The vcf file was filtered using settings; −‐min‐alleles 2 ‐‐max‐alleles 2 ‐‐maf 0.01 ‐‐remove‐indels ‐‐minQ 10 ‐‐max‐missing 0.9. The vcf file from the current analysis is deposited in Zenodo, which is the open‐access repository developed under the European OpenAIRE program and operated by CERN (Ranade and García‐Gil 2024). SNPassoc was used to determine the allele and genotype frequencies (Gonzalez et al., 2007) and Analysis of variance and Tukey's posthoc tests (Bonferroni *p values*) were applied to determine the statistical significance of their differences. Genetic diversity among the six different populations (pairwise F_ST_ estimates) was estimated using DnaSP 6 (Rozas et al., 2017) including both synonymous + missense SNPs, for each of the putative PAL/PTAL genes. Allele frequencies in each population regarding the putative PAL/PTAL genes were calculated and then regressed on population latitude. R2 of the linear regression was computed as the proportion of the total variance of latitude explained by the frequency of each marker (Berry and Kreitman 1993), where R2 is the goodness‐of‐fit of the linear regression model.

### 2.7 Gene expression of putative PAL/BAL in response to Shade in conifers

The differential expression of putative *PAL/BAL* genes in response to Shade was derived from the transcriptome data from our earlier study in Norway spruce (Ranade et al., 2022a) and Scots pine (Ranade et al., 2022b), where the transcriptomes were analysed in seedling samples in both species from the northern and southern latitudes in Sweden, grown under Shade and Sun conditions. In brief, single‐gene differential expressions between the northern and southern latitudes in response to Shade was determined where Sun was the control, using DESeq2 (v1.12.0; Love et al., 2014). False discovery rate (FDR) adjusted p‐values were used to assess the significance of the expression of the genes while a common threshold of 5% was used throughout.

## 3 RESULTS

### 3.1 Detection of metabolites in response to Shade

LC–MS detected a total of 799 metabolites (targeted + untargeted) in Norway spruce and 781 targeted + untargeted metabolites in Scots pine (Supplementary file3, Table S1). GC–MS detected and identified 69 metabolites in Norway spruce and 68 metabolites in Scots pine. Further details on the metabolites detected with LC–MS and GC–MS in both conifer species are included in the supplementary data (Tables S1‐S5). A total of 30 identified metabolites (LC–MS + GC–MS) were significantly down‐regulated under Shade and 21 identified metabolites were significantly up‐regulated under Shade in the northern Norway spruce samples. Likewise, 41 and 25 identified metabolites (LC–MS + GC–MS) were significantly down‐regulated and significantly up‐regulated respectively, in response to Shade in the southern Norway spruce population. In the case of Scots pine, 63 identified metabolites (LC–MS + GC–MS) were significantly down‐regulated and 29 were significantly up‐regulated under Shade in the northern population, while 63 and 29 identified metabolites (LC–MS + GC–MS) were significantly down‐regulated and significantly up‐regulated respectively, in response to Shade in the southern population. Overall, a larger number of metabolites were down‐regulated under Shade in both conifers.

The PCA of LC–MS and GC–MS data for Norway spruce and Scots pine demonstrated a clear separation between all groups – North_Shade, North_Sun, South_Shade and South_Sun (Supplementary file4, Figure S1‐S3), except in case of GC–MS data for Scots pine where the PCA showed clear separation between Shade and Sun conditions, but the Northern and Southern samples were not completely separated (Supplementary file4, Figure S4). The OPLS‐DA (LC–MS and GC–MS data) showed differences between Shade and Sun conditions in both populations in both species (Supplementary file4, Figure S5‐S8). Metabolite loadings for the Northern ecotype plotted against the Southern ecotype (OPLS‐DA for South vs. North models) in Norway Spruce and Scots pine showed similarity between the models and highly similar metabolic responses to Sun and Shade conditions for both ecotypes i.e. up/down regulation of metabolites under the Sun and Shade conditions (Supplementary file4, Figure S10‐S12). The exception was in the case of Norway Spruce LC–MS data, which showed only a mild similarity in the metabolic response to light condition (Supplementary file4, Figure S9). Furthermore, Scots pine exhibited a more similar metabolic response under both light conditions compared to Norway spruce (higher Q2Y in Scots pine).

The list of selected statistically significant metabolites detected in response to Shade along with their fold change under Shade as compared to the Sun conditions is represented in Table 1, while Table S2‐S5 provided in the supplementary data gives the complete list of statistically significant metabolites that were identified by LC–MS and GC–MS in response to Shade in both conifer species, at both latitudes. Up/down‐regulation refers to up/down‐regulation of the compound under Shade conditions in these tables.

**TABLE 1:** Summary of statistically significant metabolites detected in response to Shade in Norway spruce and Scots pine – Up/Down‐regulation refers to Up/Down‐regulation of the compound under Shade conditions.

	Compound	Role	Regulation under Shade: Spruce North (Fold change under Shade in parenthesis)	Regulation under Shade: Spruce South (Fold change under Shade in parenthesis)	Regulation under Shade: Pine North (Fold change under Shade in parenthesis)	Regulation under Shade: Pine South (Fold change under Shade in parenthesis)	Reference (Regarding function)
1	L‐Leucine	Defense	Up‐regulated (2.3)	Up‐regulated (2.5)	Up‐regulated (3.6)	Up‐regulated (3.8)	(Jones and Jones 1997)
2	gamma‐Aminobutyric acid (GABA)	Defense	Up‐regulated (3.2)	Up‐regulated (1.6)	Up‐regulated (2.1)	Up‐regulated (1.2)	(Guo et al., 2023)
3	L‐Serine	Plant immunity	Up‐regulated (1.9)	Up‐regulated (2.2)	Up‐regulated (1.8)	Up‐regulated (1.1)	(Zhang et al., 2023b)
4	Betaine	Abiotic stress tolerance	Up‐regulated (2.7)	Up‐regulated (2.4)	Up‐regulated (2.8)	Up‐regulated (3.2)	(Ashraf and Foolad 2007, Giri 2011)
5	L‐Threonine	Stress response	Up‐regulated (1.6)	Up‐regulated (1.9)	Up‐regulated (1.5)	Up‐regulated (1.8)	(Charlton et al., 2008)
6	Valine	Stress response, reduces vegetative growth	Up‐regulated (5.4)	Up‐regulated (4.1)	Up‐regulated (4.6)	Up‐regulated (4.9)	(Charlton et al., 2008, Li et al., 2020)
7	Threonic acid	Defense	Up‐regulated (1.9)		Up‐regulated (1.8)		(Wen et al., 2023)
8	L‐Lysine	Defense	Up‐regulated (1.9)		Up‐regulated (1.9)	Up‐regulated (1.4)	(Wen et al., 2023)
9	Ornithine	Defense	Up‐regulated (1.9)			Up‐regulated (1.04)	(Hussein et al., 2019)
10	L‐Leucine/L‐Isoleucine	Disease resistance		Up‐regulated (3.6)	Up‐regulated (3.8)		(Jones and Jones 1997, Li et al., 2021)
11	Tryptophan	Defense			Up‐regulated (3.1)	Up‐regulated (1.1)	(Zhao et al., 2022)
12	L‐Proline	Biotic/abiotic stress tolerance	Up‐regulated (1.6)				(Ashraf and Foolad 2007)
13	Xanthone	Defense			Up‐regulated (2.2)		(Tocci et al., 2011)
14	Caffeoyl quinic acid 4	Defense			Down‐regulated (0.4)	Down‐regulated (0.2)	(Mondolot et al., 2006)
15	Coumaroyl quinic acid 2	Defense			Down‐regulated (0.6)	Down‐regulated (0.4)	(Koskimäki et al., 2009)
16	Coumaroyl quinic acid 3	Defense				Down‐regulated (0.5)	(Koskimäki et al., 2009)
17	Kaempferol	Defense		Down‐regulated (0.6)	Down‐regulated (0.2)	Down‐regulated (0.1)	(Likic et al., 2014)
18	Abietic acid	Defense	Down‐regulated (0.9)		Down‐regulated (0.5)	Down‐regulated (0.1)	(Trapp and Croteau 2001)
19	Tyrosine	Lignin synthesis, antioxidant, defense		Up‐regulated (5.7)	Up‐regulated (3.6)	Up‐regulated (3.1)	(Yadav and Chattopadhyay 2023)
20	L‐Phenylalanine	Lignin synthesis, defense			Up‐regulated (4.3)	Up‐regulated (1.7)	(Yadav and Chattopadhyay 2023)
21	Shikimic acid	Lignin synthesis	Down‐regulated (0.6)	Down‐regulated (0.7)	Down‐regulated (0.5)	Down‐regulated (0.5)	(Santos‐Sánchez et al., 2019)
22	L‐Arginine	Nitrogen reserve	Up‐regulated (2.5)	Up‐regulated (1.5)	Up‐regulated (1.6)	Up‐regulated (1.2)	(Yang and Gao 2007)
23	Asparagine	Nitrogen reserve	Up‐regulated (5.5)	Up‐regulated (4.1)	Up‐regulated (1.9)	Up‐regulated (1.6)	(Lea et al., 2007)
24	Oxoglutaric acid	TCA ‐ energy‐yielding metabolism	Down‐regulated (0.6)	Down‐regulated (0.6)	Down‐regulated (0.4)	Down‐regulated (0.5)	(Zhang and Fernie 2018)
25	Pyruvic acid	TCA ‐ energy‐yielding metabolism	Down‐regulated (0.9)	Down‐regulated (0.6)	Down‐regulated (0.3)	Down‐regulated (0.2)	(Zhang and Fernie 2018)
26	L‐Glutamine	Metabolic fuel, defense			Down‐regulated (0.5)	Down‐regulated (0.2)	(Zhang et al., 2023b)
27	Dehydroascorbic acid (DHAA)	Cellular homeostasis	Down‐regulated (0.7)	Down‐regulated (0.5)	Down‐regulated (0.2)	Down‐regulated (0.2)	(Deutsch 2000, Dreyer 2021)
28	L‐Aspartic acid	Growth and defense	Up‐regulated (1.2)	Up‐regulated (1.3)	Down‐regulated (0.8)	Down‐regulated (0.8)	(Han et al., 2021)
29	Glucose	Growth & development			Down‐regulated (0.6)	Down‐regulated (0.5)	(Yoon et al., 2021)
30	Sucrose	Growth & development	Down‐regulated (0.8)	Down‐regulated (0.7)	Down‐regulated (0.5)	Down‐regulated (0.5)	(Yoon et al., 2021)

Generally, amino acids are the building blocks of proteins and they play a vital role in the overall growth and development throughout the plant life cycle. However, studies in plant model systems have reported a few amino acids that are also associated with defense responses. Amino acids involved in plant immunity, e.g. serine (Zhang et al., 2023b), defense (leucine; Jones and Jones 1997) and stress response, e.g. threonine, valine (Charlton et al., 2008, Li et al., 2021) were found to be up‐regulated in response to Shade in both conifers at both latitudes (Table 1). The common resin component involved in plant defense e.g. abietic acid (Trapp and Croteau 2001), was found to be down‐regulated under Shade at both latitudes in Scots pine and in the northern Norway spruce population, while threonic acid, engaged in plant defense (Wen et al., 2023), was detected to be up‐regulated specifically in the northern populations of both conifers (Table 1). The amino acid proline (Ashraf and Foolad 2007) and the secondary metabolite xanthone (Tocci et al., 2011), both involved in the stress and defense response, were detected to be up‐regulated, specifically in Norway spruce and Scots pine respectively (Table 1). A higher number of phenolic compounds like e.g. Caffeoyl quinic acid 4, related to the flavonoid biosynthesis pathway (Mondolot et al., 2006), were down‐regulated in Scots pine as compared to Norway spruce populations. Tyrosine and phenylalanine involved in defense and lignin synthesis (Yadav and Chattopadhyay 2023) were up‐regulated under Shade in Scots pine in both populations, while in the case of Norway spruce, only tyrosine was observed to be up‐regulated under Shade in the southern population (Table 1). Shikimic acid involved in the biosynthesis of lignin, the aromatic amino acids phenylalanine, tyrosine and tryptophan, and most alkaloids of plants (Santos‐Sánchez et al., 2019), were found to be down‐regulated under Shade at both latitudes in both conifers (Table 1). The amino acids arginine and asparagine that are related to nitrogen reserve in plants (Yang and Gao 2007; Lea et al., 2007), were found to be up‐regulated in response to Shade in both conifer species and both populations, while oxoglutaric acid and pyruvic acid related to energy‐yielding metabolism (Zhang and Fernie 2018) were observed to be down‐regulated in both species (Table 1). Growth‐related metabolites e.g. glutamine and glucose (Yoon et al., 2021; Zhang et al., 2023b) were found to be down‐regulated in Scots pine while these were up‐regulated in Norway spruce. Figures S13‐S16 from the supplementary data (Supplementary file4) represent an overview of the metabolic pathways that were impacted under Shade in both conifer species. Alanine, aspartate and glutamate metabolism was the most impacted pathway in both species under Shade.

### 3.2 Total lignin content

Pyrolysis‐Gas Chromatography–Mass Spectrometry (Py‐GC–MS) was performed using two approaches – using AIR1/AIR2 and the crude seedling powder. Regarding the lignin pathway, monolignols are synthesized in the cytoplasm and are then translocated to the cell wall, where they undergo polymerization leading to lignin formation and deposition (Alejandro et al., 2012). AIR1 removes unpolymerized lignin (monolignols) from the cytoplasm and cell wall and thus AIR1/AIR2 gives the measurement of only the polymerized lignin present in the cell wall. Py‐GC–MS of the crude sample measures unpolymerized and polymerized lignin i.e. monolignols present in the cytoplasm and cell wall, and the polymerised lignin from the cell wall.

Although the Py‐GC–MS by AIR1/AIR2 method showed higher lignin under Shade as compared to the Sun conditions in all cases except for the southern Scots pines, it was not statistically significant. Py‐GC–MS performed with the crude wood powder showed higher lignin under Shade as compared to the Sun condition in all cases, which was statistically significant (p‐value <0.05) in the southern populations of Norway spruce and Scots pine, but it was not statistically significant in the northern populations of both conifers. Table 2 represents the means of the percentage (proportion) of total lignin derived using both methods.

**TABLE 2:** The means of the percentage (proportion) of total lignin derived using Py‐GC/MS by AIR1/AIR2 method and Py‐GC/MS with crude wood powder.

	Means of the percentage (proportion) of total lignin	
Population and light treatment	Py‐GC/MS by AIR1/AIR2	Py‐GC/MS with crude wood powder
North‐Norway spruce Shade	18.3	7.5
North‐Norway spruce Sun	18.0	6.5
South‐Norway spruce Shade	17.8	8.5
South‐Norway spruce Sun	17.6	6.4
North‐Scots pine Shade	19.1	9.0
North‐Scots pine Sun	19.0	8.3
South‐Scots pine Shade	18.2	10.0
South‐Scots pine Sun	18.4	8.2

The proportion of the total lignin content derived by NIR from Sävar (north of Sweden) was detected to be higher than the proportion of total lignin in trees from Höreda (south of Sweden; p‐value <0.05). The mean percentage (proportion) of total lignin content in trees from Höreda was 25.8, while it was 26.1 in the case of trees from Sävar.

### 3.3 Sequence analysis and phylogeny of putative PAL/BAL homologs in conifers

**FIGURE 2:**
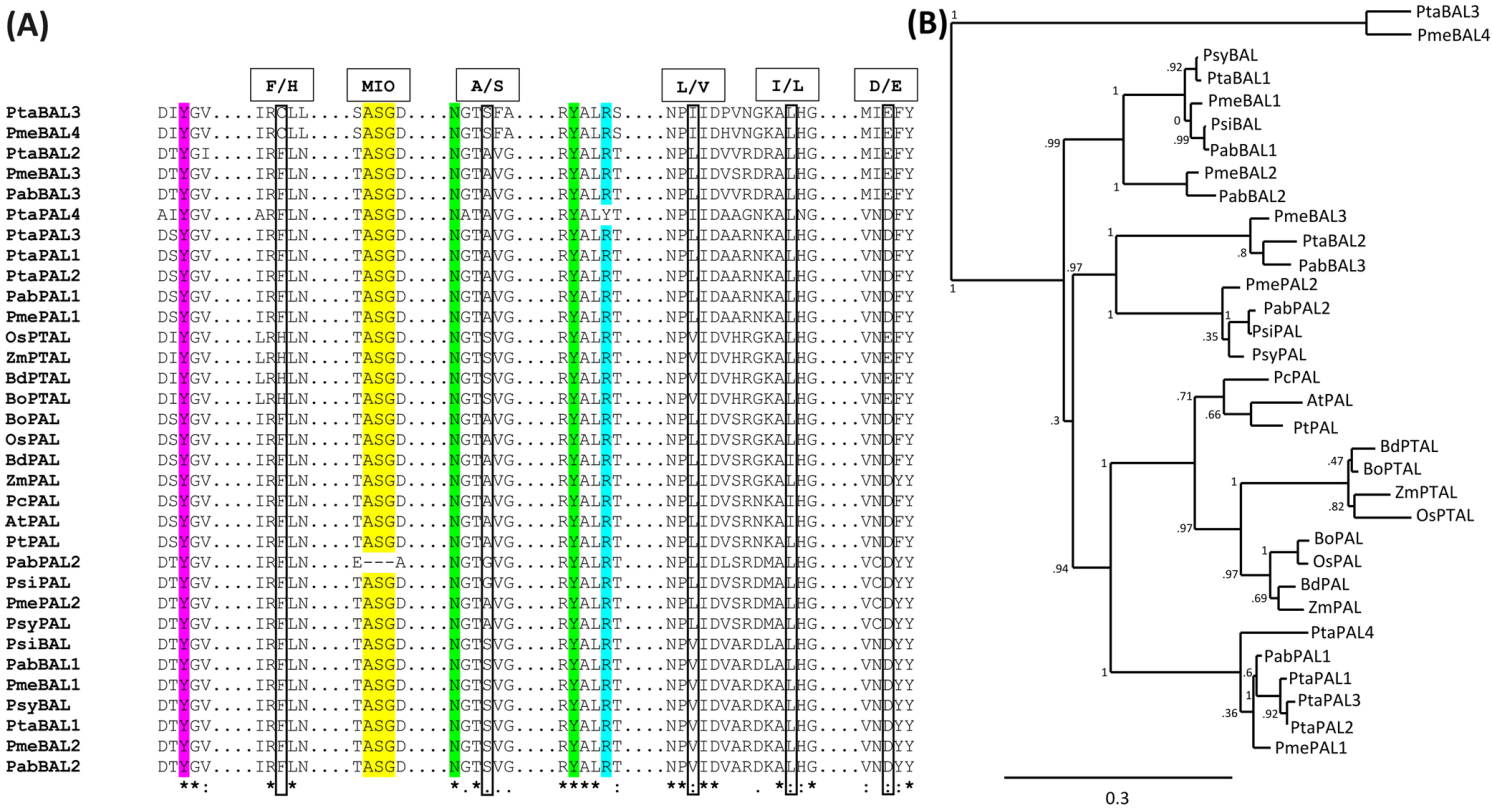
**Sequence alignment and phylogenetic tree of PALs and PTALs** ‐ At: *Arabidopsis thaliana*; Bd: *Brachypodium distachyon*; Bo: *Bambusa oldhamii*; Pc: *Petroselinum crispum*; Pt: *Populus trichocarpa*; Os: *Oryza sativa*; Zm: *Zea mays*; Pab: *Picea abies*; Pme: *Pseudotsuga menziesii*; Psi: *Picea sitchensis*; Psy: *Pinus sylvestris*; Pta: *Pinus taeda*. (A) Partial alignment of PALs and PTALs from plant model systems and putative PALs and BALs from conifer species showing conserved amino acid residues. Colour codes – magenta: catalytically essential tyrosine; yellow: MIO region; green: asparagine and tyrosine stabilizing the MIO group; blue: arginine responsible for binding the carboxylic group of the substrate. Residues involved in substrate specificity for phenylalanine/tyrosine are marked with boxes (F/H, A/S, L/V, I/L and D/E). (B) Phylogenetic tree of PALs and PTALs from plant model systems and putative PALs and BALs from conifer species.

All the PTAL homologs from conifers in the current work are designated as BALs rather than the standard PTAL genes, as they are yet to be functionally characterized. The sequence details of all the PAL/PTAL sequences from monocots, dicots and putative PAL/BAL sequences from conifer species included in the analysis are represented in the supplementary data (Supplementary file3, Table S6‐S7). The partial alignment of PALs and PTALs from the model plants and putative PALs and BALs from conifer species (Figure 2A) suggests that the functional domains/residues are well conserved across angiosperms and conifers. The catalytically essential MIO region formed from an alanine‐serine‐glycine triad, which is conserved in model plants, was found to be present in all the conifer species except PabPAL2. Several other key amino acid residues required for the functioning of the lyase were also found to be conserved in conifers such as tyrosine as the catalytic base, arginine interacting with the carboxylic group of the substrate, and, tyrosine and asparagine involved in stabilization of the electrophilic MIO within the catalytic site (Varga et al., 2021). Residues involved in substrate specificity for Phe/Tyr in PALs/TALs/PTALs according to the previous studies were detected in the putative PALs and BALs from conifer species (F/H, A/S, L/V, I/L, D/E; Xue et al., 2007; Hsieh et al., 2010; Barros et al., 2016; Jun et al., 2018; Feduraev et al., 2020). The F/H residue is crucial for substrate specificity; F is conserved between all the PALs in angiosperms while the H residue is conserved in the PTALs (Barros et al., 2016). Although none of the conifers possessed the H residue at the position, the other residues involved in substrate specificity for Phe/Tyr seem to be conserved in conifers with exceptions (Figure 2A). Similar to the bifunctional PTALs from grasses, PtaBAL2, PabBAL3 and PmeBAL3 have a conserved E residue rather than a D residue present in PALs (refer to D/E position in Figure 2A). Likewise, PtaBAL3 and PmeBAL4 have S and E (refer A/S and D/E position in Figure 2A), while PsiBAL, PmeBAL1, PmeBAL2, PsyBAL, PtaBAL1, PabBAL1, PabBAL2 have S and V (refer A/S and L/V position in Figure 2A), similar to PTALs from grasses. The alignment of the complete sequences of PAL/BALs (Supplementary file 4, Figure S17) showed high sequence similarity among the angiosperms and conifers, especially in the regions containing the functional domains/residues, including the conserved motif “GTITASGDLVPLSYIA” with the MIO region (ASD; He et al., 2020). The putative PALs and BALs from conifer species do not tend to blend and instead appear to be well separated into distinct clades. Moreover, they also form distinct clades separated from the dicots and monocots (Figure 2B).

### 3.4 Clinal variation in SNPs detected in putative PAL/BAL in Norway spruce

Out of the two PALs and three BALs from Norway spruce, SNPs were detected in the coding regions of one PAL (PabPAL2; 14 missense and 20 synonymous) and two BALs (PabBAL1; 3 missense and five synonymous and PabBAL3: four missense and two synonymous; Supplementary file3, Table S8). Statistically significant clinal variations in the allele and genotype frequencies were detected in two synonymous and one missense mutation in PabPAL2, while PabBAL1 and PabBAL3 showed significant latitudinal variation in two missense mutations, respectively (Table 3). One‐way ANOVA of the allele frequencies and genotype frequencies of SNPs detected in PAL/BAL showing cline is represented in the supplementary data (Supplementary file3, Table S9‐S10).

**TABLE 3:** SNPs in the putative PAL/BAL genes in Norway spruce showing clinal variation and the population‐wise allele frequencies of the reference and alternate alleles.

Gene	Mutation	Variation	Allele	Population‐wise allele frequency					
				S1	S2	S3	S4	S5	S6
PabPAL2	Synonymous	Ala228Ala: Reference T, alternate C; GCT → GCC	Reference (T)	0.50	0.50	0.50	0.49	0.48	0.47
		(Nonpolar, hydrophobic → Nonpolar, hydrophobic)	Alternate (C)	0.50	0.50	0.50	0.51	0.52	0.53
	Synonymous	Pro356Pro: Reference G, alternate C; CCG → CCC	Reference (G)	0.97	0.96	0.96	0.95	0.92	0.86
		(Nonpolar, hydrophobic → Nonpolar, hydrophobic)	Alternate (C)	0.03	0.04	0.04	0.05	0.08	0.14
	Missense	Ala383Thr: Reference G, alternate A; GCA → ACA	Reference (G)	0.90	0.90	0.87	0.86	0.85	0.77
		(Nonpolar, hydrophobic → Polar, uncharged)	Alternate (A)	0.10	0.10	0.13	0.14	0.15	0.23
PabBAL1	Missense	Ala535Val: Reference C, alternate T; GCC → GTC	Reference (C)	0.90	0.90	0.87	0.88	0.84	0.80
		(Nonpolar, hydrophobic → Nonpolar, hydrophobic)	Alternate (T)	0.10	0.10	0.13	0.12	0.16	0.20
	Missense	Ala537Val: Reference C, alternate T; GCT → GCT	Reference (C)	0.97	0.97	0.95	0.93	0.89	0.86
		(Nonpolar, hydrophobic → Nonpolar, hydrophobic)	Alternate (T)	0.03	0.03	0.05	0.07	0.11	0.14
PabBAL3	Missense	Ser595Phe: Reference C, alternate T; TCC → TTC	Reference (C)	0.94	0.95	0.91	0.87	0.89	0.83
		(Nonpolar, uncharged → Nonpolar, hydrophobic)	Alternate (T)	0.06	0.05	0.09	0.13	0.11	0.17
	Missense	Leu601Phe: Reference A, alternate T; TTA → TTT	Reference (A)	0.95	0.95	0.92	0.89	0.89	0.85
		(Nonpolar, hydrophobic → Nonpolar, hydrophobic)	Alternate (T)	0.05	0.05	0.08	0.11	0.11	0.15

**FIGURE 3:**
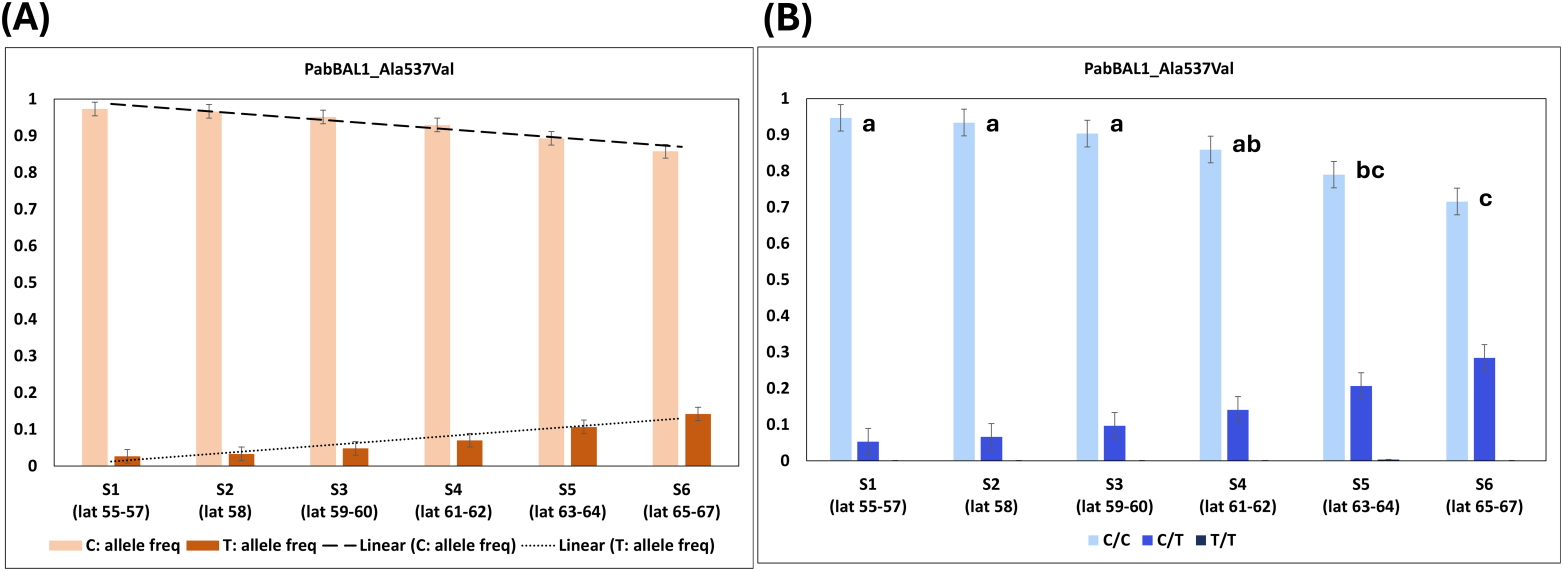
Latitudinal variation in the SNP (Ala537Val) from the putative PabBAL1 gene in Norway spruce populations across Sweden, which was the steepest among all the SNPs detected in the putative PAL/BAL genes. (A) Cline in the allele frequencies of Ala537Val. (B) Cline in the genotype frequencies of Ala537Val. One‐way ANOVA and Tukey's posthoc test was performed with the genotype frequencies. Tukey's post hoc categorization is indicated above the bars.

Ala537Val from PabBAL1 displayed the steepest cline among all (Figure 3), meaning the difference between the allele frequencies between the extreme southern and northern populations was the highest. Other SNPs from PabPAL2, PabBAL1 and PabBAL3 that showed latitudinal variation in the allele and genotype frequencies are represented in Figures S18‐S23 included in the supplementary data. The variations that did not show any latitudinal variation in their allele/genotype frequencies could be referred to as controls (Supplementary file3, Table S8). These include several SNPs from PabPAL2, PabBAL1 and PabBAL3. No SNPs were detected in PabPAL1 and PabBAL2.

**FIGURE 4:**
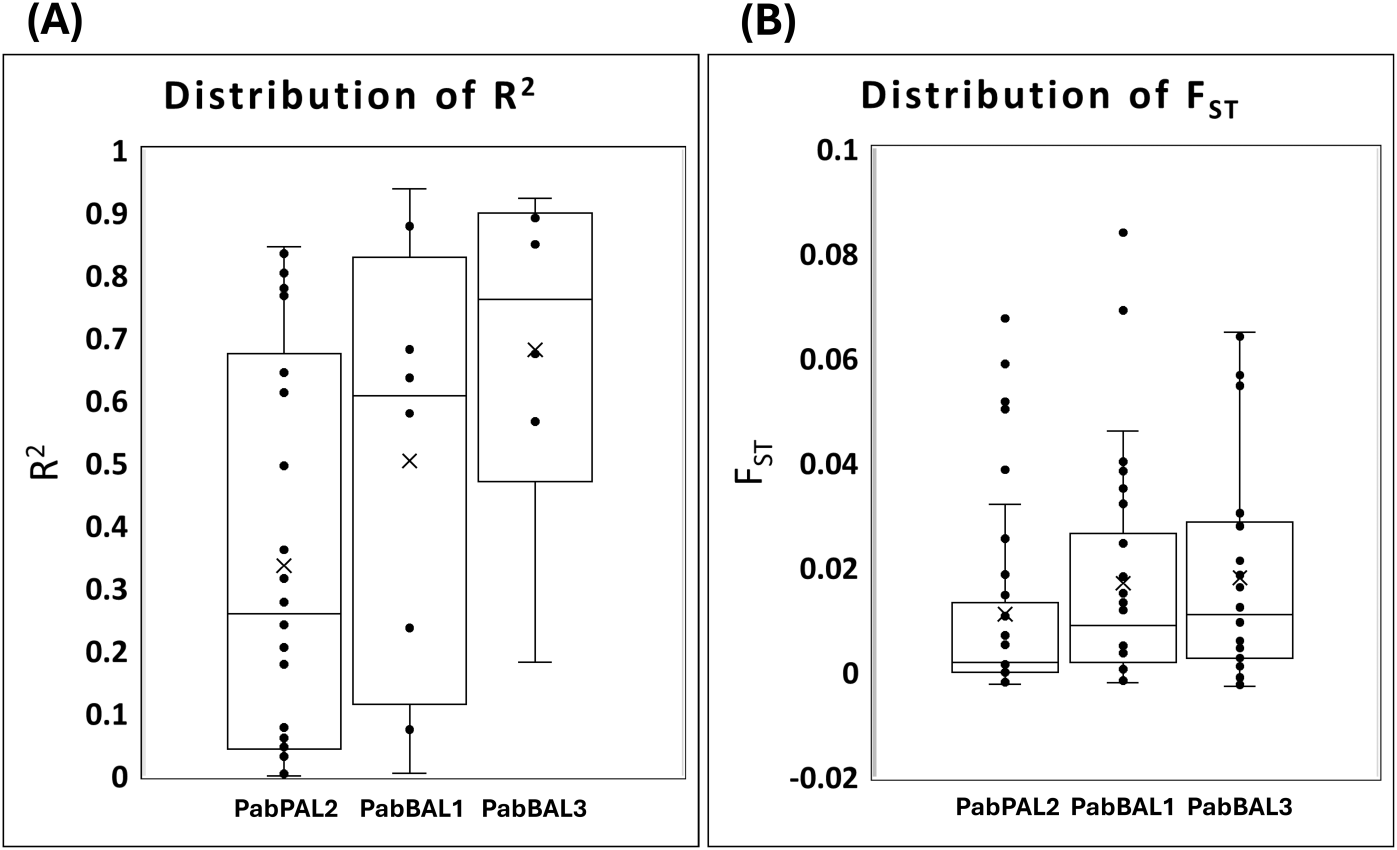
R2 and F_ST_ were calculated with the allele frequencies of all the missense and synonymous SNPs detected in the putative PAL/BAL genes of the Norway spruce populations in Sweden. (A) The distribution of R2. (B) The distribution of F_ST_. Black circles represent the R2/F_ST_ values for respective PAL/BAL genes and “x” represents the mean value of R2/F_ST_ for respective genes.

PabBAL3 showed the highest F_ST_ values along with higher and less dispersed R^2^ values as compared to PabPAL2 and PabBAL1 which suggests that it may exhibit more precise clinal variation among the three genes (Figure 4), considering all the SNPs taken together. However overall, in accordance with the literature available on Norway spruce summarised previously (Ranade and García‐Gil 2021, 2023), the pair‐wise F_ST_ estimates for six populations of Norway spruce across Sweden (Table 4) were low, indicating low population genetic differentiation.

**TABLE 4:** Pairwise F_ST_ estimates for six populations of Norway spruce across Sweden involved in the detection of clinal variation in the SNPs in the putative PAL/BAL genes.

Population	S2	S3	S4	S5	S6
S1	0	0.00198	0.00349	0.00143	0.01225
S2		0	0	0	0.00604
S3			0	0	0.00797
S4				0	0.01026
S5					0.00375

### 3.5 Variation in expression of putative PAL/BAL in response to Shade

The results from the expression data of putative *PAL/BAL* genes generated in our earlier work (Ranade et al., 2022a, 2022b) under Sun and Shade conditions in Scots pine and Norway spruce from both latitudes are summarised in Table S6‐S7 (Supplementary file3). Significant differential expression was not detected in most putative PAL/BAL genes in both conifers. However, *PabPAL2* from Norway spruce was up‐regulated under Shade, which coincided with higher lignin synthesis in the northern population but not in the southern population, which is relevant from the point of view of the current study.

## 4 DISCUSSION

### 4.1 Shade tolerance and latitudinal variation in response to shade in conifers

As mentioned in the introduction, shade is a condition with low R:FR ratio or with FR‐enriched light. Twilight is similar to shade as it also has a low R:FR ratio (Nilsen 1985). Moreover, shade is perceived by the plants as a decrease in R:FR ratio, thus having higher amount of FR light. Scots pine is shade‐intolerant, and Norway spruce is a shade‐tolerant species (Ranade et al., 2019). Generally, the underlying molecular response and mechanisms are contrasting in shade‐tolerant and shade‐intolerant species, which is evident from the literature (Ruberti et al., 2012; Martinez‐Garcia and Rodriguez‐Concepcion, 2023). Conifers, including Scots pine and Norway spruce, display similar responses under shade as flowering plants, which is demonstrated by our earlier work (Ranade et al., 2019). Yet, both species have adapted to the local light conditions and show latitudinal variations in growth depending on the quality of light (e.g. FR‐enriched) and duration of light exposure which varies latitude‐wise. Both species exhibit interesting responses to shade, twilight or FR‐enriched light. Scots pine is shade intolerant, meaning it does not thrive under shade or FR‐enriched light, yet there is a northward increase in FR requirement to maintain growth. Norway spruce also shows a higher requirement of FR light in the northern latitudes, but being a shade‐tolerant species, it can thrive well under shade conditions anyway. Norway spruce shows a clinal variation in hypocotyl elongation under shade conditions. Latitudinal variations in SNPs in key light genes such as phytochrome may contribute to the understanding of shade response in Norway spruce. Similar reasoning can be attributed to Scots pine as well. The most interesting responses with reference to the regulation of defense‐related genes were noted in both conifers in the context of the latitudinal variation. In agreement with the shade tolerant nature of Norway spruce, an equal number of defense‐related genes were up‐regulated and down‐regulated, respectively, under shade conditions within latitudes comparisons (comparisons separately performed in the southern populations and in the northern populations respectively). However, the north versus south comparisons showed a significantly higher number of defense‐related genes up‐regulated in the northern population in response to shade or FR‐enriched light. Likewise, in Scots pine, the within‐latitude comparisons revealed a statistically significant higher number of defense‐related genes that were down‐regulated under shade which holds true owing to its shade intolerant character. Nevertheless, the north versus south comparisons showed a significantly higher number of defense‐related genes being up‐regulated in the northern population as compared to the southern population, under shade conditions. This enhanced defense‐related gene expression under shade in the northern latitudes in both conifers were interpreted as local adaptation to the extended FR‐enriched light (or shade or twilight) prevalent in the northern latitudes (Ranade et al., 2019).

In the current work, higher lignin was detected under Shade (low R:FR or FR enriched light) as compared to the Sun conditions in both conifer species. The NIR data from Norway spruce trees showed that the proportion of total lignin content is higher in the northern trees as compared to the southern ones. In addition, clinal variation in SNPs was detected in the putative PAL/BALs in Norway spruce, which are the first enzymes of the lignin biosynthetic pathway. Variations in SNPs in the putative PAL/BALs may alter enzyme activity that may lead to differential lignin synthesis. Higher lignin content in the northern trees and SNP variations in PAL/BALs are concurrent with the latitudinal variation in the natural light conditions (higher amount of FR light in the north) and the northward increase in FR requirement to maintain growth in Norway spruce.

### 4.2 The presence of a potential BAL gene family in conifers that catalyses Tyr assimilation into the lignin biosynthetic pathway

Lignin is synthesized from Phe/Tyr via the phenylpropanoid metabolic pathway in plant cells (Liu et al., 2018). In most dicot plants, lignin is synthesised from Phe, where the first step involves the deamination of Phe by PAL producing cinnamic acid. Cinnamate 4‐hydroxylase (C4H) produces p‐coumaric acid from cinnamic acid by introducing a hydroxyl group into the phenyl ring of cinnamic acid and p‐coumaric acid forms the precursor for all monolignols. Monolignol biosynthesis in grasses is reported with fewer steps; p‐coumaric acid is produced directly from Tyr through the PTAL enzyme (Barros et al., 2016). Thus, in dicots, lignin is synthesized only from Phe, while both Phe and Tyr form the basis for lignin synthesis in monocots. The biosynthesis of lignin in plants requires the provision of its essential precursors, Phe or Tyr. The supply of exogenous Phe or Tyr leads to a lignin deposition which indicates that the availability of Phe or Tyr is a deciding factor for lignin synthesis (Wang et al., 2018; Feduraev et al., 2020).

In the current work, the metabolomic analysis detected up‐regulation of Phe and Tyr together with higher lignin synthesis in Scots pine, whereas in Norway spruce, up‐regulation of only Tyr was detected along with higher lignin synthesis. In grasses, although Tyr is preferentially incorporated into the S‐units of lignin, Phe is also utilised in the formation of the S‐units (Barros et al., 2016). In this regard, it is worth noting that conifers mainly contain G‐units with little or no S‐units of lignin (Ralph et al., 2019). Therefore, conifers may follow alternative mechanisms to efficiently utilize Tyr for lignin formation, which needs further validation. Lastly, although there is evidence of increased lignin synthesis under Shade, the concurrent down‐regulation of shikimic acid, which is one of the key elements involved in the biosynthesis of lignin, also warrants further investigation in conifers.

Multiple PAL/PTAL genes are found in monocots, e.g. in grasses that represent distinct PAL/PTAL monocot‐specific clades in phylogenetic trees (Barros et al., 2016; Schaker et al., 2017). Likewise, many PAL genes have been described in dicots (e.g. *Arabidopsis thaliana* and tomato) which form a dicot‐specific PAL clade in the phylogenetic tree (Schaker et al., 2017; Zhang et al., 2023a). Previous research has documented a phylogenetically diverse set of PAL enzymes in gymnosperms (Bagal et al., 2012) and suggested the existence of a gymnosperm‐specific lineage of PAL genes (Bagal et al., 2012; Neale et al., 2017). Our findings on the PAL homologs in Scots pine and Norway spruce align with these studies. The phylogenetic tree with the putative PAL/BAL sequences detected in the conifers showed distinct clades comprising either PAL or BAL, also the respective clades are distinct from the dicots and monocots. Additionally, our research contributes to a novel discovery of a potential BAL gene family in conifers, which was previously thought to be absent (Barros et al., 2016).

Finally, the novel detection of potential BALs in both conifer species with supporting evidence from expression data, sequence analysis and phylogeny coupled with the up‐regulation of Tyr and enhanced lignin synthesis under Shade, strongly supports the concept of Tyr as a probable precursor of lignin biosynthesis in conifer species which is a new proposition put forth by this investigation. However, further research is required to validate the functionality of BALs and lignin synthesis using Tyr in conifers.

### 4.3 The possible role of PAL and BAL gene families in the local adaptation through lignin content regulation

Our previous investigation regarding the transcriptomic data in Scots pine and Norway spruce in response to Shade (Ranade et al., 2022a, 2022b), indicates that the PAL/BAL gene family is expressed in conifers (Supplementary file3, Table S6‐S7). In Norway spruce, *PabPAL2* was found to be up‐regulated under Shade together with enhanced lignin synthesis in the northern but not in the southern population. Furthermore, our current results revealed a latitudinal cline in the missense variations from the coding regions of PAL and BALs in Norway spruce populations across Sweden. Ala537Val from PabBAL1 may lead to minor or no alterations in the chemical properties of amino acids, as both Ala and Val are nonpolar/hydrophobic. However, Ala383Thr from PabPAL2 and Ser595Phe from PabBAL3 represent the change in the properties of amino acids, as the former represents a conversion from nonpolar/hydrophobic to polar/uncharged while the latter is the conversion from nonpolar/uncharged to nonpolar/hydrophobic. Variations in SNPs in the coding regions of the putative PAL/BAL genes in Norway spruce, especially when they result in amino acid substitutions with different chemical properties, may alter protein function. This can affect enzyme activity and may lead to differential lignin synthesis, independent of gene expression regulation. Such clinal variation suggests the potential role of the PAL/BAL gene family in local adaptation through lignin synthesis regulation, meriting further investigation. It is worth noting that although an allele frequency between 0.10–0.15 may appear low, it still, for example accounts for approximately 30% of the northernmost population S6 (lat 65–67; Figure 3). This means that nearly 30% of this population is heterozygous (C/T, a missense mutation) for PabBAL1_Ala537Val, where this frequency shows a steady latitudinal decrease in frequency southwards. More importantly, the trend or cline in allele frequency for the missense mutation is crucial, as it indicates local adaptation to the prevailing light conditions in these latitudes. This highlights a potential differential regulation of lignin synthesis along the cline, which could be linked to an adaptive defense mechanism that appears more pronounced in the northern populations. This observation has been previously documented in earlier research work by Hansson et al., (1998), which demonstrated that the northern Norway spruce populations showed the strongest resistance to fungus (*Gremmeniella abietina*) attacks.

Another aspect that supplements the higher lignin synthesis in northern Norway spruce populations in response to Shade is the differential expression of the MYB3 transcription factor which is involved in the repression of lignin synthesis. Lower expression of two copies of the MYB3 transcription factors was detected in the northern Norway spruce populations as compared to the southern ones in response to Shade (Ranade et al., 2022a). Interestingly, one of these MYB3 copies also showed a steep latitudinal cline in allelic and genotypic frequencies of a missense SNP in its coding region (Ranade and García‐Gil 2021).

Enhanced lignin synthesis under Shade reported in the current study aligns with our previous studies in Norway spruce and Scots pine (Ranade et al., 2022a, 2022b). Although the higher lignin detected under Shade in most cases was not statistically significant (e.g. AIR1/AIR2), it is important to highlight that there is a trend for enhanced lignin synthesis under Shade conditions in both conifer species in both northern and southern ecotypes. Yet, above all, this contrasts with most angiosperms, where there is a significant decrease in lignin synthesis in response to Shade (Wu et al., 2017; Hussain et al., 2019). The probable reason for the lignin content detected by AIR1/AIR2 not being statistically significant as compared to the Py‐GC–MS with the crude sample may be because the monomers are yet being synthesized and are being transported to the cell wall but have not yet undergone polymerization to form lignin. Therefore, the measurement of lignin monomers + polymerized lignin (Py‐GC–MS with the crude sample) was statistically significant as compared to the lignin content measured with Py‐GC–MS performed by AIR1/AIR2 method, which measures only the polymerized lignin. Lignin formation in the cell wall is an irreversible process and it is this irreversibility that governs the necessity for the strict regulation of the lignification process (Wang et al., 2013). Therefore, another reason may be attributed to seedlings representing a very early stage of development in the process of lignification in tree species (Ruzicka et al., 2015), probably not a very likely stage to see the striking differences in lignin content. Regarding the north versus south comparisons in both conifers, no significant difference in lignin content was detected and this could also be attributed to the early developmental stage of the seedlings. Yet, the older Norway spruce trees from the north (Sävar) showed a significantly higher lignin content (NIR) as compared to the trees from the southern population (Höreda). Likewise, in the case of Scots pine, the fold change in the increase of Phe and Tyr under Shade is higher in the northern as compared to the southern populations (Table 1). Our previous studies in Norway spruce and Scots pine have shown that latitudinal variations in gene expression related to tree defense appear to be influenced by differences in FR‐enriched light or shade‐like conditions across latitudes. In addition, there is enhanced lignin synthesis in response to Shade or FR‐enriched light in both species. These studies concluded that enhanced lignin synthesis coupled with higher defense‐related gene expression in northern populations may render disease resilience to the northern trees compared to the southern ones. This finding is further supported by the current analysis. The detection of potential BALs and latitudinal variation in the SNPs may lead to differential lignin synthesis in the two populations which is well supported by the detection of Phe/Tyr levels. Lignin acts as a barrier to pathogens, thus offering protection to the plants against diseases (Lee et al., 2019). These findings support the hypothesis that higher lignin content in northern conifer species is due to the increased exposure to FR‐rich light during the growth season, suggesting a combination of plastic and adaptive genetic responses (Ranade and García‐Gil 2021; Ranade et al., 2022a, 2022b).

### 4.4 Detection of metabolites related to plant defense under Shade

In addition to lignin biosynthesis, Phe and Tyr also serve as precursors for several metabolites having diverse physiological functions as antioxidants and defense compounds (Pascual et al., 2016; Schenck and Maeda 2018). Threonic acid, which is specifically involved in defense (Wen et al., 2023), was found to be up‐regulated under Shade in the northern populations of both conifers and amino acids generally involved in defense, e.g. L‐leucine (Jones and Jones 1997) was found to be up‐regulated in response to Shade in both conifers at both latitudes. Recently, it was demonstrated that threonic acid along with lysine was critical for recruiting beneficial microorganisms that protected the plants from pathogen attacks in cucumbers (Wen et al., 2023). The key role played by the leucine‐rich repeat proteins in plant defense has been well documented (Jones and Jones 1997). Threonic acid is a weak sugar acid derived from threose while L‐threonic acid is produced by the degradation of ascorbic acid under oxidative conditions at alkaline pH (Loewus 1999). Ascorbic acid helps plants to defend against oxidative stress as it is the most abundant water‐soluble antioxidant (Shen et al., 2021). Ascorbic acid is reversibly oxidized into dehydroascorbic acid (DHAA) upon exposure to light, heat, transition metal ions and low alkaline pH (Thurnham 2000; Yin et al., 2022). The stability of DHAA lasts only for a few minutes and it is further irreversibly hydrolyzed to form 2,3‐diketogulonic acid (Zilva 1928). The reduction of DHAA leads to the formation of ascorbic acid which takes part in regulating cellular homeostasis (Deutsch 2000; Dreyer 2021). Cellular homeostasis is crucial for the establishment of balanced conditions for the controlled commencement and performance of various biochemical processes. DHAA was detected to be down‐regulated in both species at both latitudes in response to Shade. However, gamma‐aminobutyric acid (GABA), which not only helps the plant to respond to biotic and abiotic stresses but is also involved in maintaining cellular homeostasis (Guo et al., 2023), was up‐regulated under Shade in both species at both latitudes.

Flavonoids play a key role in plant defense by protecting plants from different biotic and abiotic stresses (Panche et al., 2016; Divekar et al., 2022). Phenolic compounds e.g. caffeoyl quinic acid 4, coumaroyl quinic acid 2 and coumaroyl quinic acid 3 (Table 1, Tables S2‐S5 from Supplementary file3) related to flavonoid biosynthesis were down‐regulated in Scots pine, while in Norway spruce these compounds were not statistically significant. This could be extrapolated as the SAS response in Scots pine which is associated with reduced defense responses as compared to the STR response in Norway spruce, where no significant difference in the number of defense‐related genes was reported under the Sun and Shade conditions (Ranade et al., 2019). In this context, the regulation of defense‐related metabolites is more pronounced in Norway spruce in general, meaning that overall, the fold change of the up‐regulated defense‐related metabolites is higher and the fold change of down‐regulated defense‐related metabolites is lower in Norway spruce as compared to Scots pine.

### 4.5 Detection of metabolites related to plant growth and development under Shade

The metabolic pathways commonly involved in the growth and development of plants, such as glycolysis/gluconeogenesis, starch and sucrose metabolism, the pentose phosphate pathway, carbon fixation and TCA cycle were altered in response to Shade in both conifer species (Figures S13‐S16 from the Supplementary file4). As Norway spruce is shade‐tolerant, it can grow, survive and thrive under shade as compared to Scots pine which is shade‐intolerant. The growth‐related metabolite aspartic acid was found to be up‐regulated under Shade in Norway spruce, while it was down‐regulated in Scots pine. Similarly, Shade did not significantly affect glucose regulation in Norway spruce, but glucose was found to be down‐regulated in Scots pine. Glucose is synthesised during photosynthesis using carbon dioxide and water using light; glucose is the key carbon source acting as a signalling molecule, regulating various metabolomic processes (Siddiqui et al., 2020). Glucose affects plant growth, improves the harmful effects of abiotic stress by increasing the level of antioxidants and induces the synthesis of chlorophyll, thereby regulating photosynthesis (Siddiqui et al., 2020). While sucrose is down‐regulated under Shade in both conifers, the fold change is lower in Scots pine. Sucrose is synthesized from the monosaccharides fructose and glucose in photosynthetically active cells. As sucrose is a disaccharide, its usage is more energy efficient for transport and storage as compared to fructose and glucose (Geiger 2020). In addition, as sucrose is a non‐reducing sugar, therefore it cannot be oxidized, and intermediate reactions with other molecules do not take place (Geiger 2020).

Oxoglutaric acid and pyruvic acid, which are considered as energy‐yielding metabolites (Zhang and Fernie 2018) were detected to be down‐regulated under Shade in both conifers, following the similar trend observed for sugars. However, the fold change of their down‐regulation was lower in Scots pine as compared to Norway spruce, which may be attributed to its shade‐tolerant nature. Glutamine, which is known to function as a metabolic fuel, apart from taking part in defense (Zhang et al., 2023b), was found to be down‐regulated in Scots pine, whereas Shade did not alter its regulation in Norway spruce. This again can be due to the shade‐tolerant characteristic feature exhibited by Norway spruce.

## 5 CONCLUSION

The current analysis reports new findings regarding the lignin pathway in conifers. Based on the sequence analysis and phylogeny of potential PAL/BAL homologs in conifers coupled with the correlation of up‐regulation of the precursors of lignin (Phe/Tyr), we propose the potential presence of BALs and biosynthesis of lignin using Tyr in conifers. Yet, additional research is needed to validate the functionality of BALs and to reveal the underlying molecular mechanism involved in lignin biosynthesis using Tyr as a precursor in conifers.

## AUTHOR CONTRIBUTIONS

S.S.R. contributed to the experimental design, experiment performance, data collection, data analysis and interpretation, and manuscript writing. M.R.G.G. contributed with experimental design, data analysis and interpretation, and manuscript writing. Both authors read and approved the manuscript.

## CONFLICT OF INTEREST STATEMENT

The authors declare no conflict of interest.

## Supporting information


**Supplementary file1**.

**Supplementary file2**.

**Supplementary file3**.

**Supplementary file4**.

## Data Availability

The vcf file from the current analysis containing data from the exome sequencing results is deposited in Zenodo (https://doi.org/10.5281/zenodo.12605324). All other data are included in the supplementary data.
