# Supplementary material for: Metabolomic profiling of shade response and in silico analysis of PAL homologs imply the potential presence of bifunctional ammonia lyases in conifers
Source: Physiol Plant. 2025 Mar 27;177(2):e70175. doi: 10.1111/ppl.70175 (PMC11949857; doi:10.1111/ppl.70175)
Supplement: Supplementary file 3 — Supplementary file3. [file PPL-177-e70175-s003.pdf]

Table S1 Number of metabolites detected in Norway spruce and Scots pine in response to shade				
Species	Processing type	Metabolites detected	Significantly down-regulated metabolites	Significantly up-regulated metabolites
Spruce North	LCMS	799	141 (identified + non identified)	45 (identified + non identified)
Spruce South	LCMS	799	137 (identified + non identified)	58 (identified + non identified)
Pine North	LCMS	781	264 (identified + non identified)	73 (identified + non identified)
Pine South	LCMS	781	218 (identified + non identified)	69 (identified + non identified)
Spruce North	GCMS	69	15 (identified)	3 (identified)
Spruce South	GCMS	69	13 (identified)	9 (identified)
Pine North	GCMS	68	18 (identified)	15 (identified)
Pine South	GCMS	68	16 (identified)	13 (identified)

Table S2 Metabolites detected in Norway Spruce_north: Sun vs Shade				
**Compound Name**	**HMDB**	**KEGG**	**Direction in Shade**	**Pathway**
(±)-Taxifolin	HMDB0303943	C01617	Down	
1-Aminocyclopropanecarboxylic acid	HMDB0036458	C01234	Up	
4-Guanidinobutanoic acid	HMDB0003464	C01035	Down	Arginine and proline metabolism
Abietic acid		C06087	Down	
Alanine	HMDB0000161	C00041	Up	Alanine, Aspartate, Glutamate metabolism<br>Carbon fixation in photosynthetic organisms
alpha-Tocopherol	HMDB0001893	C02477	Down	
Benzyl-HCH fr	HMDB0304271		Down	
beta-Alanine	HMDB0000056	C00099	Up	beta-Alanine metabolism<br>Pantothenate and CoA biosynthesis
Betaine	HMDB0000043	C00719	Up	Glycine, serine and threonine metabolism
Catechin	HMDB0002780	C06562	Down	
chiro-Inositol	HMDB0240209	C19891	Down	
Cinnamoyl acetate fr			Down	
Dehydroascorbic acid (DHAA)	HMDB0001264	C00425	Down	
Dihydrouracil	HMDB0000076	C00429	Up	beta-Alanine metabolism<br>Pantothenate and CoA biosynthesis
Dihydroxy benzoic acid fr1			Down	
Dihydroxy benzoic acid fr2			Down	
Dihydroxy benzoic acid fr3			Down	
Epigallocatechin dimer			Down	
Eriodictyol glucoside 1	HMDB0304673		Down	
Fucose	HMDB0029196	C01018	Down	
Galactinol	HMDB0005826	C01235	Down	Galactose metabolism
Gallocatechin	HMDB0038365	C12127	Down	
gamma-Aminobutyric acid (GABA)	HMDB0000112	C00334	Up	Alanine, aspartate and glutamate metabolism<br>Butanoate metabolism<br>Arginine and proline metabolism
Indoleacetaldehyde	HMDB0001190	C00637	Up	
L-Arginine	HMDB0000517	C00062	Up	Arginine biosynthesis<br>Arginine and proline metabolism
L-Asparagine	HMDB0000168	C00152	Up	Alanine, aspartate and glutamate metabolism<br>Cyanoamino acid metabolism
L-Aspartic acid	HMDB0000191	C00049	Up	Alanine, aspartate and glutamate metabolism<br>Arginine biosynthesis<br>Glycine, serine and threonine metabolism<br>beta-Alanine metabolism<br>Cyanoamino acid metabolism<br>Carbon fixation in photosynthetic organisms<br>Glycolysis / Gluconeogenesis<br>Cysteine and methionine metabolism
L-Histidine	HMDB0000177	C00135	Up	
L-Leucine / L-Isoleucine	HMDB0000687	C00123	Up	Valine, leucine and isoleucine biosynthesis
L-Lysine	HMDB0000182	C00047	Up	
L-Proline	HMDB0000162	C00148	Up	Arginine and proline metabolism
L-Serine	HMDB0000187	C00065	Up	Glycine, serine and threonine metabolism<br>Cyanoamino acid metabolism<br>Glyoxylate and dicarboxylate metabolism<br>Cysteine and methionine metabolism
L-Threonine	HMDB0000167	C00188	Up	Glycine, serine and threonine metabolism<br>Valine, leucine and isoleucine biosynthesis
LysoPE(18:2(9Z,12Z)/0:0)	HMDB0011507		Down	
Ornithine	HMDB0000214	C00077	Up	Arginine and proline metabolism<br>Arginine biosynthesis

Oxoglutaric acid	HMDB0000208	C00026	Down	Alanine, aspartate and glutamate metabolism<br>Arginine biosynthesis<br>Butanoate metabolism<br>Glyoxylate and dicarboxylate metabolism<br>TCA cycle<br>Glycolysis/Gluconeogenesis
Phosphorylcholine	HMDB0001565	C00588	Up	
Procyanidin B-type dimer 3	HMDB0033974		Down	
Procyanidin B-type trimer 3			Down	
Pyroglutamic acid	HMDB0000267	C01879	Up	
Pyruvic acid	HMDB0000243	C00022	Down	Alanine, aspartate and glutamate metabolism<br>Glycine, serine and threonine metabolism<br>Valine, leucine and isoleucine biosynthesis<br>Butanoate metabolism<br>Pantothenate and CoA biosynthesis<br>TCA cycle<br>Glycolysis/Gluconeogenesis<br>Tyrosine metabolism<br>Pyruvate metabolism<br>Carbon fixation in photosynthetic organisms<br>Cysteine and methionine metabolism
Quinic acid	HMDB0003072	C00296	Down	
Raffinose	HMDB0003213	C00492	Down	Galactose metabolism
Ribitol	HMDB0000508	C00474	Down	
Salicyl aldehyde/Benzoic acid fr 1	HMDB0001870	C00539	Down	
Salicylic acid fr 3	HMDB0001895	C00805	Down	
Shikimic acid	HMDB0003070	C00493	Down	Phenylalanine, tyrosine and tryptophan biosynthesis
Sucrose	HMDB0000258	C00089	Down	Galactose metabolism<br>Starch and sucrose metabolism<br>Glycolysis/Gluconeogenesis
Taxifolin glucopyranoside			Down	
Threonic acid	HMDB0000943	C01620	Up	
Valine	HMDB0000883	C00183	Up	Valine, leucine and isoleucine biosynthesis<br>Pantothenate and CoA biosynthesis

Table S3 Metabolites detected in Norway Spruce_south: Sun vs Shade				
**Compound Name**	**HMDB**	**KEGG**	**Direction in Shade**	**Pathway**
(±)-Taxifolin	HMDB0303943	C01617	Down	
1-Aminocyclopropanecarboxylic acid	HMDB0036458	C01234	Up	Cysteine and methionine metabolism
2,3,4,5,6,7-Hexahydroxyheptanoic acid	HMDB0240292		Down	
2-Ketobutyric acid	HMDB0000005	C00109	Down	Valine, leucine and isoleucine biosynthesis<br>Glycine, serine and threonine metabolism<br>Cysteine and methionine metabolism
Adenosine	HMDB0000050	C00212	Down	
Allothreonine	HMDB0004041	C05519	Up	Glycine, serine and threonine metabolism
Asymmetric dimethylarginine	HMDB0001539	C03626	Up	
Benzyl-HCH fr	HMDB0304271		Down	
beta-Cyanoalanine	METPA0300	C02512	Up	Cyanoamino acid metabolism
Betaine	HMDB0000043	C00719	Up	Glycine, serine and threonine metabolism
Catechin	HMDB0002780	C06562	Down	
chiro-Inositol	HMDB0240209	C19891	Down	
Cinnamoyl acetate fr			Down	
Dehydroascorbic acid (DHAA)	HMDB0001264	C00425	Down	
Dihydrouracil	HMDB0000076	C00429	Up	beta-Alanine metabolism<br>Pantothenate and CoA biosynthesis
Dihydroxy benzoic acid fr1			Down	
Dihydroxy benzoic acid fr2			Down	
Dihydroxy benzoic acid fr3			Down	
Epigallocatechin dimer			Down	
Fumaric acid	HMDB0000134	C00122	Up	Alanine, aspartate and glutamate metabolism<br>TCA cycle<br>Arginine biosynthesis<br>Tyrosine metabolism<br>Pyruvate metabolism<br>Glycolysis / Gluconeogenesis
Galactinol	HMDB0005826	C01235	Down	Galactose metabolism
Galactose 1-phosphate	HMDB0000645	C00446	Down	Galactose metabolism
Gallocatechin	HMDB0038365	C12127	Down	
gamma-Aminobutyric acid (GABA)	HMDB0000112	C00334	Up	Alanine, aspartate and glutamate metabolism<br>Butanoate metabolism<br>Arginine and proline metabolism
gamma-Glutamylleucine / gamma-Glutam	HMDB0011171		Up	
Guanosine	HMDB0000133	C00387	Down	
Isoleucine	HMDB0000172	C00407	Up	Valine, leucine and isoleucine biosynthesis
Isopropylmalate	HMDB0000402	C02504	Down	Valine, leucine and isoleucine biosynthesis<br>Pyruvate metabolism
Kaempferol	HMDB0005801	C05903	Down	Flavone and flavonol biosynthesis<br>Flavonoid biosynthesis
L-2-Hydroxyglutaric acid / 3-Hydroxyglutar	HMDB0000694	C03196	Down	
L-Arginine	HMDB0000517	C00062	Up	Arginine biosynthesis<br>Arginine and proline metabolism
L-Asparagine	HMDB0000168	C00152	Up	Alanine, aspartate and glutamate metabolism<br>Cyanoamino acid metabolism
L-Aspartic acid	HMDB0000191	C00049	Up	Alanine, aspartate and glutamate metabolism<br>Arginine biosynthesis<br>Glycine, serine and threonine metabolism<br>beta-Alanine metabolism<br>Cyanoamino acid metabolism<br>Carbon fixation in photosynthetic organisms<br>Glycolysis / Gluconeogenesis<br>Cysteine and methionine metabolism
Leucyl-Aspartate / gamma-Glutamylvaline	HMDB0028925		Up	

L-Leucine / L-Isoleucine	HMDB0000687	C00123	Up	Valine, leucine and isoleucine biosynthesis
L-Serine	HMDB0000187	C00065	Up	Glycine, serine and threonine metabolism<br>Cyanoamino acid metabolism<br>Glyoxylate and dicarboxylate metabolism<br>Cysteine and methionine metabolism
L-Threonine	HMDB0000167	C00188	Up	Glycine, serine and threonine metabolism<br>Valine, leucine and isoleucine biosynthesis
LysoPC(16:0/0:0)	HMDB0010382	C04230	Down	
LysoPC(18:2(9Z,12Z)/0:0)	HMDB0010386	C04230	Down	
LysoPC(18:3(9Z,12Z,15Z)/0:0)	HMDB0010388	C04230	Down	
LysoPE(0:0/18:2(9Z,12Z))	HMDB0011477		Down	
Malic acid	HMDB0000156	C00149	Up	TCA cycle<br>Glyoxylate and dicarboxylate metabolism<br>Pyruvate metabolism<br>Carbon fixation in photosynthetic organisms<br>Glycolysis / Gluconeogenesis
myo-Inositol	HMDB0000211	C00137	Down	Galactose metabolism<br>Inositol phosphate metabolism
Naringenin-7-O-Glucoside (Prunin)		C09099	Down	
N-gamma-Glutamylglutamine	HMDB0029147		Up	
Oxoglutaric acid	HMDB0000208	C00026	Down	Alanine, aspartate and glutamate metabolism<br>Arginine biosynthesis<br>Butanoate metabolism<br>Glyoxylate and dicarboxylate metabolism<br>TCA cycle<br>Glycolysis/Gluconeogenesis
Pantothenic acid	HMDB0000210	C00864	Up	beta-Alanine metabolism<br>Pantothenate and CoA biosynthesis
Procyanidin B-type dimer 3	HMDB0033974		Down	
Procyanidin B-type trimer 1			Down	
Procyanidin B-type trimer 3			Down	
Pyroglutamic acid	HMDB0000267	C01879	Up	
Pyruvic acid	HMDB0000243	C00022	Down	Alanine, aspartate and glutamate metabolism<br>Glycine, serine and threonine metabolism<br>Valine, leucine and isoleucine biosynthesis<br>Butanoate metabolism<br>Pantothenate and CoA biosynthesis<br>TCA cycle<br>Glycolysis/Gluconeogenesis<br>Tyrosine metabolism<br>Pyruvate metabolism<br>Carbon fixation in photosynthetic organisms
Quinic acid	HMDB0003072	C00296	Down	
Ribitol	HMDB0000508	C00474	Down	
Salicyl aldehyde/Benzoic acid fr 1	HMDB0001870	C00539	Down	
Salicylic acid fr 3	HMDB0001895	C00805	Down	
Shikimic acid	HMDB0003070	C00493	Down	Phenylalanine, tyrosine and tryptophan biosynthesis
Spermidine	HMDB0001257	C00315	Down	beta-Alanine metabolism<br>Arginine and proline metabolism
Succinic acid	HMDB0000254	C00042	Down	Alanine, aspartate and glutamate metabolism<br>Butanoate metabolism<br>Glyoxylate and dicarboxylate metabolism<br>TCA cycle<br>Glycolysis/Gluconeogenesis
Sucrose	HMDB0000258	C00089	Down	Galactose metabolism<br>Starch and sucrose metabolism<br>Glycolysis/Gluconeogenesis
Taxifolin glucopyranoside			Down	

Trigonelline	HMDB0000875	C01004	Up	
Tyrosine	HMDB0000158	C00082	Up	Phenylalanine, tyrosine and tryptophan biosynthesis<br>Tyrosine metabolism<br>Isoquinoline alkaloid biosynthesis
Valine	HMDB0000883	C00183	Up	Valine, leucine and isoleucine biosynthesis<br>Pantothenate and CoA biosynthesis
Vanillic acid fr	HMDB0000484	C06672	Down	
Vanilloyl glucoside 1			Up	

Table S4 Metabolites detected in Scots Pine_north: Sun vs Shade				
**Compound Name**	**HMDB**	**KEGG**	**Direction in Shade**	**Pathway**
(±)-Taxifolin	HMDB0303943	C01617	Down	
2,3,4,5,6,7-Hexahydroxyheptanoic acid	HMDB0240292		Down	
3-Hydroxyisovaleric acid	HMDB0000754		Up	
4-Guanidinobutanoic acid	HMDB0003464	C01035	Down	Arginine and proline metabolism
Abietic acid		C06087	Down	
Adenosine	HMDB0000050	C00212	Down	
Allothreonine	HMDB0004041	C05519	Up	Glycine, serine and threonine metabolism
Benzyl hexoside pentoside 2			Down	
Benzyl-HCH fr	HMDB0304271		Down	
beta-Cyanoalanine	METPA0300	C02512	Up	Cyanoamino acid metabolism
Betaine	HMDB0000043	C00719	Up	Glycine, serine and threonine metabolism
beta-Sitosterol	HMDB0000852	C01753	Down	
Caffeoyl glucose 3	HMDB0036937	C10433	Down	
Caffeoyl quinic acid 4	HMDB0003164	C00852/	Down	Phenylpropanoid biosynthesis<br>Flavonoid biosynthesis
Caffeoylshikimate	HMDB0304282		Down	
Campesterol	HMDB0002869	C01789	Down	
Catechin	HMDB0002780	C06562	Down	
chiro-Inositol	HMDB0240209	C19891	Down	
Cinnamoyl acetate fr			Down	
Citrulline	HMDB0000904	C00327	Up	Arginine biosynthesis
Coumaroyl quinic acid 2	HMDB0029681	C12208	Down	Phenylpropanoid biosynthesis<br>Flavonoid biosynthesis
Cyanidin 3-glucoside	HMDB0030684		Down	Anthocyanin biosynthesis
Dehydroabietate	HMDB0061925	C12078	Down	
Dehydroascorbic acid (DHAA)	HMDB0001264	C00425	Down	
Dehydromyricetin fr			Up	
Dihydrouracil	HMDB0000076	C00429	Up	beta-Alanine metabolism<br>Pantothenate and CoA biosynthesis
Dihydroxy benzoic acid fr1			Down	
Dihydroxy benzoic acid fr2			Down	
Dihydroxy benzoic acid fr3			Down	
Epigallocatechin dimer			Down	
Erythrose	HMDB0002649	C01796	Down	
Ferulic acid fr	HMDB0000954	C01494	Up	Phenylpropanoid biosynthesis
Fructose 6-phosphate	HMDB0000124	C00085	Down	Starch and sucrose metabolism<br>Pentose phosphate pathway<br>Glycolysis/Gluconeogenesis<br>Fructose and mannose metabolism
Fucose	HMDB0029196	C01018	Down	
Galactinol	HMDB0005826	C01235	Down	Galactose metabolism
Galactose 1-phosphate	HMDB0000645	C00446	Down	Galactose metabolism
Gallocatechin	HMDB0038365	C12127	Down	
gamma-Aminobutyric acid (GABA)	HMDB0000112	C00334	Up	Alanine, aspartate and glutamate metabolism<br>Butanoate metabolism<br>Arginine and proline metabolism
Gluconic acid	HMDB0000625	C00257	Down	Pentose phosphate pathway<br>Glycolysis/Gluconeogenesis
Glucose	HMDB0000122	C00221	Down	Galactose metabolism<br>Starch and sucrose metabolism<br>Glycolysis/Gluconeogenesis
Glucose 6-phosphate	HMDB0001401	C00092	Down	Starch and sucrose metabolism<br>Glycolysis/Gluconeogenesis
Glutathione (GSSG)	HMDB0003337	C00127	Down	
Indoleacetaldehyde	HMDB0001190	C00637	Up	Tryptophan metabolism

Isoleucine	HMDB0000172	C00407	Up	Valine, leucine and isoleucine biosynthesis
Isopropylmalate	HMDB0000402	C02504	Down	Valine, leucine and isoleucine biosynthesis<br>Pyruvate metabolism
Kaempferol	HMDB0005801	C05903	Down	Flavone and flavonol biosynthesis<br>Flavonoid biosynthesis
Kaempferol 3-O-b -rutinoside (Nicotiflorin)			Down	
L-Arginine	HMDB0000517	C00062	Up	Arginine biosynthesis<br>Arginine and proline metabolism
L-Asparagine	HMDB0000168	C00152	Up	Alanine, aspartate and glutamate metabolism<br>Cyanoamino acid metabolism
L-Aspartic acid	HMDB0000191	C00049	Down	Alanine, aspartate and glutamate metabolism<br>Arginine biosynthesis<br>Glycine, serine and threonine metabolism<br>beta-Alanine metabolism<br>Cyanoamino acid metabolism<br>Carbon fixation in photosynthetic organisms<br>Glycolysis / Gluconeogenesis<br>Cysteine and methionine metabolism
Leucyl-Aspartate / gamma-Glutamylvaline	HMDB0028925		Up	
L-Glutamine	HMDB0000641	C00064	Down	Alanine, aspartate and glutamate metabolism<br>Arginine biosynthesis<br>Glyoxylate and dicarboxylate metabolism
L-Histidine	HMDB0000177	C00135	Up	
Linoleic acid	HMDB0000673	C01595	Down	Linoleic acid metabolism
L-Leucine / L-Isoleucine	HMDB0000687	C00123	Up	Valine, leucine and isoleucine biosynthesis
L-Lysine	HMDB0000182	C00047	Up	
L-Phenylalanine	HMDB0000159	C00079	Up	Cyanoamino acid metabolism<br>Phenylalanine, tyrosine and tryptophan biosynthesis<br>Phenylpropanoid biosynthesis<br>Phenylalanine metabolism
L-Serine	HMDB0000187	C00065	Up	Glycine, serine and threonine metabolism<br>Cyanoamino acid metabolism<br>Glyoxylate and dicarboxylate metabolism<br>Cysteine and methionine metabolism
L-Threonine	HMDB0000167	C00188	Up	Glycine, serine and threonine metabolism<br>Valine, leucine and isoleucine biosynthesis
LysoPC(0:0/18:3)			Down	
LysoPC(16:0/0:0)	HMDB0010382	C04230	Down	
LysoPC(18:1(9Z)/0:0)	HMDB0002815	C04230	Down	
LysoPC(18:2(9Z,12Z)/0:0)	HMDB0010386	C04230	Down	
LysoPE(0:0/18:2(9Z,12Z))	HMDB0011477	C04230	Down	
LysoPE(16:0/0:0)	HMDB0011503		Down	
Oxoglutaric acid	HMDB0000208	C00026	Down	Alanine, aspartate and glutamate metabolism<br>Arginine biosynthesis<br>Butanoate metabolism<br>Glyoxylate and dicarboxylate metabolism<br>TCA cycle<br>Glycolysis/Gluconeogenesis
Palatinose		C01742	Down	
Palmitic acid	HMDB0000220	C00249	Down	
Pelargonic acid	HMDB0000847	C01601	Up	
Phosphorylcholine	HMDB0001565	C00588	Up	
Procyanidin B-type dimer 1	HMDB0029754		Down	
Procyanidin B-type dimer 2	HMDB0033973		Down	
Procyanidin B-type dimer 3	HMDB0033974		Down	
Procyanidin B-type trimer 1			Down	
Procyanidin B-type trimer 2			Down	

Protocatechuic acid glucoside	HMDB0303826		Up	
Pyruvic acid	HMDB0000243	C00022	Down	Alanine, aspartate and glutamate metabolism<br>Glycine, serine and threonine metabolism<br>Valine, leucine and isoleucine biosynthesis<br>Butanoate metabolism<br>Pantothenate and CoA biosynthesis<br>TCA cycle<br>Glycolysis/Gluconeogenesis<br>Tyrosine metabolism<br>Pyruvate metabolism<br>Carbon fixation in photosynthetic organisms
Quercetin fr 2			Up	
Quinic acid	HMDB0003072	C00296	Down	
Ribitol	HMDB0000508	C00474	Down	
Salicyl aldehyde/Benzoic acid fr 1	HMDB0001870	C00539	Down	
Salicylic acid fr 3	HMDB0001895	C00805	Down	
Salirepin 1			Down	
Shikimic acid	HMDB0003070	C00493	Down	Phenylalanine, tyrosine and tryptophan biosynthesis
Spermidine	HMDB0001257	C00315	Down	beta-Alanine metabolism<br>Arginine and proline metabolism
Succinic acid	HMDB0000254	C00042	Down	Alanine, aspartate and glutamate metabolism<br>Butanoate metabolism<br>Glyoxylate and dicarboxylate metabolism<br>TCA cycle<br>Glycolysis/Gluconeogenesis
Sucrose	HMDB0000258	C00089	Down	Galactose metabolism<br>Starch and sucrose metabolism<br>Glycolysis/Gluconeogenesis
Threonic acid	HMDB0000943	C01620	Up	
Tryptophan	HMDB0000929	C00078	Up	Glycine, serine and threonine metabolism<br>Phenylalanine, tyrosine and tryptophan biosynthesis<br>Tryptophan metabolism
Tyrosine	HMDB0000158	C00082	Up	Phenylalanine, tyrosine and tryptophan biosynthesis<br>Tyrosine metabolism<br>Isoquinoline alkaloid biosynthesis
Valine	HMDB0000883	C00183	Up	Valine, leucine and isoleucine biosynthesis<br>Pantothenate and CoA biosynthesis
Xanthone der 1	HMDB0029463	C10065	Up	

Table S5 Metabolites detected in Scots Pine_south: Sun vs Shade				
**Compound Name**	**HMDB**	**KEGG**	**Direction in Shade**	**Pathway**
(±)-Taxifolin	HMDB0303943	C01617	Down	
2,3,4,5,6,7-Hexahydroxyheptanoic ac	HMDB0240292		Down	
2-Hydroxyhexadecanoic acid	HMDB0031057		Down	
3-Hydroxyisovaleric acid	HMDB0000754		Up	
4-Guanidinobutanoic acid	HMDB0003464	C01035	Down	Arginine and proline metabolism
5-Hydroxy-L-tryptophan	HMDB0000472	C00643	Up	Tryptophan metabolism
Abietic acid		C06087	Down	
Allothreonine	HMDB0004041	C05519	Up	Glycine, serine and threonine metabolism
Asymmetric dimethylarginine	HMDB0001539	C03626	Up	
Benzyl-HCH fr	HMDB0304271		Down	
beta-Cyanoalanine	METPA0300	C02512	Up	Cyanoamino acid metabolism
Betaine	HMDB0000043	C00719	Up	Glycine, serine and threonine metabolism
Caffeoyl glucose 3	HMDB0036937	C10433	Down	
Caffeoyl quinic acid 4	HMDB0003164	C00852/(	Down	Phenylpropanoid biosynthesis<br>Flavonoid biosynthesis
Caffeoylshikimate	HMDB0304282		Down	
Catechin	HMDB0002780	C06562	Down	
chiro-Inositol	HMDB0240209	C19891	Down	
Cinnamoyl acetate fr			Down	
Citrulline	HMDB0000904	C00327	Up	Arginine biosynthesis
Coumaroyl quinic acid 2	HMDB0029681	C12208	Down	Phenylpropanoid biosynthesis<br>Flavonoid biosynthesis
Coumaroyl quinic acid 3	HMDB0301709	C12208	Down	Phenylpropanoid biosynthesis<br>Flavonoid biosynthesis
Dehydroabietate	HMDB0061925	C12078	Down	
Dehydroascorbic acid (DHAA)	HMDB0001264	C00425	Down	
D-Glucuronic acid	HMDB0000127	C00191	Down	Pentose and glucuronate interconversions
Dihydrouracil	HMDB0000076	C00429	Up	beta-Alanine metabolism<br>Pantothenate and CoA biosynthesis
Dihydroxy benzoic acid fr1			Down	
Dihydroxy benzoic acid fr2			Down	
Dihydroxy benzoic acid fr3			Down	
Dimethoxybenzaldehyde fr 2			Down	
D-threo-Isocitric acid	HMDB0001874	C00451	Down	
Epigallocatechin dimer			Down	
Ferulic acid fr	HMDB0000954	C01494	Up	Phenylpropanoid biosynthesis
Fructose	HMDB0000660	C02336	Down	Galactose metabolism<br>Starch and sucrose metabolism<br>Glycolysis/Gluconeogenesis<br>Fructose and mannose metabolism
Fucose	HMDB0029196	C01018	Down	
Galactinol	HMDB0005826	C01235	Down	Galactose metabolism
Galactose 1-phosphate	HMDB0000645	C00446	Down	Galactose metabolism
Gallocatechin	HMDB0038365	C12127	Down	
gamma-Aminobutyric acid (GABA)	HMDB0000112	C00334	Up	Alanine, aspartate and glutamate metabolism<br>Butanoate metabolism<br>Arginine and proline metabolism
gamma-Glutamylleucine / gamma-Gl	HMDB0011171		Up	
Gluconic acid	HMDB0000625	C00257	Down	
Glucose	HMDB0000122	C00221	Down	Galactose metabolism<br>Starch and sucrose metabolism<br>Glycolysis/Gluconeogenesis

Indoleacetaldehyde	HMDB0001190	C00637	Up	Tryptophan metabolism
Isopropylmalate	HMDB0000402	C02504	Down	Valine, leucine and isoleucine biosynthesis<br>Pyruvate metabolism
Kaempferol	HMDB0005801	C05903	Down	Flavone and flavonol biosynthesis<br>Flavonoid biosynthesis
Kaempferol 3-O-b -rutinoside (Nicotiflorin)			Down	
Kaempferol-7-o-glucoside			Down	
Kaempherol fr			Down	
L-Arginine	HMDB0000517	C00062	Up	Arginine biosynthesis<br>Arginine and proline metabolism
L-Asparagine	HMDB0000168	C00152	Up	Alanine, aspartate and glutamate metabolism<br>Cyanoamino acid metabolism
L-Aspartic acid	HMDB0000191	C00049	Down	Alanine, aspartate and glutamate metabolism<br>Arginine biosynthesis<br>Glycine, serine and threonine metabolism<br>beta-Alanine metabolism<br>Cyanoamino acid metabolism<br>Carbon fixation in photosynthetic organisms<br>Glycolysis / Gluconeogenesis<br>Cysteine and methionine metabolism
Leucyl-Aspartate / gamma-Glutamylv	HMDB0028925		Up	
L-Glutamine	HMDB0000641	C00064	Down	Alanine, aspartate and glutamate metabolism<br>Arginine biosynthesis<br>Glyoxylate and dicarboxylate metabolism
L-Histidine	HMDB0000177	C00135	Up	
L-Leucine / L-Isoleucine	HMDB0000687	C00123	Up	Valine, leucine and isoleucine biosynthesis
L-Lysine	HMDB0000182	C00047	Up	
L-Phenylalanine	HMDB0000159	C00079	Up	Cyanoamino acid metabolism<br>Phenylalanine, tyrosine and tryptophan biosynthesis<br>Phenylpropanoid biosynthesis<br>Phenylalanine metabolism
L-Serine	HMDB0000187	C00065	Up	Glycine, serine and threonine metabolism<br>Cyanoamino acid metabolism<br>Glyoxylate and dicarboxylate metabolism<br>Cysteine and methionine metabolism
L-Threonine	HMDB0000167	C00188	Up	Glycine, serine and threonine metabolism<br>Valine, leucine and isoleucine biosynthesis
LysoPC(16:0/0:0)	HMDB0010382	C04230	Down	
LysoPC(18:1(9Z)/0:0)	HMDB0002815	C04230	Down	
LysoPC(18:2(9Z,12Z)/0:0)	HMDB0010386	C04230	Down	
LysoPE(0:0/18:2(9Z,12Z))	HMDB0011477		Down	
LysoPE(16:0/0:0)	HMDB0011503		Down	
myo-Inositol	HMDB0000211	C00137	Down	Galactose metabolism
Myo-Inositol 1-Monophosphate	HMDB0000213	C01177	Down	
N-gamma-Glutamylglutamine	HMDB0029147		Down	
Ornithine	HMDB0000214	C00077	Up	Arginine biosynthesis<br>Arginine and proline metabolism
Oxoglutaric acid	HMDB0000208	C00026	Down	Alanine, aspartate and glutamate metabolism<br>Arginine biosynthesis<br>Butanoate metabolism<br>Glyoxylate and dicarboxylate metabolism<br>TCA cycle<br>Glycolysis/Gluconeogenesis

Pantothenic acid	HMDB0000210	C00864	Up	beta-Alanine metabolism<br>Pantothenate and CoA biosynthesis
Pelargonic acid	HMDB0000847	C01601	Up	
Phosphorylcholine	HMDB0001565	C00588	Up	
Procyanidin B-type dimer 1	HMDB0029754		Down	
Procyanidin B-type dimer 2	HMDB0033973		Down	
Procyanidin B-type dimer 3	HMDB0033974		Down	
Procyanidin B-type trimer 1			Down	
Procyanidin B-type trimer 2			Down	
Pyroglutamic acid	HMDB0000267	C01879	Down	
Pyruvic acid	HMDB0000243	C00022	Down	Alanine, aspartate and glutamate metabolism<br>Glycine, serine and threonine metabolism<br>Valine, leucine and isoleucine biosynthesis<br>Butanoate metabolism<br>Pantothenate and CoA biosynthesis<br>TCA cycle<br>Glycolysis/Gluconeogenesis<br>Tyrosine metabolism<br>Pyruvate metabolism<br>Carbon fixation in photosynthetic organisms
Quinic acid	HMDB0003072	C00296	Down	
Ribitol	HMDB0000508	C00474	Down	
Salicyl aldehyde/Benzoic acid fr 1	HMDB0001870	C00539	Down	
Salicylic acid fr 3	HMDB0001895	C00805	Down	
Salirepin 1			Down	
Shikimic acid	HMDB0003070	C00493	Down	Phenylalanine, tyrosine and tryptophan biosynthesis
Spermidine	HMDB0001257	C00315	Down	beta-Alanine metabolism<br>Arginine and proline metabolism
Succinic acid	HMDB0000254	C00042	Down	Alanine, aspartate and glutamate metabolism<br>Butanoate metabolism<br>Glyoxylate and dicarboxylate metabolism<br>TCA cycle<br>Glycolysis/Gluconeogenesis
Sucrose	HMDB0000258	C00089	Down	Galactose metabolism<br>Starch and sucrose metabolism<br>Glycolysis/Gluconeogenesis
Trigonelline	HMDB0000875	C01004	Up	
Tryptophan	HMDB0000929	C00078	Up	Glycine, serine and threonine metabolism<br>Phenylalanine, tyrosine and tryptophan biosynthesis<br>Tryptophan metabolism
Tyrosine	HMDB0000158	C00082	Up	Phenylalanine, tyrosine and tryptophan biosynthesis<br>Tyrosine metabolism<br>Isoquinoline alkaloid biosynthesis
Valine	HMDB0000883	C00183	Up	Valine, leucine and isoleucine biosynthesis<br>Pantothenate and CoA biosynthesis
Vanillic acid fr	HMDB0000484	C06672	Down	

Table S6 Details of PAL sequences from monocots and putative PAL sequences from conifers used for the multiple alignment and phylogeny.

Species	Sequence Id (TAIR/GenBank/Plaza)	Expression: Sun Versus Shade at South and North, respectively	Number of amino acids	Abbreviation
Arabidopsis thaliana	AT2G37040		725	AtPAL
Brachypodium distachyon	XP_003575400.1		717	BdPAL
Bambusa oldhamii	ACN62413.1		713	BoPAL
Petroselinum crispum	P24481.1		716	PcPAL
Populus trichocarpa	XP_006381441.1		714	PtPAL
Oryza sativa	A2X7F7.1		713	OsPAL
Zea mays	NP_001151482.2		718	ZmPAL
Picea abies	PAB00008810/MA_10429279g0010	Not significant at latitude South and North	711	PabPAL1
	PAB00025303/MA_15852g0010 High confidence	Not significant at latitude South Shade>Sun at latitude North	787	PabPAL2
Pseudotsuga menziesii	PME00016510		712	PmePAL1
	PME00011612		828	PmePAL2
Picea sitchensis	PSI00008374		790	PsiPAL
Pinus sylvestris	PSY00008254		678	PsyPAL
Pinus taeda	PTA00029770/ PITA_000038676	Not significant at latitude South and North	711	PtaPAL1
	PTA00066008/ PITA_000078355	Shade<Sun at latitude South Not significant at latitude North	711	PtaPAL2
	PTA00062798/ PITA_000074853	Not significant at latitude South and North	739	PtaPAL3
	PTA00046911/ PITA_000057642	Not significant at latitude South and North	696	PtaPAL4

Table S7 Details of PTAL sequences from monocots and putative BAL sequences from conifers used for the multiple alignment and phylogeny.

Species	Sequence Id (TAIR/GenBank/Plaza)	Expression: Sun Versus Shade at South and North, respectively	Number of amino acids	Abbreviation
Brachypodium distachyon	XP_003575396.1		707	BdPTAL
Bambusa oldhamii	ADE08261.1		701	BoPTAL
Oryza sativa	S06475		701	OsPTAL
Zea mays	NP_001105334.2		703	ZmPTAL
Picea abies	PAB00020676/MA_123220g0010	Not significant at latitude South and North	748	PabBAL1
	PAB00042267/MA_44561g0010 High confidence	Shade<Sun at latitude South and North	718	PabBAL2
	PAB00052998/MA_73113g0010	Not significant at latitude South and North	736	PabBAL3
Pseudotsuga menziesii	PME00018594		753	PmeBAL1
	PME00002674		724	PmeBAL2
	PME00065720		765	PmeBAL3
	PME00143027		687	PmeBAL4
Picea sitchensis	PSI00019215		720	PsiBAL
Pinus sylvestris	PSY00017079		718	PsyBAL
Pinus taeda	PTA00031900/ PITA_000041078	Not significant at latitude South and North	718	PtaBAL1
	PTA00063025/ PITA_000075110	Not significant at latitude South and North	764	PtaBAL2
	PTA00005492/ PITA_000005593	Not expressed at latitude South and North	686	PtaBAL3

Table S8 Population-wise allele frequencies of putative PAL/BAL SNPs detected in Norway spruce										
**Gene<br>Spruce gene id**	**Position**	**Mutation**	**Allele**	**Population-wise allele frequency**						**Cline**
				**S1**	**S2**	**S3**	**S4**	**S5**	**S6**	
**PabPAL2<br>MA_15852g0010**	35746	missense	Reference (C)	0.59	0.58	0.60	0.57	0.58	0.56	No
			Alternate (T)	0.41	0.42	0.40	0.43	0.42	0.44	
	35760	missense	Reference (A)	0.56	0.56	0.56	0.57	0.57	0.55	No
			Alternate (T)	0.44	0.44	0.44	0.43	0.43	0.45	
	35766	missense	Reference (T)	0.56	0.56	0.57	0.57	0.56	0.56	No
			Alternate (G)	0.44	0.44	0.43	0.43	0.44	0.44	
	35776	missense	Reference (C)	0.56	0.56	0.58	0.56	0.57	0.56	No
			Alternate (A)	0.44	0.44	0.43	0.44	0.43	0.44	
	35780	synonymous	Reference (C)	0.58	0.57	0.58	0.59	0.58	0.59	No
			Alternate (T)	0.42	0.43	0.42	0.41	0.42	0.41	
	35829	missense	Reference (T)	0.52	0.52	0.53	0.52	0.52	0.52	No
			Alternate (G)	0.48	0.48	0.47	0.48	0.48	0.48	
	35893	missense	Reference (C)	0.82	0.81	0.81	0.82	0.87	0.83	No
			Alternate (T)	0.18	0.19	0.19	0.18	0.13	0.17	
	35924	synonymous	Reference (A)	0.51	0.51	0.51	0.52	0.52	0.51	No
			Alternate (G)	0.49	0.49	0.49	0.48	0.48	0.49	
	35929	missense	Reference (A)	0.50	0.50	0.50	0.50	0.50	0.50	No
			Alternate (T)	0.50	0.50	0.50	0.50	0.50	0.50	
	35940	synonymous	Reference (C)	1.00	0.98	0.99	1.00	0.98	0.98	No
			Alternate (A)	0.00	0.02	0.01	0.00	0.02	0.02	
	**36047**	**synonymous**	Reference (T)	**0.50**	**0.50**	**0.50**	**0.49**	**0.48**	**0.47**	**Yes**
			Alternate (C)	**0.50**	**0.50**	**0.50**	**0.51**	**0.52**	**0.53**	
	36124	missense	Reference (G)	0.93	0.94	0.90	0.93	0.93	0.94	No
			Alternate (A)	0.07	0.06	0.10	0.07	0.07	0.06	
	36127	missense	Reference (A)	1.00	1.00	0.99	0.93	0.92	0.95	No
			Alternate (G)	0.00	0.00	0.01	0.07	0.08	0.05	
	36132	missense	Reference (G)	0.43	0.42	0.43	0.44	0.42	0.41	No
			Alternate (A)	0.57	0.58	0.57	0.56	0.58	0.59	
	36150	missense	Reference (A)	0.56	0.56	0.58	0.57	0.60	0.56	No
			Alternate (C)	0.44	0.44	0.42	0.43	0.40	0.44	
	36176	synonymous	Reference (T)	0.98	0.99	0.99	0.99	0.99	1.00	No
			Alternate (A)	0.02	0.01	0.01	0.01	0.01	0.00	
	36193	missense	Reference (G)	0.66	0.69	0.69	0.67	0.69	0.65	No
			Alternate (C)	0.34	0.31	0.31	0.33	0.31	0.35	
	36197	synonymous	Reference (G)	0.66	0.67	0.68	0.67	0.69	0.64	No
			Alternate (A)	0.34	0.33	0.32	0.33	0.31	0.36	
	36230	synonymous	Reference (T)	0.74	0.75	0.79	0.74	0.74	0.68	No
			Alternate (C)	0.26	0.25	0.21	0.26	0.26	0.32	
	36236	synonymous	Reference (T)	0.78	0.80	0.84	0.81	0.77	0.72	No
			Alternate (C)	0.22	0.20	0.16	0.19	0.23	0.28	
	36257	synonymous	Reference (G)	0.87	0.89	0.86	0.82	0.83	0.76	No
			Alternate (A)	0.13	0.11	0.14	0.18	0.17	0.24	
	36338	synonymous	Reference (C)	0.79	0.81	0.77	0.70	0.72	0.68	No
			Alternate (T)	0.21	0.19	0.23	0.30	0.28	0.32	
	36347	synonymous	Reference (A)	0.40	0.40	0.40	0.42	0.43	0.40	No
			Alternate (G)	0.60	0.60	0.60	0.58	0.57	0.60	
	36356	synonymous	Reference (C)	0.78	0.80	0.78	0.70	0.70	0.68	No
			Alternate (T)	0.22	0.20	0.22	0.30	0.30	0.32	

Gene<br>Spruce gene id	Position	Mutation	Allele	Population-wise allele frequency<br>S1	S2	S3	S4	S5	S6	Cline
**PabPAL2**<br>**MA_15852g0010**	36380	synonymous	Reference (G)	0.50	0.51	0.53	0.51	0.52	0.50	No
			Alternate (C)	0.50	0.49	0.47	0.49	0.48	0.50	
	**36431**	**synonymous**	Reference (G)	**0.97**	**0.96**	**0.96**	**0.95**	**0.92**	**0.86**	**Yes**
			Alternate (C)	**0.03**	**0.04**	**0.04**	**0.05**	**0.08**	**0.14**	
	36440	synonymous	Reference (A)	0.50	0.51	0.50	0.50	0.50	0.51	No
			Alternate (G)	0.50	0.49	0.50	0.50	0.50	0.49	
	36446	synonymous	Reference (A)	0.50	0.51	0.50	0.50	0.50	0.51	No
			Alternate (G)	0.50	0.49	0.50	0.50	0.50	0.49	
	36497	synonymous	Reference (C)	0.50	0.50	0.50	0.50	0.50	0.50	No
			Alternate (T)	0.50	0.50	0.50	0.50	0.50	0.50	
	**36510**	**missense**	Reference (G)	**0.90**	**0.90**	**0.87**	**0.86**	**0.85**	**0.77**	**Yes**
			Alternate (A)	**0.10**	**0.10**	**0.13**	**0.14**	**0.15**	**0.23**	
	36512	synonymous	Reference (A)	0.50	0.50	0.50	0.50	0.50	0.50	No
			Alternate (C)	0.50	0.50	0.50	0.50	0.50	0.50	
	36561	synonymous	Reference (T)	0.89	0.94	0.94	0.96	0.95	0.95	No
			Alternate (C)	0.11	0.06	0.06	0.04	0.05	0.05	
	36677	synonymous	Reference (C)	0.83	0.82	0.77	0.86	0.90	0.85	No
			Alternate (T)	0.17	0.18	0.23	0.14	0.10	0.15	
	36699	missense	Reference (T)	0.98	0.95	0.96	0.96	0.97	0.98	No
			Alternate (A)	0.02	0.05	0.04	0.04	0.03	0.02	
**PabBAL1**<br>**MA_123220g0010**	3693	synonymous	Reference (C)	0.99	0.98	1.00	0.99	1.00	0.98	No
			Alternate (A)	0.01	0.02	0.00	0.01	0.00	0.02	
	**3784**	**missense**	Reference (C)	**0.97**	**0.97**	**0.95**	**0.93**	**0.89**	**0.86**	**Yes**
			Alternate (T)	**0.03**	**0.03**	**0.05**	**0.07**	**0.11**	**0.14**	
	**3790**	**missense**	Reference (C)	**0.90**	**0.90**	**0.87**	**0.88**	**0.84**	**0.80**	**Yes**
			Alternate (T)	**0.10**	**0.10**	**0.13**	**0.12**	**0.16**	**0.20**	
	3805	missense	Reference (C)	0.94	0.96	0.94	0.95	0.95	0.96	No
			Alternate (T)	0.06	0.04	0.06	0.05	0.05	0.04	
	3807	synonymous	Reference (C)	0.96	0.96	0.97	0.99	0.98	0.98	No
			Alternate (T)	0.04	0.04	0.03	0.01	0.02	0.02	
	3831	synonymous	Reference (G)	0.96	0.96	0.97	0.99	0.98	0.98	No
			Alternate (A)	0.04	0.04	0.03	0.01	0.02	0.02	
	3861	synonymous	Reference (C)	0.98	0.97	0.96	0.94	0.87	0.93	No
			Alternate (T)	0.02	0.03	0.04	0.06	0.13	0.08	
	3882	synonymous	Reference (C)	0.99	0.99	0.99	1.00	0.99	0.98	No
			Alternate (T)	0.01	0.01	0.01	0.00	0.01	0.02	
**PabBAL3**<br>**MA_73113g0010**	6025	missense	Reference (A)	0.93	0.96	0.94	0.99	0.98	0.97	No
			Alternate (G)	0.07	0.04	0.06	0.01	0.02	0.03	
	6122	missense	Reference (G)	0.92	0.91	0.86	0.76	0.81	0.71	No
			Alternate (A)	0.08	0.09	0.14	0.24	0.19	0.29	
	6168	synonymous	Reference (G)	0.84	0.87	0.88	0.94	0.93	0.91	No
			Alternate (A)	0.16	0.13	0.12	0.06	0.07	0.09	
	6237	synonymous	Reference (G)	0.84	0.83	0.82	0.84	0.84	0.81	No
			Alternate (A)	0.16	0.17	0.18	0.16	0.16	0.19	
	**6265**	**missense**	Reference (C)	**0.94**	**0.95**	**0.91**	**0.87**	**0.89**	**0.83**	**Yes**
			Alternate (T)	**0.06**	**0.05**	**0.09**	**0.13**	**0.11**	**0.17**	
	**6296**	**missense**	Reference (A)	**0.95**	**0.95**	**0.92**	**0.89**	**0.89**	**0.85**	**Yes**
			Alternate (T)	**0.05**	**0.05**	**0.08**	**0.11**	**0.11**	**0.15**	

Table S9 P-values from one-way ANOVA of the allele frequencies and genotype frequencies of missense SNPs in putative PAL/BAL showing cline across the populations in Norway spruce in Sweden

Gene<br>Spruce gene id	Variation	Reference allele frequency p-value	Alternate allele frequency p-value	Genotype frequency p-value
**PabPAL2**<br>**MA_15852g0010**	Reference T, alternate C<br>GCT → GCC; Ala228Ala	T: 3.19E-05	C: 0.0004	0.0002
	Reference G, alternate C<br>CCG → CCC; Pro356Pro	G: 3.99E-12	C: 3.99E-12	3.99E-12
	Reference G, alternate A<br>GCA → ACA; Ala383Thr	G: 4.75E-10	A: 4.75E-10	4.75E-10
**PabBAL1**<br>**MA_123220g0010**	Reference C, alternate T<br>GCC → GTC; Ala535Val	C: 3.34E-06	T: 1.43E-06	1.73E-06
	Reference C, alternate T<br>GCT → GCT; Ala537Val	C: 7.8E-15	T: 1.56E-15	1.47E-15
**PabBAL3**<br>**MA_73113g0010**	Reference C, alternate T<br>TCC → TTC; Ser595Phe	C: 3.09E-06	T: 1.3E-09	4.12E-09
	Reference A, alternate T<br>TTA → TTT; Leu601Phe	A: 1.95E-06	T: 7.36E-09	1.20E-07

Table S10 One-way ANOVA and Tukey's post-hoc test for genotype frequencies of missense SNPs showing latitudinal cline in Norway spruce in Sweden.
Significant p-values (p-value<0.05) are highlighted

Gene<br>Spruce gene id	Variation	Tukey's p-value S1 vs S2	Tukey's p-value S1 vs S3	Tukey's p-value S1 vs S4	Tukey's p-value S1 vs S5	Tukey's p-value S1 vs S6	Tukey's p-value S2 vs S3	Tukey's p-value S2 vs S4	Tukey's p-value S2 vs S5	Tukey's p-value S2 vs S6	Tukey's p-value S3 vs S4	Tukey's p-value S3 vs S5	Tukey's p-value S3 vs S6	Tukey's p-value S4 vs S5	Tukey's p-value S4 vs S6	Tukey's p-value S5 vs S6
**PabPAL2**<br>**MA_15852g0010**	Ala228Ala	0.89999	0.89999	0.89999	0.14764	**0.0016**	0.89999	0.89999	0.38736	**0.0085**	0.89493	0.13527	**0.0018**	0.77999	**0.0491**	0.24361
	Pro356Pro	0.89999	0.89999	0.80186	**0.0014**	**0.001**	0.89999	0.89999	**0.0358**	**0.001**	0.89999	**0.033**	**0.001**	0.20479	**0.001**	**0.001**
	Ala383Thr	0.89999	0.7354	0.42999	0.10567	**0.001**	0.73089	0.43676	0.12345	**0.001**	0.89999	0.89999	**0.001**	0.89999	**0.001**	**0.001**
**PabBAL1**<br>**MA_123220g0010**	Ala535Val	0.89999	0.52257	0.79511	**0.0041**	**0.001**	0.62482	0.89213	**0.0131**	**0.001**	0.89999	0.74499	0.06147	0.35208	**0.01**	0.30369
	Ala537Val	0.89999	0.79039	0.10416	**0.001**	**0.001**	0.89999	0.27973	**0.001**	**0.001**	0.80937	**0.0018**	**0.001**	0.12789	**0.001**	0.13467
**PabBAL3**<br>**MA_73113g0010**	Ser595Phe	0.89999	0.68176	**0.0082**	**0.0059**	**0.001**	0.64761	**0.0086**	**0.0072**	**0.001**	0.4482	0.61754	**0.0015**	0.89999	0.30079	**0.0165**
	Leu601Phe	0.89999	0.43629	**0.0123**	**0.0024**	**0.001**	0.47522	**0.018**	**0.0049**	**0.001**	0.74936	0.76679	**0.0369**	0.89999	0.54461	0.17789
