# Supplementary material for: Metabolomic profiling of shade response and in silico analysis of PAL homologs imply the potential presence of bifunctional ammonia lyases in conifers
Source: Physiol Plant. 2025 Mar 27;177(2):e70175. doi: 10.1111/ppl.70175 (PMC11949857; doi:10.1111/ppl.70175)
Supplement: Supplementary file 4 — Supplementary file4. [file PPL-177-e70175-s004.pdf]

OPLS-DA: **R2Y** is the model interpretation rate; **Q2Y** is the model predictive ability. **R2Y** and **Q2Y** closer to 1 indicates the model stability and reliability.
K is the number metabolites considered for the plot.
Regression: **R2** is the goodness-of-fit of the linear regression.

Figure S1 Norway Spruce, LCMS data: PCA shows separation between all groups – North_Shade, North_Sun, South_Shade and South_Sun; targeted + untargeted metabolites, K = 799.

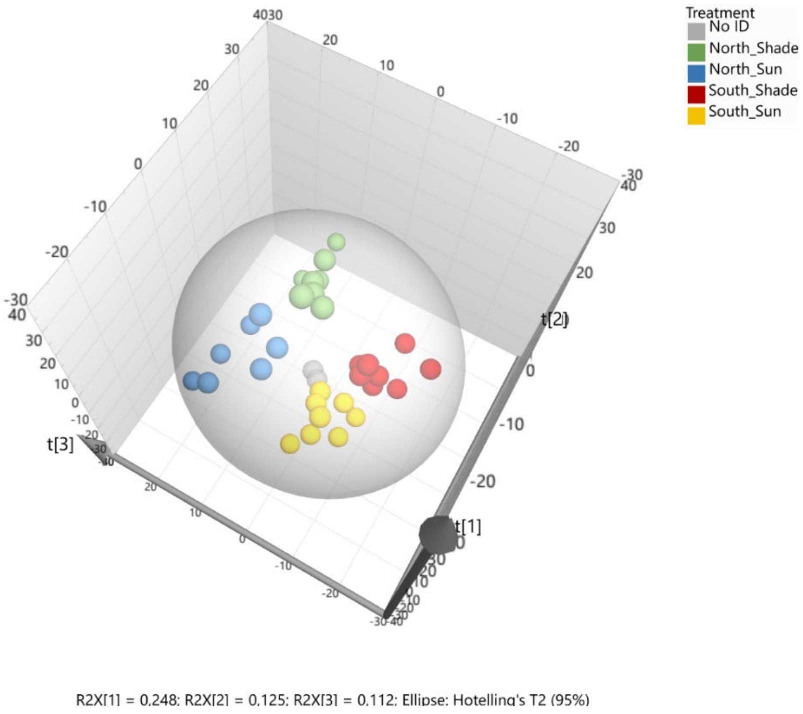

Figure S2 Norway Spruce GCMS data: PCA shows separation between all groups – North_Shade, North_Sun, South_Shade and South_Sun; K = 69.

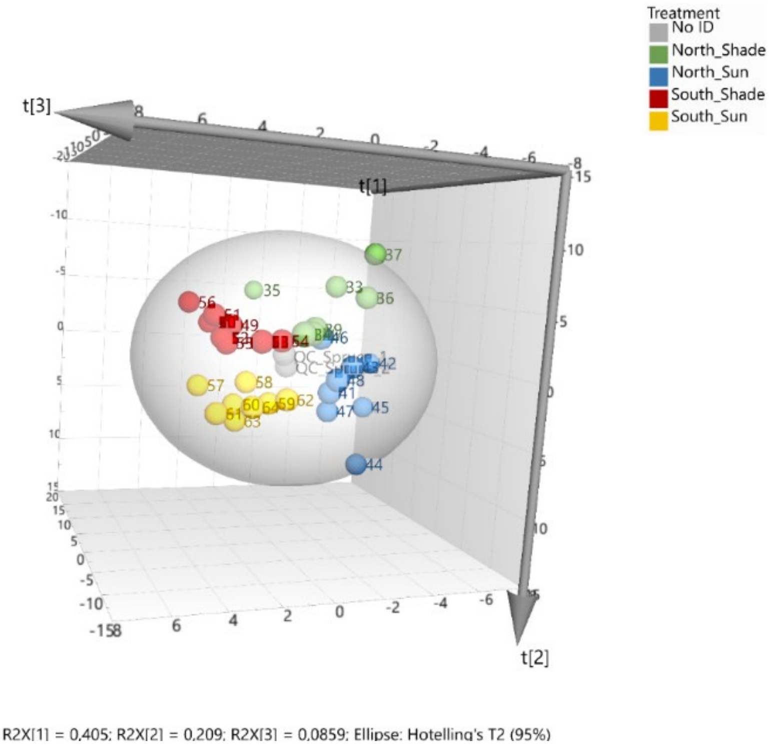

Figure S3 Scots pine LCMS data: PCA shows separation between all groups – North_Shade, North_Sun, South_Shade and South_Sun; targeted + untargeted metabolites, K = 781.

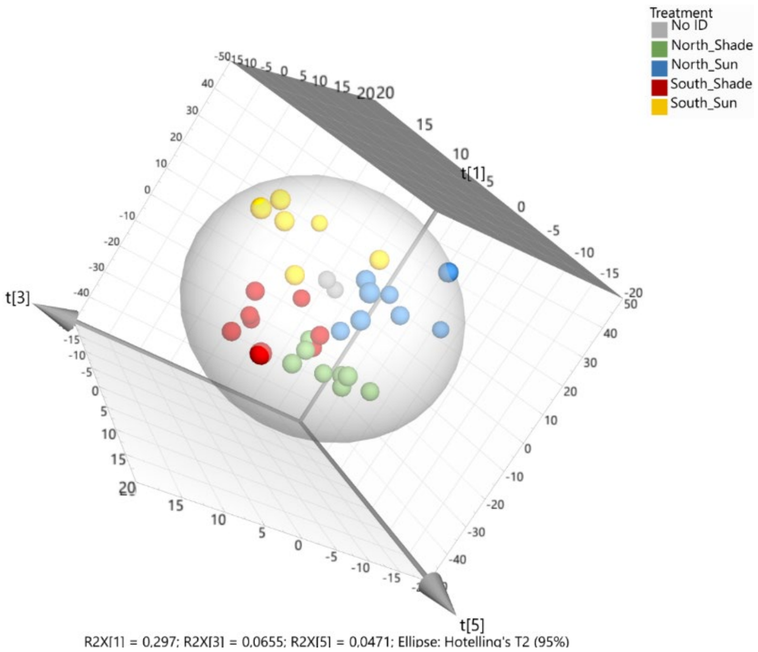

Figure S4 Scots pine GCMS data: PCA shows clear separation between Shade and Sun conditions, but the Northern and Southern samples are not completely separated; K = 68.

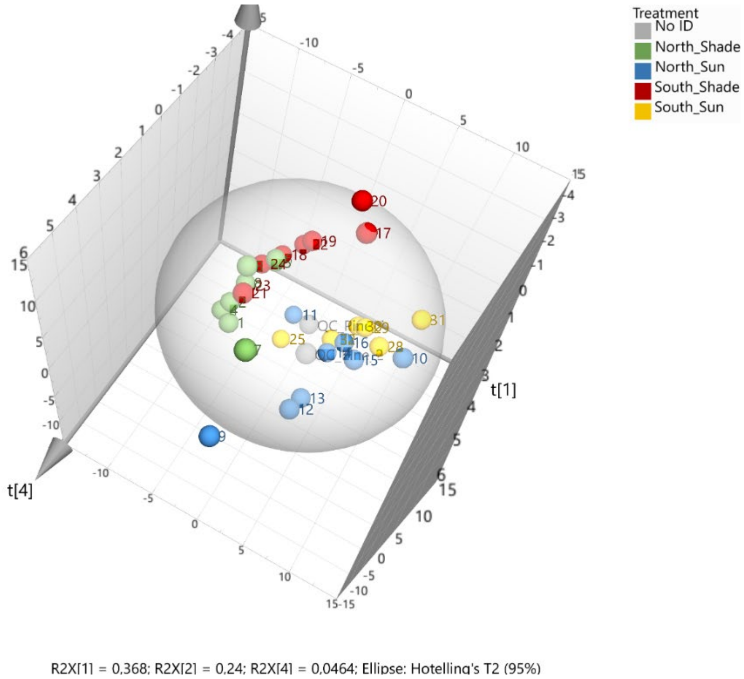

Figure S5 Norway spruce samples, LCMS data: OPLS-DA shows differences between Shade and Sun conditions in both populations; targeted + untargeted metabolites, K = 799.

(a) Northern samples: R2Y = 0.97 Q2Y = 0.78

(b) Southern samples: R2Y = 0.98, Q2Y = 0.84

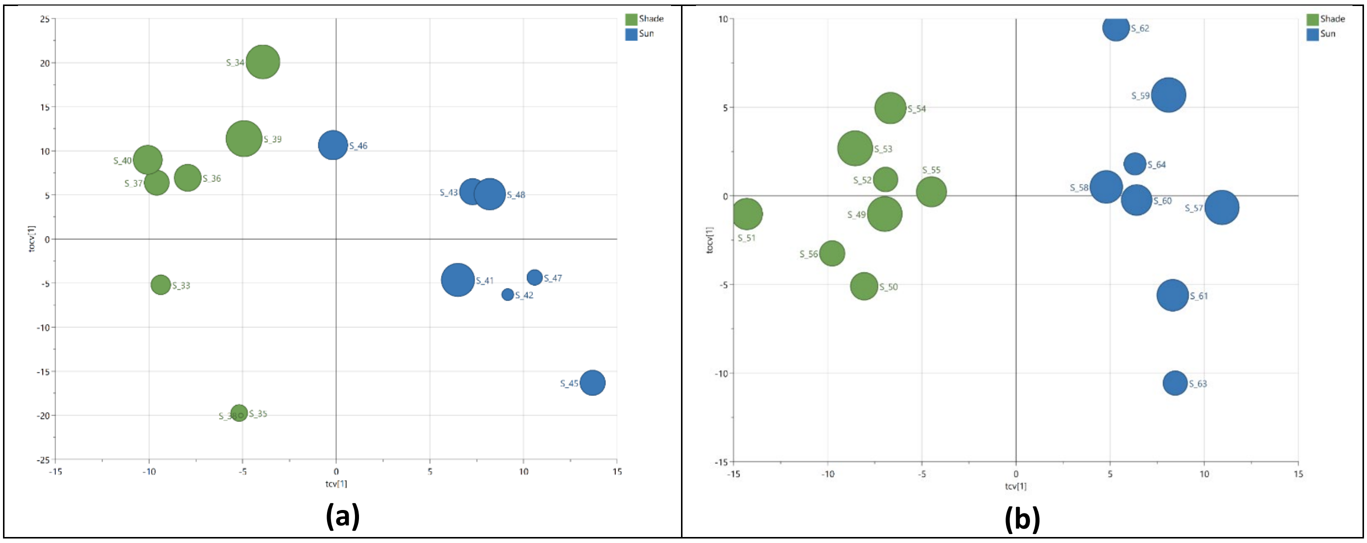

Figure S6 Norway spruce samples, GCMS data: OPLS-DA shows differences between Shade and Sun conditions in both populations; K = 69.

(a) Northern samples: Clear separation between Shade and Sun conditions for all samples except S46. R2Y = 0.79 Q2Y = 0.58

(b) Southern samples: R2Y = 0.97, Q2Y = 0.94

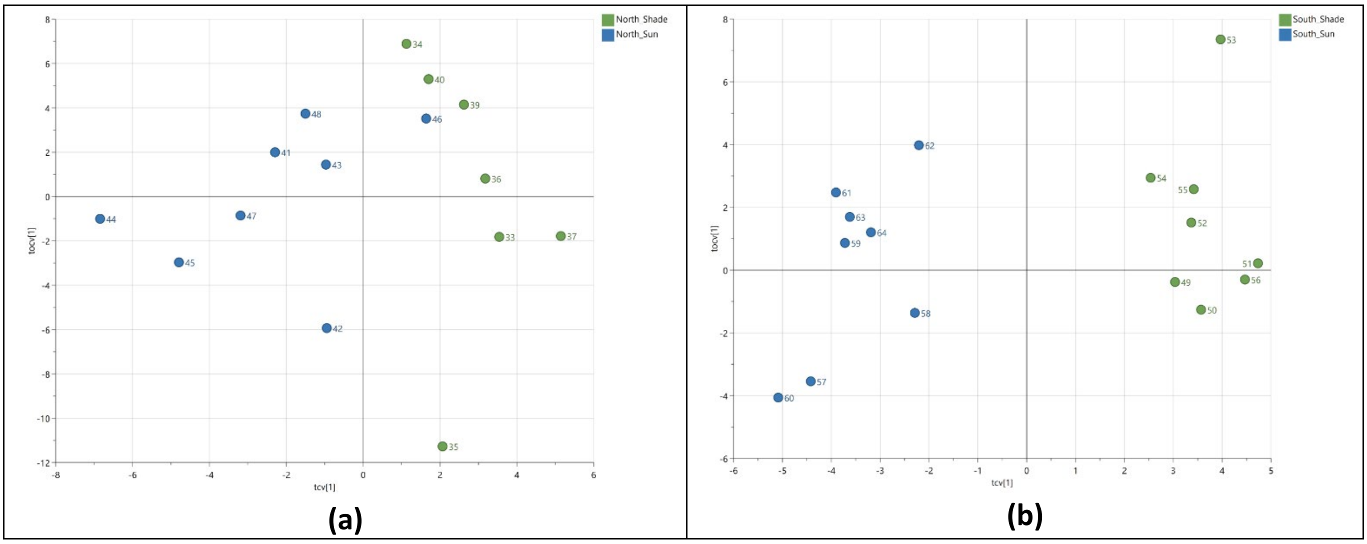

Figure S7 Scots pine samples, LCMS data: OPLS-DA shows differences between Shade and Sun conditions in both populations; targeted + untargeted metabolites, K = 781.

(a) Northern samples: R2Y = 0.97, Q2Y = 0.87
(b) Southern samples: R2Y = 0.98, Q2Y = 0.92

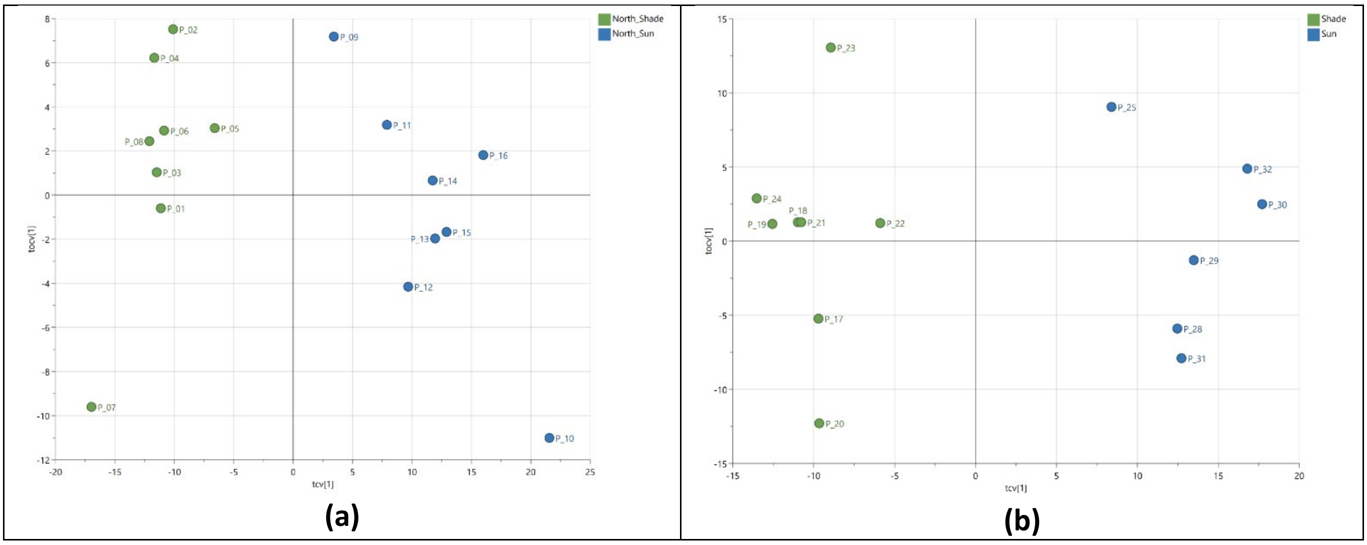

Figure S8 Scots pine samples, GCMS data: OPLS-DA shows differences between Shade and Sun conditions in both populations; K = 68.

(a) Northern samples: R2Y = 0.93 Q2Y = 0.87
(b) Southern samples: R2Y = 0.96, Q2Y = 0.93

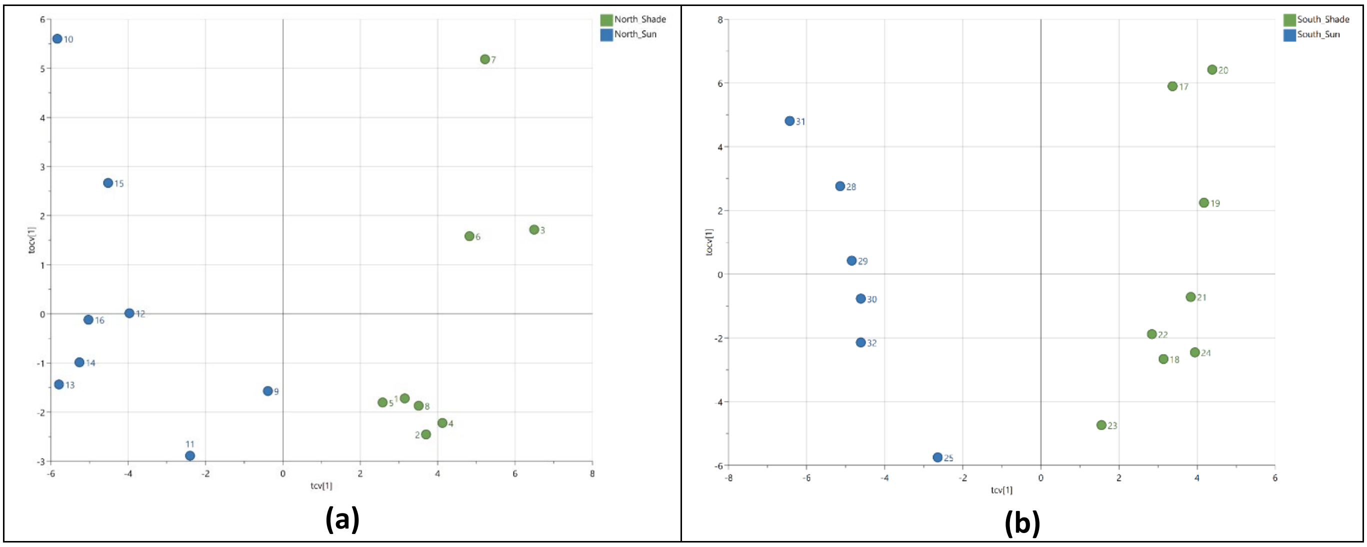

Figure S9 Norway Spruce LCMS data: OPLS-DA, South vs North models; targeted + untargeted metabolites, K = 799.

- Metabolite loadings for Northern ecotype plotted against Southern ecotype.
- Shows similarity between models and up/down regulation in Sun & Shade conditions.
- Regression line, R2 = 0.44 – There is a slight similarity in metabolic response due to light condition for the two ecotypes.

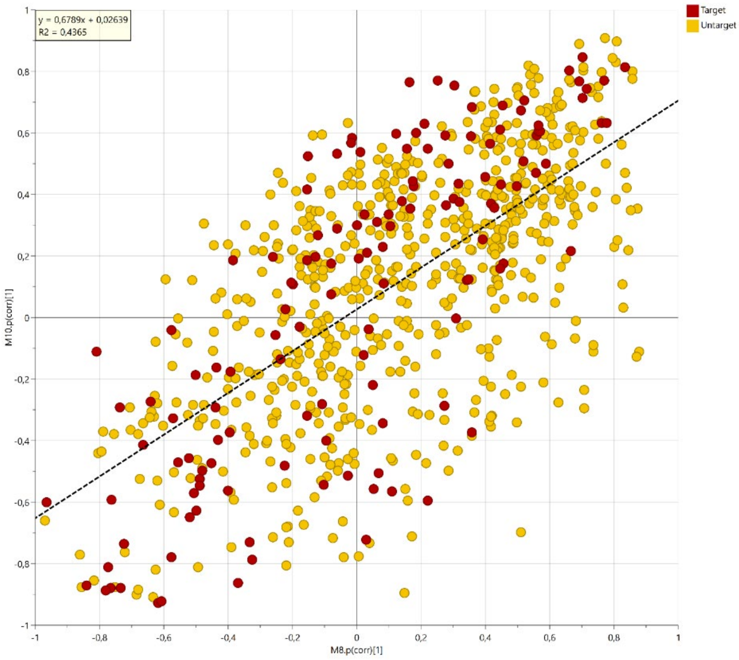

Figure S10 Norway Spruce GCMS data: OPLS-DA, South vs North models; K = 69.

- Metabolite loadings for Northern ecotypes plotted against Southern ecotypes.
- Shows similarity between models and up/down regulation in Sun & Shade conditions.
- Regression lines, R2 = 0.75 – High similarity in metabolic response for Sun and Shade conditions for the two ecotypes.

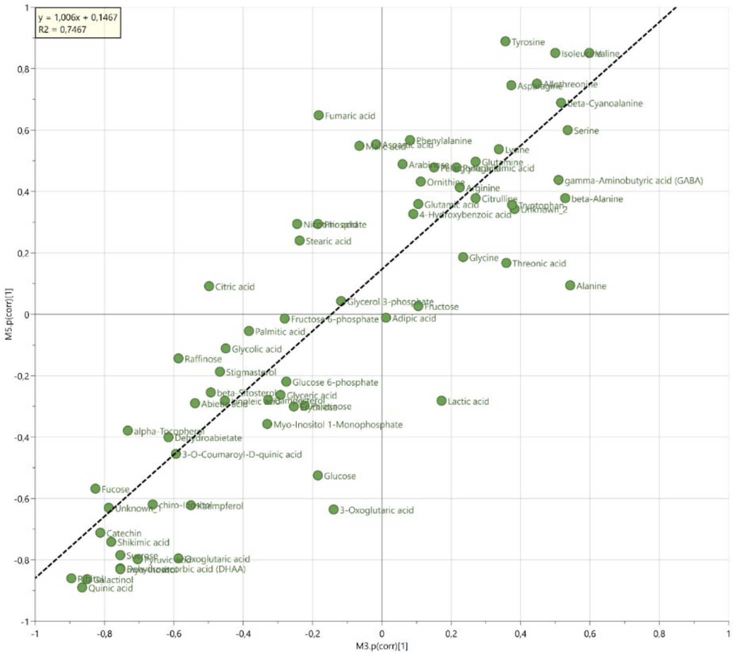

Figure S11 Scots pine LCMS data: OPLS-DA, South vs North models; targeted + untargeted metabolites, K = 781.

- Metabolites for Northern ecotype plotted against Southern ecotype.
- Shows similarity between ecotype models and up/down regulation in Sun & Shade conditions.
- Regression line, R2 = 0.73 – Pine exhibits a more similar metabolic response in both light conditions compared to spruce.

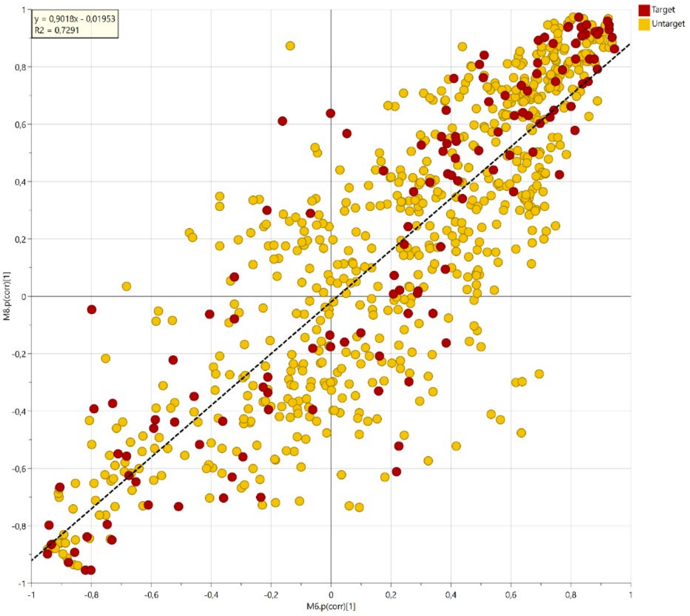

Figure S12 Scots pine GCMS data: OPLS-DA, South vs North models; K = 68.

- Metabolite loadings for Northern ecotypes plotted against Southern ecotypes.
- Shows similarity between models and up/down regulation in Sun & Shade conditions.

Regression lines, R2 = 0.86 – High similarity in metabolic response for Sun and Shade conditions for the two ecotypes.

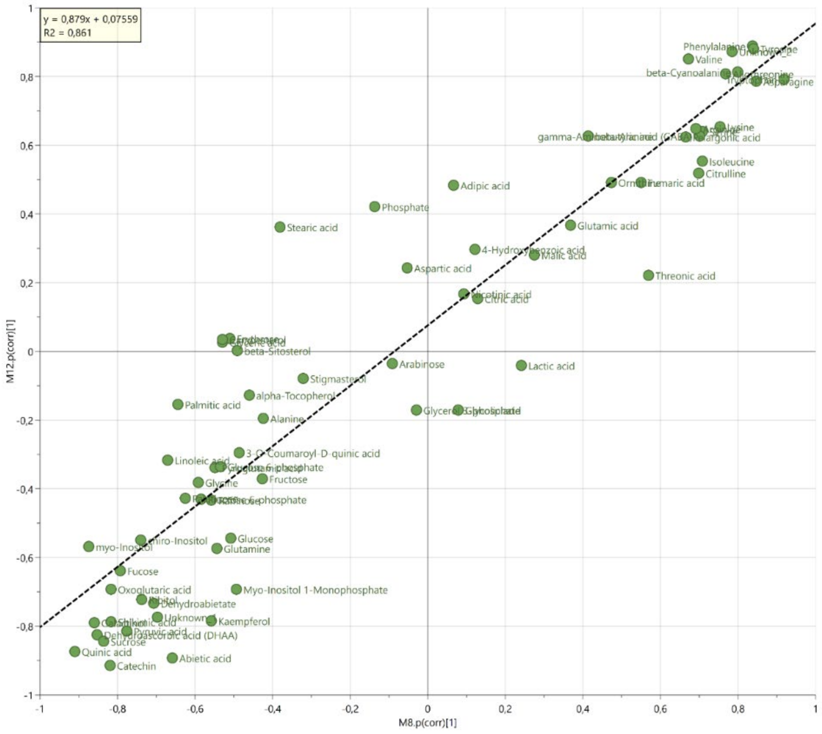

Figure S13 Overview of Pathway Analysis: Northern Norway spruce, Sun vs Shade
The x axis shows pathway impact scores that summarize normalized topology measures of those perturbed metabolites in each pathway. The y axis shows −log10(P) values of the enrichment analysis results. The sizes of the data points are correlated with their x values, and the colour gradients correspond to their y values.

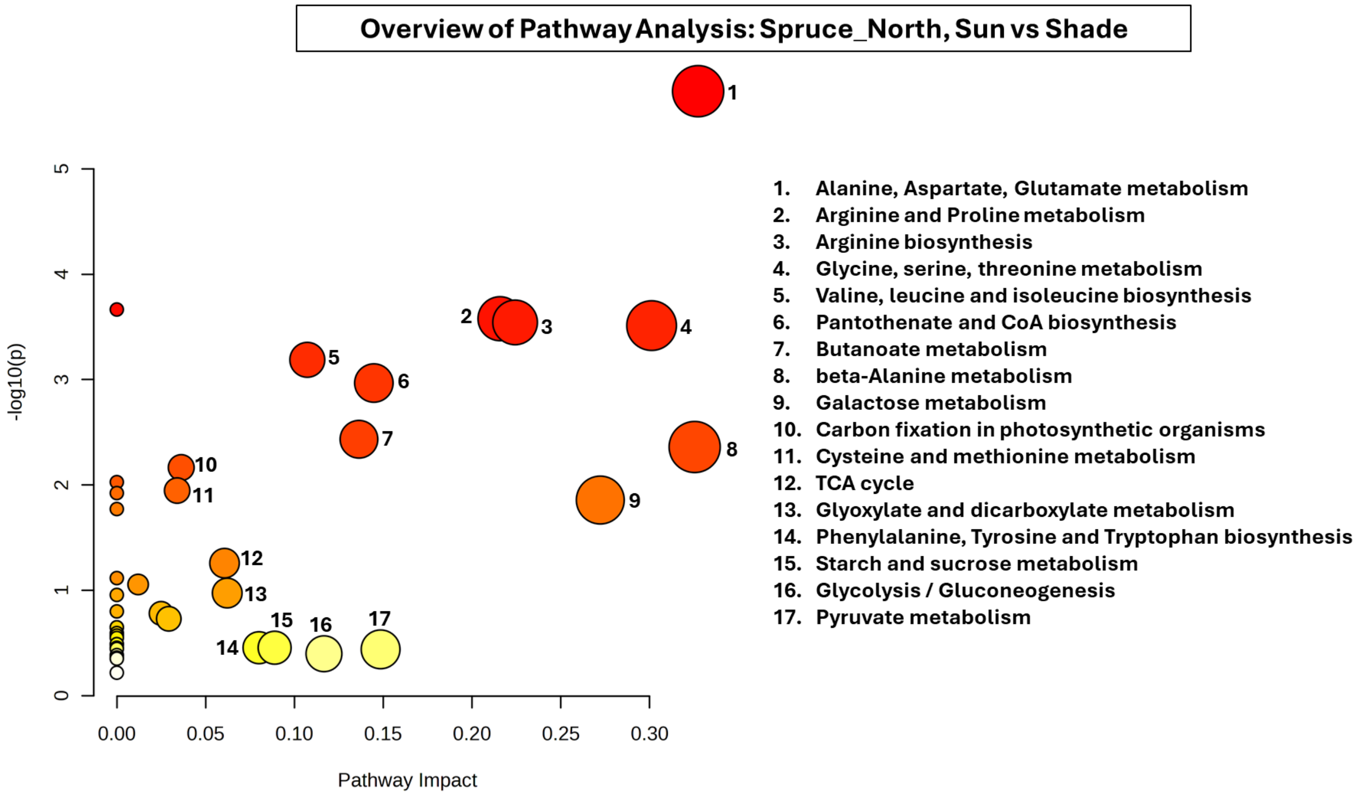

Figure S14 Overview of Pathway Analysis: Southern Norway spruce, Sun vs Shade

The x axis shows pathway impact scores that summarize normalized topology measures of those perturbed metabolites in each pathway. The y axis shows −log10(P) values of the enrichment analysis results. The sizes of the data points are correlated with their x values, and the colour gradients correspond to their y values.

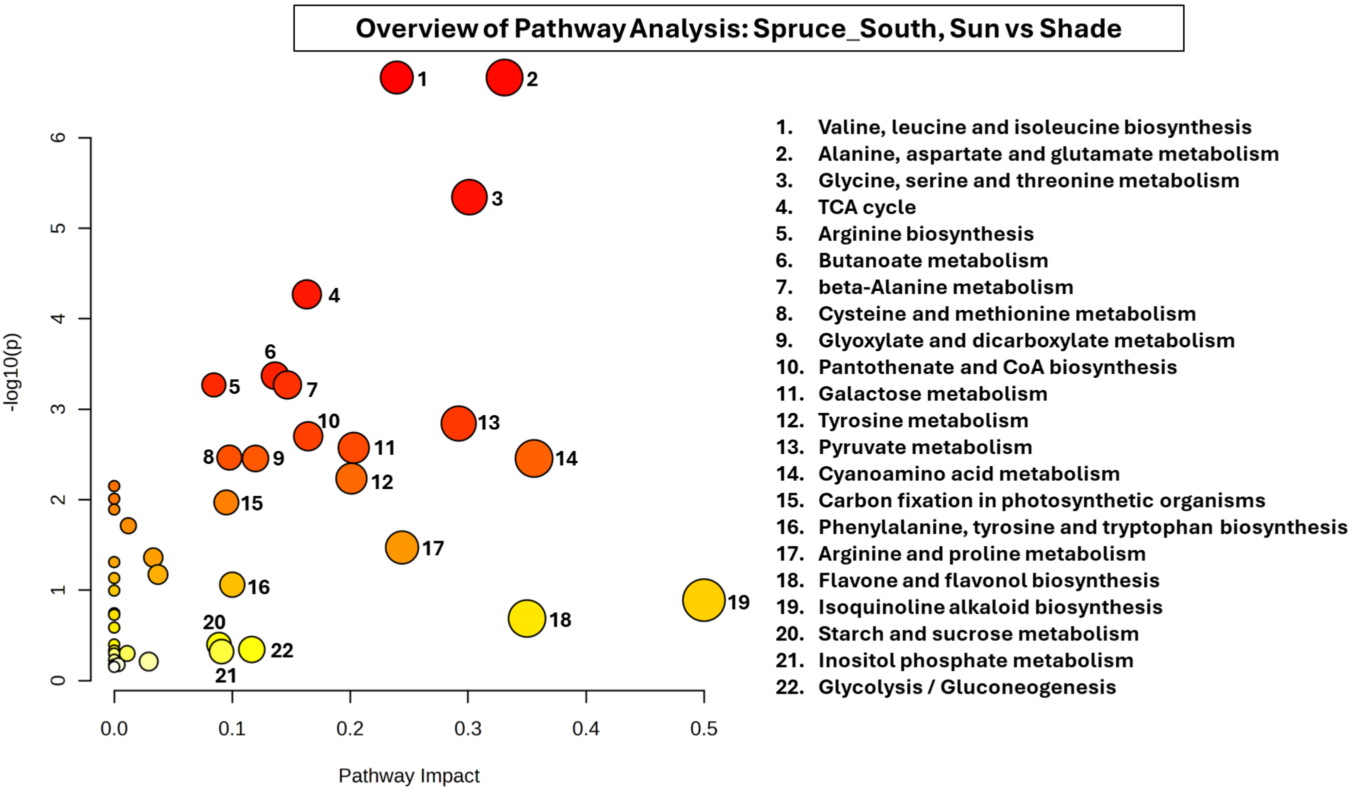

Figure S15 Overview of Pathway Analysis: Northern Scots pine, Sun vs Shade

The x axis shows pathway impact scores that summarize normalized topology measures of those perturbed metabolites in each pathway. The y axis shows −log10(P) values of the enrichment analysis results. The sizes of the data points are correlated with their x values, and the colour gradients correspond to their y values.

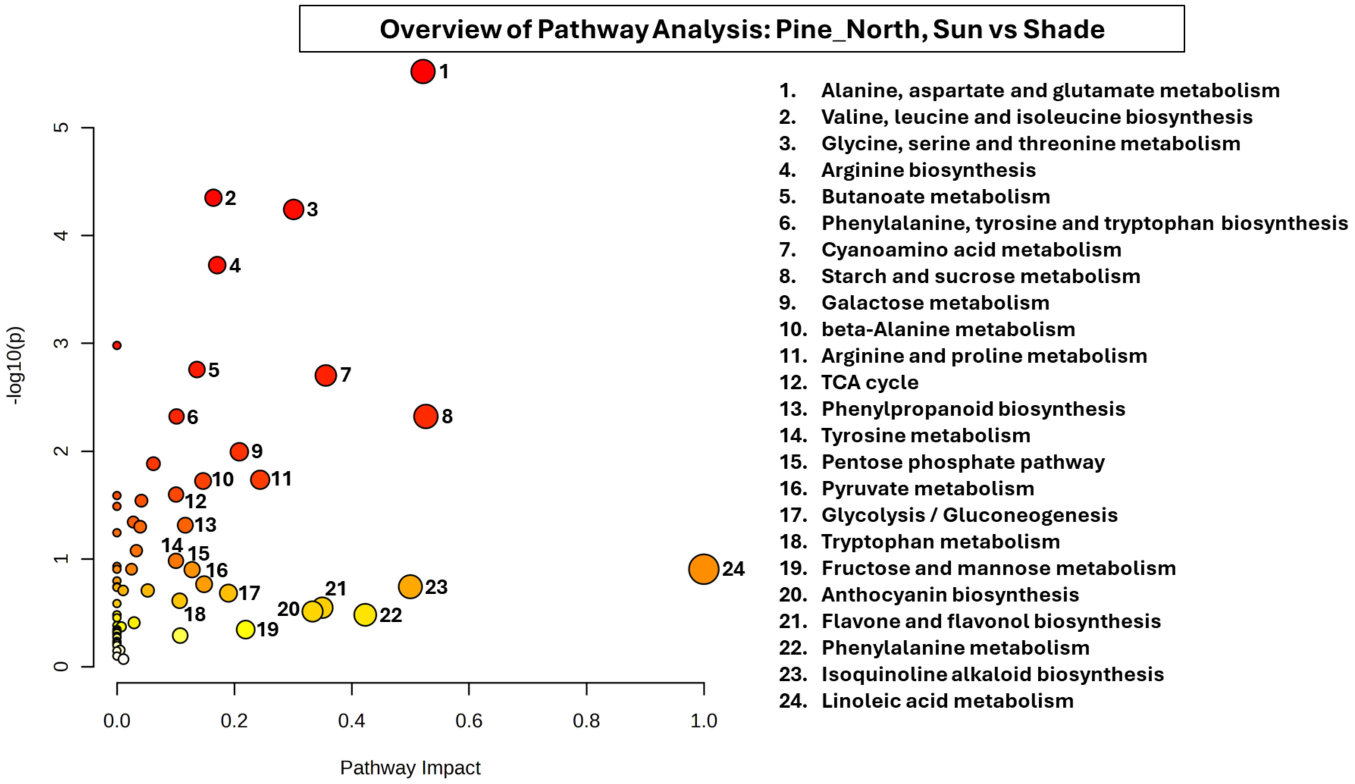

Figure S16 Overview of Pathway Analysis: Southern Scots pine, Sun vs Shade
The x axis shows pathway impact scores that summarize normalized topology measures of those perturbed metabolites in each pathway. The y axis shows −log10(P) values of the enrichment analysis results. The sizes of the data points are correlated with their x values, and the colour gradients correspond to their y values.

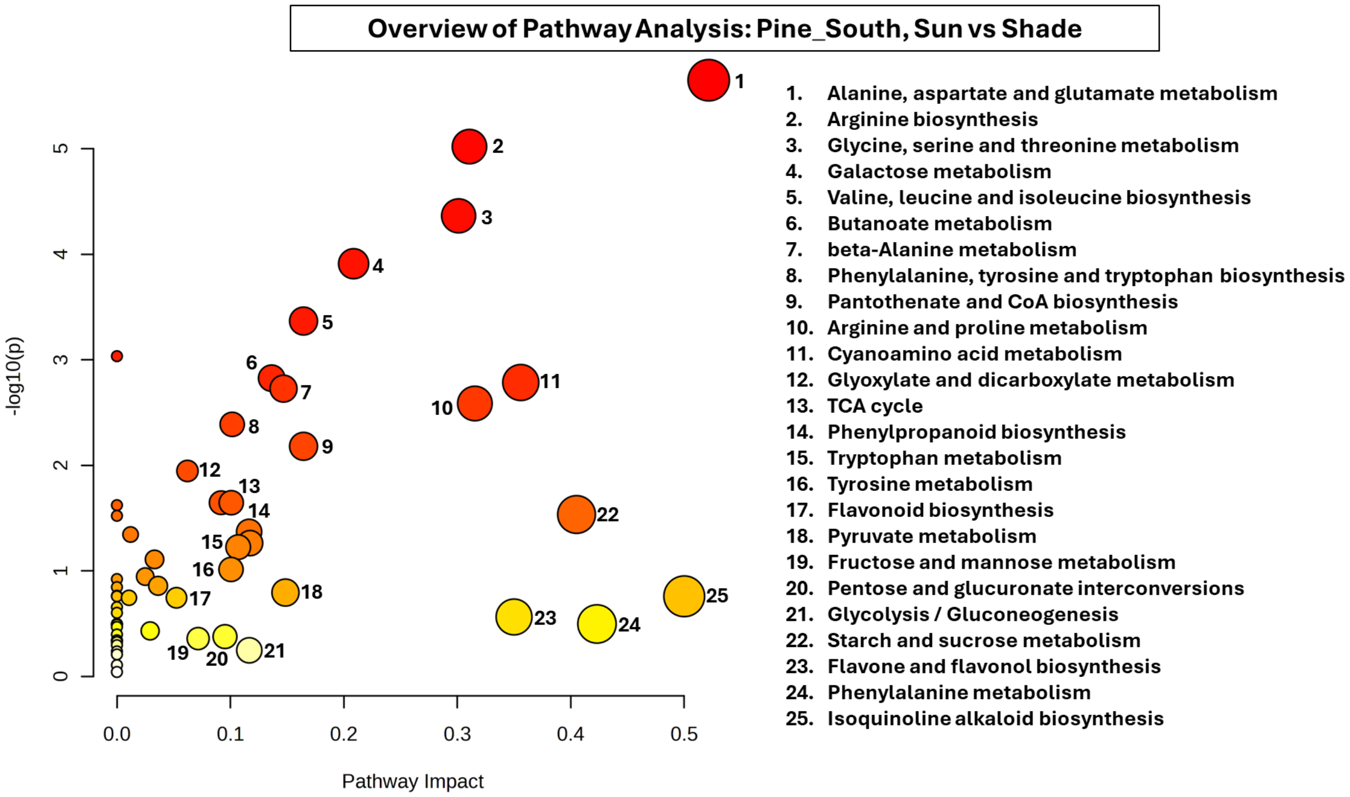

Figure S17 Multiple sequence alignment of PALs and PTALs from plant model systems and putative PALs and BALs (putative PTALs) from conifer species showing conserved amino acid residues (CLUSTAL O, 1.2.4). Colour codes – red: catalytically essential tyrosine; yellow: MIO region; green: asparagine and tyrosine stabilizing the MIO group; blue: arginine responsible for binding the carboxylic group of substrate. The conserved GTITASGDLVPLSYIA motif contains MIO region, marked in bold. Residues involved in substrate specificity for phenylalanine/tyrosine are marked with boxes (F/H, S/A, V/L, L/I, E/D).

```
PtaBAL3         ------------------------------------------------------------   0
PmeBAL4         ------------------------------------------------------------   0
PtaBAL2         -----------------------------------------------MPE-------AAS   6
PmeBAL3         ------------------------------------------------------------   0
PabBAL3         -------------------------------------------MVEVMAE-------AAP   10
PtaPAL4         ------------------------------------------------------------   0
PtaPAL3         ------------------------------------------------------------   0
PtaPAL1         ------------------------------------------------------------   0
PtaPAL2         ------------------------------------------------------------   0
PabPAL1         ------------------------------------------------------------   0
PmePAL1         ------------------------------------------------------------   0
OsPTAL          ------------------------------------------------------------   0
ZmPTAL          ------------------------------------------------------------   0
BdPTAL          ------------------------------------------------------------   0
BoPTAL          ------------------------------------------------------------   0
BoPAL           ------------------------------------------------------MECENG   6
OsPAL           ------------------------------------------------------MECENG   6
BdPAL           ------------------------------------------------------MECENG   6
ZmPAL           ------------------------------------------------------MESEAG   6
PcPAL           ------------------------------------------------------MENGNG   6
AtPAL           --------------------------------------------MEINGA---HKSNGGG   13
PtPAL           --------------------------------------------METITK----------   6
PabPAL2         --------------------------------------------MSSQGEEISGMENTNA   16
PsiPAL          --------------------------------------------MSSQGEEISRLENTNA   16
PmePAL2         ---------MLSLHYLAATSFLSAFSLRYCIAVPRPSASIHQIQEEIMSSTLGKENSVNG   51
PsyPAL          ------------------------------------------------------------   0
PsiBAL          -----------------------------------------MVAA---AAEM---TQTNE   13
PabBAL1         -----------------------------------------MVAA---AAEM---TQTNE   13
PmeBAL1         VSNKHSSFQLPCLALLCSAQFLDCL-VHFALQDHK------NMVA---GAEI---MQTNE   47
PsyBAL          ------------------------------------------MVA---AAGM---TQASE   12
PtaBAL1         ------------------------------------------MVA---AAEI---TQANE   12
PmeBAL2         ---------------------MVAG-VDMAVQANG------NGN-----------GNGNG   21
PabBAL2         ---------------------MVAG-IDLAVQANG------NQNGDLAVQVN---GNQNG   29


PtaBAL3         ------------MEFCQLNS--------------------NSCITARSRSCHVEDIILLI   28
PmeBAL4         ------------MECSQSKG--------------------KCC--KSAHSCHVKDIILLI   26
PtaBAL2         TVMI--ASIV--SSLAIKDDINI----Q-GCHEYDHDSLNWSRVADAMKGSHANEVKNMI   57
PmeBAL3         --VM--GSMV--SLLAIKNDINM----E-GVHAYDHDRLNWSRAADAMKGSHGKEVKNMI   49
PabBAL3         TVVM--GSM-------VKDDINM----E-SAHEYDHDGLNWSRAADAMKGSHAKEVRNMI   56
PtaPAL4         ---MASQEFTGLMNLCAGN-----------------DPLNWASVAESMKGSHFEEVKRMV   40
PtaPAL3         ---MAPQEFTGEVKFCAGND---------RV-SSLHDPLNWEAAAEPMKGSHFDEVKRMV   47
PtaPAL1         ---MAPQGITGEVKFCAGNG---------RV-SSLHDPLNWEAAAESMKGSHFDEVKRMV   47
PtaPAL2         ---MAPQGITGEVKFCAGNG---------RV-SSLHDPLNWEAAAESMKGSHFDEVKRMV   47
PabPAL1         ---MAPQEFTGEVKFCAGNG---------GTA-SLHDPLNWVAAAESMKGSHFEEVKRMV   47
PmePAL1         ---MAPQEFNGEVKFCAGNG---------GVGSNLHDPLNWAAAAESMKGSHFEEVKRMV   48
OsPTAL          ---------------MAGNG---------P--INKEDPLNWGAAAAEMAGSHLDEVKRMV   34
ZmPTAL          ---------------MAGNG---------A--IVESDPLNWGAAAAELAGSHLDEVKRMV   34
BdPTAL          ---------------MAGNG---------A--ISEKDPLNWGAAAAELTGSHLDEVKRMV   34
BoPTAL          ---------------MAGNG---------P--IVKDDPLNWGAAAAELTGSHFDEVKRMV   34
BoPAL           QVA--SNG----NGLCMATP--------------RADPLNWGKAAEELMGSHLEEVKRMV   46
OsPAL           RVS--ANGM---SGLCVAAP--------------RADPLNWGKATEEMTGSHLDEVKRMV   47
BdPAL           L----VGSL-NGEGLCMSAP---------P--RAAADPLNWAKTAEELAGSHLEEVKKMV   50
ZmPAL           LLV--RSSL-NGEGLCMPAP--------------RADPLNWGKAAEGLSGSHLDEVKRMV   49
PcPAL           --ATTNGHV-NGNGM---DF---------C--MKTEDPLYWGIAAEAMTGSHLDEVKKMV   49
AtPAL           VDAMLCGGD-IK-TK---NM---------V--INAEDPLNWGAAAEQMKGSHLDEVKRMV   57
PtPAL           -----NGYQ-NG-SS---ES---------L--CTQRDPLSWGVAAEAMKGSHLDEVKRMV   45
PabPAL2         L--M------RPMELCRPPILPLPESFGGKSPDHVIIPTHWKKAAEAMQCSHYEEVRKMI   68
PsiPAL          L--M------RPMELCRPPTLPLPESFGGKSPDHVIIPTHWKKAAEAMQCSHYEEVRKMI   68
PmePAL2         F--K------RPMELCRPPTLPLPGTLGNKTPDHVIIPTHWKKAAEALQCSHYEEVRKMI   103
PsyPAL          ------------------------------------------------------------   0
PsiBAL          VQQV------KSTGLC--------TGLSSSS----SDPLNWIRAAKAMEGSHFEEVKTMV   55
PabBAL1         VQQV------KSTGLC--------TGFSSSS----SDPLNWIRAAKAMEGSHFEEVKTMV   55
PmeBAL1         -VQV------KSTGLC--------TSFGSST----SDPLNWIRAAKAMEGSHFEEVKAMV   88
PsyBAL          -VQV------KSTGLC--------TDFGSSG----SDPLNWVRAAKAMEGSHFEEVKAMV   53
PtaBAL1         -VQV------KSTGLC--------TDFGSSG----SDPLNWVRAAKAMEGSHFEEVKAMV   53
PmeBAL2         FHHV------NAVDLC--------IQ---NG----PDPLNWGQAAKELQGSHFQEVKLMV   60
PabBAL2         FHQV------HSVDLC--------IQ---NG----PDPLNWGRAAKALQGSHFEEVKLMV   68
```

```
PtaBAL3        KTFNETNCINVDGSRITVAHVTALGRR---PQVKVTLDNHGGNCRERVERCS--------   77
PmeBAL4        NTFNEIQCINVDGSHITVAHVTALGRR---PQVKVALDDHGGHCRDNVERCS--------   75
PtaBAL2        EEFQGIKVVSLTGFDLNIAQVTALARR---PDVKVVLDELN--AKQRVDASS--------   104
PmeBAL3        EEFQGIKVVSLTGSNLSIAQVTALARR---PDVKVVLDKLN--CKDRVDASS--------   96
PabBAL3        EEFQGIKVVSLTGSSLSIAQVAALARR---PDVKVVLDELN--SKDGVDASS--------   103
PtaPAL4        EEFRA-PVVRLQGSGLTIAQVAAVARR--LGSVRVEFDT-G--ARARVEESS--------   86
PtaPAL3        EEFRA-PVVRLQGSGLTIAQVAAVARR--MGSVRVELET-G--AKARVDESS--------   93
PtaPAL1        EEFRA-PVVRLQGSGLTIAQVAAVARR--MGSVRVELET-G--AKARVDESS--------   93
PtaPAL2        EEFRA-PVVRLQGSGLTIAQVAAVARR--MGSVRVELET-G--AKARVDESS--------   93
PabPAL1        EEFRA-PVVKLQGSDLTIAQVAAVARR--LGSVRVELET-G--AKARVDESS--------   93
PmePAL1        EEFRS-PVVKLQGSGLTIAQVAAVARK--LGSVRVELET-C--AKARVDQSS--------   94
OsPTAL         AQFRE-PLVKIQGATLRVGQVAAVAQAKDAARVAVELDE-E--ARPRVKASS--------   82
ZmPTAL         AQARQ-PVVKIEGSTLRVGQVAAVASAKDASGVAVELDE-E--ARPRVKASS--------   82
BdPTAL         AQFRE-PVVKIEGASLRVGQVAAVAQAKDAAGVSVELDE-E--ARPRVKASS--------   82
BoPTAL         AQFRE-PVIKIEGASLRVGQVAAVAQAKDVSGVAVELDE-E--ARPRVKASS--------   82
BoPAL          AEYRQ-PVVKIEGASLRIAQVAAVA--AGAGEAKVQLDD-S--ARGRVKESS--------   92
OsPAL          AEYRQ-PLVKIEGASLRIAQVAAVA---AAGEARVELDE-S--ARERVKASS--------   92
BdPAL          AQFRM-PLVKIEGATLGIAQVAAVA--AGAGEARVELDE-S--ARGRVKESS--------   96
ZmPAL          AEFRD-PLVKIQGASLSVAQVAAVAVGAGGGEARVELDE-S--ARERVRASS--------   97
PcPAL          AEYRK-PVVKLGGETLTISQVAAISAR-DGSGVTVELSE-A--ARAGVKASS--------   96
AtPAL          AEFRK-PVVNLGGETLTIGQVAAISTI--GNSVKVELSE-T--ARAGVNASS--------   103
PtPAL          AEYRK-PVVNLAGQTLTIAQVASIAGH-DASNVKVELSE-S--ARPRVKASS--------   92
PabPAL2        GQFKATHKVVLRGTTLTVAEVTAVTRR---AEVKVELDEVS--AKQRVERSY--------   115
PsiPAL         GQFKATHKVVLRGTTLTVAEVTAVTRR---AEVKVELDEVS--AKQRVERSY--------   115
PmePAL2        AQFNATHKVVLRGTTLTVAEVTAVTRK---ATVKVELDEAS--AKERVQESY--------   150
PsyPAL         ------------------------------------------------------------   0
PsiBAL         DSYFESKEIFIEGKTLTIADVTAVARR---SQVKVKLDAAA--AKSRVEESS--------   102
PabBAL1        DSYFESKEIFIEGKTLTIADVTAVARR---SQVKVKLDAAA--AKSRVEESSNWVLTQMT   110
PmeBAL1        NSYLGSKEISIEGKSLTISDVAAVARR---SQVKVKLDAAA--AKSRVEESS--------   135
PsyBAL         DSYLGVKEIFIEGKSLTISDVAAVARR---SQVKVKLDAEA--AKSRVEESS--------   100
PtaBAL1        DSYFGAKEISIEGKSLTISDVAAVARR---SQVKVKLDAAA--AKSRVEESS--------   100
PmeBAL2        ESYFGSQEVSIEGKTLTIADVAAVARR---PEAKVRLDAVS--AKSRVDESS--------   107
PabBAL2        ESYFGSKEVSIEGKSLTIADVAAVARR---PEAKVRLDAVA--AKARVDESS--------   115

                                                           F/H

PtaBAL3        ------LWVQEKAKGGADIYGVTTGFGACSSKRTNQLSELQEALIRCLLAGVFTGP--SS   129
PmeBAL4        ------LWVREKAKEGADIYGVTTGFGACSSKRANQLSVLQEALIRCLLAGVFTGGPASS   129
PtaBAL2        ------EWVMNNINRGTDTYGITTGFGATSHRRTNQAVELQRELIRFLNAGVMGKRDS--   156
PmeBAL3        ------DWVMNNINKGTDTYGVTTGFGATSHRRTNQAVELQRELIRFLNAGVMGKGGS--   148
PabBAL3        ------DWVMNNINKGTDTYGVTTGFGATSHRRTNQAVELQKELIRFLNAGVMGKGDS--   155
PtaPAL4        ------NWVMDSIANGKAIYGVTTGFGASSHRRTSHGEALQKEMARFLNAGIFGGCGD--   138
PtaPAL3        ------NWVMDSMANGTDSYGVTTGFGATSHRRTRQGEALQKELIRFLNAGIFGACGD--   145
PtaPAL1        ------NWVMDSMANGTDSYGVTTGFGATSHRRTRQGEALQKELIRFLNAGIFGACGD--   145
PtaPAL2        ------NWVMDSMANGTDSYGVTTGFGATSHRRTRQGEALQKELIRFLNAGIFGACGD--   145
PabPAL1        ------NWVMDSMANGTDSYGVTTGFGATSHRRTRQGEALQKELIRFLNAGIFGGCGD--   145
PmePAL1        ------NWVMDSMANGTDSYGVTTGFGATSHRRTRQGEALQKELIRFLNAGIFGGCGD--   146
OsPTAL         ------EWILTCIAHGGDIYGVTTGFGGTSHRRTKDGPALQVELLRHLNAGIFGTGSD--   134
ZmPTAL         ------EWILDCIAHGGDIYGVTTGFGGTSHRRTKDGPALQVELLRHLNAGIFGTGSD--   134
BdPTAL         ------EWILSCLAAGGDIYGVTTGFGGTSHRRTKDGPALQVELLRHLNAGIFGTGSD--   134
BoPTAL         ------EWILNCLAHGGDIYGVTTGFGGTSHRRTKDGPALQVELLRHLNAGIFGTGTD--   134
BoPAL          ------DWVMNSMMNGTDSYGVTTGFGATSHRRTKEGGALQRELIRFLNAGAFGTGSD--   144
OsPAL          ------DWVMNSMMNGTDSYGVTTGFGATSHRRTKEGGALQRELIRFLNAGAFGTGTD--   144
BdPAL          ------DWVMNSMMNGTDSYGVTTGFGATSHRRTKEGGALQRELIRFLNAGAFGTGTD--   148
ZmPAL          ------DWVMGSMMNGTDSYGVTTGFGATSHRRTKEGGALQRELIRFLNAGAFGTGAD--   149
PcPAL          ------DWVMDSMNKGTDSYGVTTGFGATSHRRTKQGGALQKELIRFLNAGIFGNGSD--   148
AtPAL          ------DWVMESMNKGTDSYGVTTGFGATSHRRTKNGVALQKELIRFLNAGIFGSTKE--   155
PtPAL          ------DWVMDSMDKGTDSYGVTTGFGATSHRRTKQGGALQKELIRFLNAGIFGNGTE--   144
PabPAL2        ------QWVANNIAKGTDTYGVTTGFGATSHRRTDKAAELQKELIRFLNAGVVGKER---   166
PsiPAL         ------QWVANNIAKGTDTYGVTTGFGATSHRRTDKAAELQKELIRFLNAGVVGKER---   166
PmePAL2        ------QWVANNVARGTDTYGVTTGFGATSHRRTDKAEDLQKELIRFLNAGVVGKAR---   201
PsyPAL         ------QWVAKNVARGTDTYGVTTGFGATSHRRTDKAADLQKELIRFLNAGVVGREC---   51
PsiBAL         ------NWVLTQMTKGTDTYGVTTGFGATSHRRTNQGAELQKELIRFLNAGVLGKCQD--   154
PabBAL1        RVEESSNWVLTQMTKGTDTYGVTTGFGATSHRRTNQGAELQKELIRFLNAGVLGKCQD--   168
PmeBAL1        ------NWVLTQMTKGTDTYGVTTGFGATSHRRTNQGAELQKELIRFLNAGVLGKCQD--   187
PsyBAL         ------NWVLTQMTKGTDTYGVTTGFGATSHRRTNQGAELQKELIRFLNAGVLGKCPE--   152
PtaBAL1        ------NWVLTQMTKGTDTYGVTTGFGATSHRRTNQGAELQKELIRFLNAGVLGKCPE--   152
PmeBAL2        ------NWVLQNMLKGTDTYGVTTGFGATSHRRTNQGAELQKELIRFLNAGVLQ-AED--   158
PabBAL2        ------NWVLQNMLKGTDTYGVTTGFGATSHRRTSQGAELQKELIRFLNSGVLT--EG--   165
                      *:      *   **:*****. * :*: .   **  : * * :* .
```

```
                                                                        MIO
PtaBAL3     SPGELSPITARCAMFLRMSSFIYGCSGIRWEIMEALQQLINSHITPKCPLRGSVSASGDL   189
PmeBAL4     SPGELSPTTTRCAMFLRMNSFIYGCSGIRWEIMEALKKLINTHVTPKCPLRGSVSASGDL   189
PtaBAL2     --NCLSVSATRAAMLVRTNTLMQGFSGIRWEILEAMQKLLDSHITPKLPLRGTITASGDL   214
PmeBAL3     --NCLSVSATRAAMLVRTNTLMQGFSGIRWEILEALQKLLDSHITPKLPLRGTITASGDL   206
PabBAL3     --NCLSVSSTRAAMLVRTNTLMQGFSGIRWEILEAMQKLLDSHVTPKLPLRGTITASGDL   213
PtaPAL4     S-NTLPRDATRATMLVRTNTLLQGYSGIRWGILEAMTGLLNAGITPRLPLRGSITASGDL   197
PtaPAL3     S-NSLPRDTTRAAMLVRANTLLQGYSGIRWGILEAMSGLLNAGITPRLPLRGTITASGDL   204
PtaPAL1     S-NSLPRDTTRAAMLVRANTLLQGYSGIRWGILEAMSGLLNAGITPRLPLRGTITASGDL   204
PtaPAL2     S-NSLPRDTTRAAMLVRANTLLQGYSGIRWGILEAMSGLLNAGITPRLPLRGTITASGDL   204
PabPAL1     S-NSLPRDTTRAAMLVRANTLLQGYSGIRWGILEAMSGLLNAGITPRLPLRGTITASGDL   204
PmePAL1     S-NSLPRETTRAAMLVRANTLLQGYSGIRWEILEAMSGLLNAGITPRLPLRGTITASGDL   205
OsPTAL      G-HTLPSETVRAAMLVRINTLLQGYSGIRFEILEAITKLLNTGVTPCLPLRGTITASGDL   193
ZmPTAL      G-HTLPSEVTRAAMLVRINTLLQGYSGIRFEILEAITKLLNTGVSPCLPLRGTITASGDL   193
BdPTAL      G-HSLPAEVTRAAMLVRINTLLQGYSGIRFEILEAITKLLNTGVSPCLPLRGTITASGDL   193
BoPTAL      G-HTLPSEVTRAAMLVRINTLLQGYSGIRFEILEAITKLINTGVTPCLPLRGTITASGDL   193
BoPAL       G-HVLAAEATRAAMLVRINTLLQGYSGIRFEILEAIAKLLNANVTPCLPLRGTITASGDL   203
OsPAL       G-HVLPAEATRAAMLVRINTLLQGYSGIRFEILEAIAKLLNANVTPCLPLRGTITASGDL   203
BdPAL       G-HVLPAEATRAAMLVRINTLLQGYSGIRFEILEAITKLLNANVTPCLPLRGTITASGDL   207
ZmPAL       G-HVLPAEATRAAMLVRVNTLLQGYSGIRFEILEAIAKLLNANVTPCLPLRGTITASGDL   208
PcPAL       --NTLPHSATRAAMLVRINTLLQGYSGIRFEILEAITKFLNQNITPCLPLRGTITASGDL   206
AtPAL       TSHTLPHSATRAAMLVRINTLLQGFSGIRFEILEAITSFLNNNITPSLPLRGTITASGDL   215
PtPAL       TCHTLPHSATRAAMLVRINTLLQGYSGIRFEILEAITKLLNNNITPCLPLRGTITASGDL   204
PabPAL2     --LCLPAEYTKAAMLVRTNTLMQGYSGIRWEILDAMRKLMDCNITPKLPLRGTIE---AL   221
PsiPAL      --LCLPAEYTKAAMLVRTNTLMQGYSGIRWEILDAMRKLMDCNITPKLPLRGTITASGDL   224
PmePAL2     --LCLSAEYTKAAMLVRTNTLMQGYSGIRWEILDAMRKLMDCNIIPKLPLRGTITASGDL   259
PsyPAL      --LCLPAEYTKAAMLVRTNTLMQGYSGIRWEILDALRKLMDCNITPKLPLRGTITASGDL   109
PsiBAL      --NVLSEDTTRAAMLVRTNTLLQGYSGIRWDILETVEKLLNAGLTPKLPLRGTITASGDL   212
PabBAL1     --NVLSEDTTRAAMLVRTNTLLQGYSGIRWDILETVEKLLNAGLTPKLPLRGTITASGDL   226
PmeBAL1     --NILSEDTTRAAMLVRTNTLLQGYSGIRWDILETVEKLLNAGLTPKLPLRGTITASGDL   245
PsyBAL      --NVLSEDTTRAAMLVRTNTLLQGYSGIRWDILETVEKLLNAGLTPKLPLRGTITASGDL   210
PtaBAL1     --NVLSEDTTRAAMLVRTNTLLQGYSGIRWDILETVEKLLNAGLTPKLPLRGTITASGDL   210
PmeBAL2     --NVLPQATTRAAMLVRTNTLMQGYSGIRWEILETIQKLLNAGITPKLPLKGTITASGDL   216
PabBAL2     --NVLPQATTRAAMLVRTNTLMQGYSGIRWDILETIQKLLNAGITPKLPLKGTITASGDL   223
                *    .:.:*::* .::: * ****: *::::  :::  : *  **:*::     *

                                                                          A/S
PtaBAL3     IPLAYIAGLLIGNPQVKARIGAHGEEQEVPAPEALMKAGLQ--PFKLQAKEGLALVNGTS   247
PmeBAL4     IPLAYIAGLLIGNPHVKARIGHHDGHEELSAPEALVKTGLQ--PFKLQAKEGLALVNGTS   247
PtaBAL2     VPLSYIAGLLIARPNSVVVGI---DGNEMGAEEGLKLAGIDK-PFELNPKEGLALVNGTA   270
PmeBAL3     VPLSYIAGLLTARPNSVATRA---NGIEMGAEEALRMAGIDK-SFELNPKEGLALVNGTA   262
PabBAL3     VPLSYIAGLLTARSNSVAIGV---NGNEMGPKEALILAGIDK-PFELNPKEGLALVNGTA   269
PtaPAL4     VPLSYIAGLLIGRPNARAVMA---DGTEVGAAEALAAAGVGNGPFVLRPKEGVALVNATA   254
PtaPAL3     VPLSYIAGLLTGRSNARAVTA---NGTELGAAEALAAAGVENGPFELRPKEGLALVNGTA   261
PtaPAL1     VPLSYIAGLLTGRSNARAVTA---NGTELGAAEALAAAGVENGPFELRPKEGLALVNGTA   261
PtaPAL2     VPLSYIAGLLTGRSNARAVTA---NGTELGAAEALAAAGVENGPFELRPKEGLALVNGTA   261
PabPAL1     VPLSYIAGLLTGRPNARAVTA---DGRELGAAEALAAAGVENGPFELRPKEGLALVNGTA   261
PmePAL1     VPLSYIAGLLTGRPNARAVTA---DGKELGSAEALAAAGVENGPFELRPKEGLALVNGTA   262
OsPTAL      VPLSYIAGLITGRPNAQAISP---DGRKVDAAEAFKLAGIEGGFFTLNPKEGLAIVNGTS   250
ZmPTAL      VPLSYIAGLITGRPNAQAVTV---DGRKVDAAEAFKIAGIEGGFFKLNPKEGLAIVNGTS   250
BdPTAL      VPLSYIAGLITGRPNAQATTA---DGRKVDAAEAFKVAGIEGGFFTLNPKEGLAIVNGTS   250
BoPTAL      VPLSYIAGLITGRPNAQAVAP---DGRKVDAAEAFKIAGIEGGFFKLNPKEGLAIVNGTS   250
BoPAL       VPLSYIAGLVTGRENSVAVAP---DGRKVNAAEAFKIAGIQGGFFELQPKEGLAMVNGTA   260
OsPAL       VPLSYIAGLVTGRENAVAVAP---DGSKVNAAEAFKIAGIQGGFFELQPKEGLAMVNGTA   260
BdPAL       VPLSYIAGLITGRQNSVAVAP---DGSKVSAAEAFKIAGIEHGFFELQPKEGLAMVNGTA   264
ZmPAL       VPLSYIAGLITGRQNSVAVDP---DGRKVGAAEAFKIAGIEHGFFELQPKEGLAMVNGTA   265
PcPAL       VPLSYIAGLLTGRPNSKAVGP---TGVILSPEEAFKLAGVEGGFFELQPKEGLALVNGTA   263
AtPAL       VPLSYIAGLLTGRPNSKATGP---NGEALTAEEAFKLAGISSGFFDLQPKEGLALVNGTA   272
PtPAL       VPLSYIAGLLTGRPNSKATGP---NGEVLDAVEAFKAAGIDSGFFELQPKEGLALVNGTA   261
PabPAL2     VPLSYIAGLLTARPNSKALAP---DGHVLDAMDALRKAGIPE-PFKLQPKEGLALVNGTG   277
PsiPAL      VPLSYIAGLLTARPNSKALAP---DGQVLDAMDALRKASIPE-PFKLQPKEGLALVNGTG   280
PmePAL2     VPLSYIAGLLTARPNSRALTP---DGHELDAMAALRKASIPE-PFKLQPKEGLALVNGTA   315
PsyPAL      VPLSYIAGLLTARPNSKALSP---DGHLLDAMEALRKAGILE-PFKLQPKEGLALVNGTA   165
PsiBAL      VPLSYIAGLLTGRPNSRVRSR---DGTEMSGAEALKKVGLEK-PFELQPKEGLAIVNGTS   268
PabBAL1     VPLSYIAGLLTGRPNSRVRSR---DGTEMSGAEALQKIGLEK-PFELQPKEGLAIVNGTS   282
PmeBAL1     VPLSYIAGLLTGRPNSKVRSR---DGTEMSGAEALKKVGVEK-PFELAPKEGLAIVNGTS   301
PsyBAL      VPLSYIAGLLTGRPNSRVRSR---DGIEMSGAEALKKVGLEK-PFELQPKEGLAIVNGTS   266
PtaBAL1     VPLSYIAGLLTGRPNSRVRSR---DGIEMSGAEALKKVGLEK-PFELQPKEGLAIVNGTS   266
PmeBAL2     VPLSYIAGLLTGRPNSKARCR---DGKEIGALEALQQVGVEK-PFELQPKEGLAIVNGTS   272
PabBAL2     VPLSYIAGFLTGRPNSKGRCR---DGKELGALEALQQIGVEK-PFELQPKEGLAIVNGTS   279
            :**:****:: .. :             :    .:   .:    * *  ***:*:**.*.
```

```
PtaBAL3       FATALAATVMYDANVLLLLVEMLCGMFCEVIFGREEFAHPLIHEMKPHPGQRESAALLEW   307
PmeBAL4       FATALASTVMYDANVLLLLVETLCGMFCEVIFGREEFAHPLIHTMKPHVGQIQSAALLEW   307
PtaBAL2       VGSAVACTVCYDANVLAVFAEIGSAFFCEVMQGKPEFTDPLTHRLKHHPGQMEAGAVMEW   330
PmeBAL3       VGAAVACTVCYDANVLAVFAEIGSAFFCEVMQGKPEFTDPLTHRLKHHPGQIEAGAVMEW   322
PabBAL3       VGAAVACTVCYDANVLAVFAEIG---------------------------QMEAGAVMEW   302
PtaPAL4       VGSALAATVLFDANVVVLLSEVLSGLFCEVMQGDPGFTNHLIHRLKDHPGQIEAAAIMEH   314
PtaPAL3       VGSALAATVLFDANVVALLSEVLSAMFCEVMQGNPEFTDHLTHRLKHHPGQIEAAAIMEH   321
PtaPAL1       VGSALAATVLFDANVVALLSEVLSAMFCEVMQGNPEFTDHLTHRLKHHPGQIEAAAIMEH   321
PtaPAL2       VGSALAATVLFDANVVALLSEVLSAMFCEVMQGNPEFTDHLTHRLKHHPGQIEAAAIMEH   321
PabPAL1       VGSALAATVLFDANVVVLLSEVLSALFCEVMQGNPEFTDHLTHRLKHHPGQIEAAAIMEH   321
PmePAL1       VGSALAATVLFDANVVVLLSEVVSALFCEVMQGNPEFTDNLTHRLKHHPGQIEAAAIMEH   322
OsPTAL        VGSALAATVMFDANILAVLSEVLSAVFCEVMNGKPEYTDHLTHKLKHHPGSIDAAAIMEH   310
ZmPTAL        VGSALAATVMYDANVLAVLSEVLSAVFCEVMNGKPEYTDHLTHKLKHHPGSIEAAAIMEH   310
BdPTAL        VGSALAATVLFDCNVLAVLSEVLSAVFCEVMNGKPEFTDHLTHKLKHHPGSIEAAAIMEH   310
BoPTAL        VGSALAATVLYDCNVLAVLSEVLSAVFCEVMNGKPEYTDHLTHKLKHHPGSIEAAAIMEH   310
BoPAL         VGSGLASTVLFEANILAILAEVLSAVFCEVMNGKPEYTDHLTHKLKHHPGQIEAAAIMEH   320
OsPAL         VGSGLASTVLFEANILAILAEVLSAVFCEVMNGKPEYTDHLTHKLKHHPGQIEAAAIMEH   320
BdPAL         VGSGLASTVLFEANIQAILAEVLSAVFCEVMTGKPEFTDHLTHKLKHHPGQIEAAAIMEH   324
ZmPAL         VGSGLASTVLFEANVLAVLAEVISAVFCEVMTGKPEFTDHLTHKLKHHPGQIEAAAVMEH   325
PcPAL         VGSGMASMVLFEANILAVLAEVMSAIFAEVMQGKPEFTDHLTHKLKHHPGQIEAAAIMEH   323
AtPAL         VGSGMASMVLFETNVLSVLAEILSAVFAEVMSGKPEFTDHLTHRLKHHPGQIEAAAIMEH   332
PtPAL         VGSGLASMVLFETNVLAVLSELISAIFAEVMNGKPEFTDHLTHKLKHHPGQIEAAAIMEH   321
PabPAL2       VGSAVAASVCFDANVLVVLAEILSAFFCEVMQGKPEFVDPLTHQLKHHPGQIEAAAVMEY   337
PsiPAL        VGSAVAASVCFDANVLVVLAEILSAFFCEVMQGKPEFVDPLTHQLKHHPGQIEAAAVIEY   340
PmePAL2       VGSAVAASVCFDANVLGVLAEILSALFCEVMQGKPEFVDPLTHQLKHHPGQIEAAAVMEY   375
PsyPAL        VGSAVAASVCFDANVLGVLAEILSALFCEVMQGKPEFVDPLTHQLKHHPGQIEAAAVMEF   225
PsiBAL        VGAALASIVCFDANVLALLSEVISALFCEVMNGKPEFTDPLTHKLKHHPGQMEAAAIMEY   328
PabBAL1       VGAALASIVCFDANVLALLSEVISALFCEVMNGKPEFTDPLTHKLKHHPGQMEAAAIMEY   342
PmeBAL1       VGAALASIVCFDANVLALLSEVISAMFCEVMNGKPEFTDPLTHKLKHHPGQMEAAAIMEY   361
PsyBAL        VGAALASIVCFDANVLALLSEVISAMFCEVMNGKPEFTDPLTHKLKHHPGQMEAAAIMEY   326
PtaBAL1       VGAALASIVCFDANVLALLSEVISAMFCEVMNGKPEFTDPLTHKLKHHPGQMEAAAIMEY   326
PmeBAL2       VGAALASIVCFDANVICILAEVLSAMFCEVMLGKPEFTDPLTHKLKHHPAQMEAAAIMEY   332
PabBAL2       VGAALASIVCFDANVICILAEVLSAMFCEVMLGKPEFTDPLTHRLKHHPAQMEAAAIMEY   339
              ..:.:*. * :: *:  :: *                                  . ::.*::*

PtaBAL3       LLRDSPFQELS-REYYSINSLKKPKQDRYALRSSPQWLAPLVQTIRE-------------   353
PmeBAL4       LLRDSPFQELS-REYYSINSLKKPKQDRYALRSSPQWLAPIVQTIRE-------------   353
PtaBAL2       LLDGSSYSF---LKLAETDPLKKPKQDRYALRTSPQWLGPQIEVIRM-------------   374
PmeBAL3       ILDGSPYVN---PKLAETDPLKKPKQDRYALRTSPQWLGPQIEVIRM-------------   366
PabBAL3       LLDGSSYSY---LKLVETDPLKKPKQDRYALRTSPQWLGPQIEVIRM-------------   346
PtaPAL4       LLDGSSYMKAAAAKNKEADPMSKPKKDRYALYTSPQWLGPQVEVIRA-------------   361
PtaPAL3       LLEGSSYMKAAAAKHHEADALSKPKQDRYALRTAPQWLGPQIEVIRA-------------   368
PtaPAL1       LLEGSSYMKAAAAKHHEADALSKPKQDRYALRTAPQWLGPQIEVIRA-------------   368
PtaPAL2       LLEGSSYMKAAAAKHHEADALSKPKQDRYALRTAPQWLGPQIEVIRA-------------   368
PabPAL1       LLDGSSYMKAAAAKHQEADALSKPKQDRYALRTAPQWLGPQVEVLRA-------------   368
PmePAL1       LLDGSSYMKAMAAKHQEADALSKPKQDRYALRTSPQWLGPQVEVIRA-------------   369
OsPTAL        ILAGSSFMSHA-KKVNEMDPLLKPKQDRYALRTSPQWLGPQIQVIRA-------------   356
ZmPTAL        ILDGSSFMKQA-KKLNELDPLLKPKQDRYALRTSPQWLGPQIEVIRA-------------   356
BdPTAL        ILAGSSFMSHA-KKVNEIDPQLKPKQDRYALRTSPQWLGPQIEVIRS-------------   356
BoPTAL        ILAGSSFMSHA-KKVNEMDPLLKPKQDRYALRTSPQWLGPQIEVIRA-------------   356
BoPAL         ILEGSSYMKLA-KKLGELDPLMKPKQDRYALRTSPQWLGPQIEVIRA-------------   366
OsPAL         ILEGSSYMKHA-KKLGELDPLMKPKQDRYALRTSPQWLGPQIEVIRA-------------   366
BdPAL         ILEGSSYMKEA-KKQGELDPLMKPKQDRYALRTSPQWLGPQIEVIRF-------------   370
ZmPAL         ILEGSSYMKLA-KRLGELDPLMKPKQDRYALRTSPQWLGPQIEVIRF-------------   371
PcPAL         ILDGSAYVKAA-QKLHEMDPLQKPKQDRYALRTSPQWLGPQIEVIRS-------------   369
AtPAL         ILDGSSYMKLA-QKLHEMDPLQKPKQDRYALRTSPQWLGPQIEVIRY-------------   378
PtPAL         ILDGSAYMKAA-KKLHEMDPLQKPKQDRYALRTSPQWLGPQIEVIRF-------------   367
PabPAL2       LLDGSDYVKEA-ARLHERDPLSKPKQDRYALRTSPQWLGPQIEVIRA-------------   383
PsiPAL        LLDGSDYVKEA-ARLHERDPLSKPKQDRYALRTSPQWLGPQIEVIRA-------------   386
PmePAL2       LLDGSDYVKEA-ARLHESDPLSKPKQDRYALRTSPQWLGPQIEVIRA-------------   421
PsyPAL        LLDGSDYVKEA-ARLHERDPLSKPKQDRYALRTSPQWLGPPIEVIRA-------------   271
PsiBAL        VLDGSSYMKHA-AKLHEMNPLQKPKQDRYALRTSPQWLGPQAEVIRS-------------   374
PabBAL1       VLDGSSYMKHA-AKLHEMNPLQKPKQDRYALRTSPQWLGPQVEVIRSATHMIESQVEVIR   401
PmeBAL1       VLDGSSYMKHA-AKLHEMNPLQKPKQDRYALRTSPQWLGPQVEVIRS-------------   407
PsyBAL        VLDGSSYMKHA-AKLHEMNPLQKPKQDRYALRTSPQWLGPQVEIIRS-------------   372
PtaBAL1       VLDGSSYMKHA-AKLHEMNPLQKPKQDRYALRTSPQWLGPQVEIIRS-------------   372
PmeBAL2       VLDGSSYMKNA-AKKHEMNPLQKPKQDRYALRTSPQWLGPQIEVIRA-------------   378
PabBAL2       ---------------KHEMNPLQKPKQDRYALRTSPQWLGPQIEVIRA-------------   372
                                . :   ***:***** ::****.*  : :*
```

```
                                 L/V         I/L

PtaBAL3     -ATATIETEINSANDNPIIDPVNGKALHGANFQGSAVGFYMDYVRVALAGLGKLIFAQFT  412
PmeBAL4     -ATATIETEINSANDNPIIDHVNGKALHGANFQGSAVGFYMDYVRVAVAGLGKLLFAQFT  412
PtaBAL2     -STHAIAREINSVNDNPLIDVVRDRALHGGNFQGTPIGVYMDNIRLALAAIAKLIFAQFS  433
PmeBAL3     -ATHAIAREINSVNDNPLIDVSRDRALHGGNFQGTPIGVSMDNIRLALAAIAKLIFAQFS  425
PabBAL3     -ATHAIAREINSVNDNPLIDVVRDRALHGGNFQGTSIGVSMDNIRLALAAIAKLIFAQFS  405
PtaPAL4     -STHMVQREINSVNDNPIIDAAGNKALNGGNFQGTPVGVAMDNVRLALAAVGKLIFAQMS  420
PtaPAL3     -STHMVQREINSVNDNPLIDAARNKALHGGNFQGTPVGVAMDNARLALAAVGKLMFAQML  427
PtaPAL1     -STHMVQREINSVNDNPLIDAARNKALHGGNFQGTPVGVAMDNARLALAAVGKLMFAQMS  427
PtaPAL2     -STHMVQREINSVNDNPLIDAARNKALHGGNFQGTPVGVAMDNARLALAAVGKLMFAQMS  427
PabPAL1     -STHMVQREINSVNDNPLIDAARNKALHGGNFQGTPVGVAMDNARLALAAVGKLMFAQMS  427
PmePAL1     -STHMVQREINSVNDNPLIDAARNKALHGGNFQGTPVGVAMDNARLALAAVGKLLFAQMS  428
OsPTAL      -ATKSIEREVNSVNDNPVIDVHRGKALHGGNFQGTPIGVSMDNARLAIANIGKLMFAQFS  415
ZmPTAL      -ATKSIEREVNSVNDNPVIDVHRGKALHGGNFQGTPIGVSMDNARLAIANIGKLMFAQFS  415
BdPTAL      -ATKSIEREVNSVNDNPVIDVHRGKALHGGNFQGTPIGVSMDNTRLAIANIGKLMFAQFS  415
BoPTAL      -ATKSIEREVNSVNDNPVIDVHRGKALHGGNFQGTPIGVSMDNTRLAIANIGKLMFAQFS  415
BoPAL       -ATKSIEREINSVNDNPLIDVSRGKALHGGNFQGTPIGVSMDNTRLAIAAVGKLMFAQFS  425
OsPAL       -ATKSIEREINSVNDNPLIDVSRGKALHGGNFQGTPIGVSMDNTRLAIAAIGKLMFAQFS  425
BdPAL       -ATKSIEREINSVNDNPLIDVSRGKAIHGGNFQGTPIGVSMDNTRLAIAAIGKLMFAQFS  429
ZmPAL       -ATKSIEREINSVNDNPLIDVSRGKALHGGNFQGTPIGVSMDNTRLALAAIGKLMFAQFS  430
PcPAL       -STKMIEREINSVNDNPLIDVSRNKAIHGGNFQGTPIGVSMDNTRLAIAAIGKLMFAQFS  428
AtPAL       -ATKSIEREINSVNDNPLIDVSRNKAIHGGNFQGTPIGVSMDNTRLAIAAIGKLMFAQFS  437
PtPAL       -STKSIEREINSVNDNPLIDVSRNKALHGGNFQGTPIGVSMDNVRLAIASIGKLLFAQFS  426
PabPAL2     -ATHSIEREINSVNDNPLIDLSRDMALHGGNFQGTPIGVSMDNMRISLAAIGKLIFAQFS  442
PsiPAL      -ATHSIEREINSVNDNPLIDVSRDMALHGGNFQGTPIGVSMDNMRISLAAIGKLIFAQFS  445
PmePAL2     -ATHSIEREINSVNDNPLIDVSRDMALHGGNFQGTPIGVSMDNMRIALAAIGKLLFAQFS  480
PsyPAL      -ATHSIEREINSVNDNPLIDVSRDMALHGGNFQGTPIGVSMDNMRISLAAVGKLLFAQFS  330
PsiBAL      -ATHMIEREINSVNDNPVIDVARDLALHGGNFQGTPIGVSMDNLRLAISAIGRLMFAQFS  433
PabBAL1     SATHMIEREINSVNDNPVIDVARDLALHGGNFQGTPIGVSMDNLRLAISAIGRLMFAQFS  461
PmeBAL1     -ATHMIEREINSVNDNPVIDVARDKALHGGNFQGTPIGVSMDNLRLAISAIGKLMFAQFS  466
PsyBAL      -ATHMIEREINSVNDNPVIDVARDKALHGGNFQGTPIGVSMDNLRLSISAIGKLMFAQFS  431
PtaBAL1     -ATHMIEREINSVNDNPVIDVARDKALHGGNFQGTPIGVSMDNLRLSISAIGKLMFAQFS  431
PmeBAL2     -ATHMIQREINSVNDNPVIDVARDKALHGGNFQGTPIGVSMDNLRLALSAIGKLMFAQFS  437
PabBAL2     -ATHMIQREINSVNDNPVIDVARDKALHGGNFQGTPIGVSMDNLRLALAAIGKLMFAQFS  431
             :*  :  *:**.****:**    .  *:*.****: :*. **  *:::: :.:*:***:

                D/E

PtaBAL3     ELMIEFYSNGLPGNLSLGPDLSLDYGFKGIDIAMAAYSSELQFLANPVTTHVHSAEQHNQ  472
PmeBAL4     ELMIEFYSNGLPSNLALGPDLSLDYGFKGVDIAMAAYSSELQFLANPVTTHVHSAEQHNQ  472
PtaBAL2     ELVNEYYNNGLPSNLSGGADPSLDYGLKGAEIAMASYTSEILFLANPVTNHVQSAEQHNQ  493
PmeBAL3     ELVNEYYNNGLPSNLSGGADPSLDYGLKGAEIAMASYTSEILFLANPVTSHVQSAEQHNQ  485
PabBAL3     ELVNEYYNNGLPSNLSGGADPSLDYGLKGAEIAMASYTSEIMFLANPVTNHVQSAEQHNQ  465
PtaPAL4     ELVNDFYNKGLPSNLSGGPDPSLDYGFKGAEVAMASYTSELQFLANPVTTHVQSAEQHNQ  480
PtaPAL3     ELVNDFYNNGLPSNLSGGPDPSLDYGFKGAEIAMAAYTSELQFLANPVTTHVQSAEQHNQ  487
PtaPAL1     ELVNDFYNNGLPSNLSGGPDPSLDYGFKGAEIAMAAYTSELQFLANPVTTHVQSAEQHNQ  487
PtaPAL2     ELVNDFYNNGLPSNLSGGPDPSLDYGFKGAEIAMAAYTSELQFLANPVTTHVQSAEQHNQ  487
PabPAL1     ELVNDFYNNGLPSNLSGGPDPSLDYGFKGAEIAMAAYTSELQFLANPVTTHVQSAEQHNQ  487
PmePAL1     ELVNDFYNNGLPSNLSGGPDPSLDYGFKGAEIAMAAYTSELQFLANPVTTHVQSAEQHNQ  488
OsPTAL      ELVNEFYNNGLTSNLAGSRNPSLDYGFKGTEIAMASYSSELQYLANPITNHVQSAEQHNQ  475
ZmPTAL      ELVNEFYNNGLTSNLAGSRNPSLDYGFKGTEIAMASYCSELQYLGNPITNHVQSAEQHNQ  475
BdPTAL      ELVNEFYNNGLTSNLAGSRNPSLDYGFKGTEIAMASYCSELQYLANPVTNHVQSAEQHNQ  475
BoPTAL      ELVNEFYNNGLTSNLAGSRNPSLDYGFKGTEIAMASYCSELQYLANPITNHVQSAEQHNQ  475
BoPAL       ELVNDFYNNGLPSNLSGGRNPSLDYGFKGAEIAMASYCSELQFLGNPVTNHVQSAEQHNQ  485
OsPAL       ELVNDFYNNGLPSNLSGGRNPSLDYGFKGAEIAMASYCSELQFLGNPVTNHVQSAEQHNQ  485
BdPAL       ELVNDFYNNGLPSNLSGGRNPSLDYGFKGAEIAMASYCSELQFLGNPVTNHVQSAEQHNQ  489
ZmPAL       ELVNDYYNNGLPSNLSGGRNPSLDYGFKGAEIAMASYCSELQFLGNPVTNHVQSAEQHNQ  490
PcPAL       ELVNDFYNNGLPSNLSGGRNPSLDYGFKGAEIAMASYCSELQFLANPVTNHVQSAEQHNQ  488
AtPAL       ELVNDFYNNGLPSNLTASRNPSLDYGFKGAEIAMASYCSELQYLANPVTSHVQSAEQHNQ  497
PtPAL       ELVNDFYNNGLPSNLTASRNPSLDYGFKGAEIAMASYCSELQYLANPVTSHVQSAEQHNQ  486
PabPAL2     ELVCDYYNNGLPSNLSGGPNPSLDYGFKGAEIAMAAYTSELQYLANPVTTHVQSTDQHNQ  502
PsiPAL      ELVCDYYNNGLPSNLSGGPNPSLDYGFKGAEIAMAAYTSELQYLANPVTTHVQSAEQHNQ  505
PmePAL2     ELVCDYYNNGLPSNLSGGPNPSLDYGFKGAEIAMAAYTSELQYLANPVTTHVQSAEQHNQ  540
PsyPAL      ELVCDYYNNGLPSNLSGGPNPSLDYGFKGAEIAMAAYTSELQYLANPVTTHVQSAEQHNQ  390
PsiBAL      ELVNDYYNGGLPSNLSGGPNPSLDYGLKGAEIAMASYTSELLYLANPVTSHVQSAEQHNQ  493
PabBAL1     ELVNDYYNGGLPSNLSGGPNPSLDYGLKGAEIAMASYTSELLYLANPVTSHVQSAEQHNQ  521
PmeBAL1     ELVNDYYNGGLPSNLSGGPNPSLDYGLKGAEIAMASYTSELLYLANPVTSHVQSAEQHNQ  526
PsyBAL      ELVNDYYNGGLPSNLSGGPNPSLDYGLKGAEIAMASYTSELLYLANPVTSHVQSAEQHNQ  491
PtaBAL1     ELVNDYYNGGLPSNLSGGPNPSLDYGLKGAEIAMASYTSELLYLANPVTSHVQSAEQHNQ  491
PmeBAL2     ELVNDYYNGGLPSNLSGGPNPSLDYGFKGAEIAMAAYTSELLYLANPVTTHVQSAEQHNQ  497
PabBAL2     ELVNDYYNGGLPSNLSGGPNPSLDYGFKGAEIAMAAYTSELQYLASPVTTHVQSAEQHNQ  491
            **: .:*. ** .**: . : *****:** ::***:* **: :*..*:*.**:*::****
```

```
PtaBAL3     DINSLALISARKTEEALDILKLMLASHLYALCQAIDLRQLEQILLNIVLGIISSVSDECH  532
PmeBAL4     DINSLALISARKTEEALDILKLMVASHLSALCQAIDLRQLEQMLVKTVLGVISSVSDACH  532
PtaBAL2     DVNSLGLISARKTAEAVDILKLMVSTYLIALCQAVDLRHLEENFHGAVKQIVCQAVRRIL  553
PmeBAL3     DVNSLGLISARKTAEAVEILELMVSTYLIALCQAVDLRHLEENFHGGVKQIVCQAAGRVL  545
PabBAL3     DVNSLGLMSARKTAEAVDILKLMVSTYLIALCQAVDLRHLEENFHSGVKQIVCQAVRTIL  525
PtaPAL4     DVNSLGLISARMTAQAVEILKLMTSTYLVALCQAIDLRHLEENLHAAVRQAVGEACKKTL  540
PtaPAL3     DVNSLGLISARMTAQAVEILKLMTSTYLVALCQAIDLRHLEENLQGTVRQAVGQTFKNTL  547
PtaPAL1     DVNSLGLISARMTAQAVEILKLMTSTYLVALCQAIDLRHLEENLQGAVRQAVGQTFKKTL  547
PtaPAL2     DVNSLGLISARMTAQAVEILKLMTSTYLVALCQAIDLRHLEENLQGTVRQAVGQTFKKTL  547
PabPAL1     DVNSLGLISARMTAQAVEILKLMTSTYLVALCQAIDLRHLEENLQTAVRQAVAQACKKTL  547
PmePAL1     DVNSLGLISARMTAQAVEILKLMSSTYLVALCQAIDLRHLEENLQTAVRQAVGQACKKTL  548
OsPTAL      DVNSLGLVSARKTLEAVDILKLMTSTYIVALCQAVDLRHLEENIKSSVKNCVTQVAKKVL  535
ZmPTAL      DVNSLGLVSARKTAEAIDILKLMSSTYIVALCQAVDLRHLEENIKASVKNTVTQVAKKVL  535
BdPTAL      DVNSLGLVSARKTAEAVDILKLMSSTYMVALCQAVDLRHLEENIKASVKNCVTQVSKKVL  535
BoPTAL      DVNSLGLVSARKTAEAVDILKLMSSTYMVALCQAVDLRHLEENIKSSVKNCVTQVAKKVL  535
BoPAL       DVNSLGLISSRKTAEAIDILKIMSSTFLVALCQAIDLRHIEENVKSAVKSCVMTVAKKTL  545
OsPAL       DVNSLGLISSRKTAEAIDILKLMSSTFLIALCQAVDLRHIEENVKSAVKSCVMTVAKKTL  545
BdPAL       DVNSLGLISSRKTAEAIDILKIMSSTFLIALCQAIDLRHLEENMKTAVRNCVMQVAKKTL  549
ZmPAL       DVNSLGLISSRKTAEAIDILKLMSSTFLIALCQAIDLRHLEENVKAAVKNCVTQVAKKSL  550
PcPAL       DVNSLGLISSRKTSEAVEILKLMSTTFLVGLCQAIDLRHLEENLKSTVKNTVSSVAKRVL  548
AtPAL       DVNSLGLISSRKTSEAVDILKLMSTTFLVAICQAVDLRHLEENLRQTVKNTVSQVAKKVL  557
PtPAL       DVNSLGLISSRKTAESVDILKLMSTTFLVALCQAIDLRHLEENLRSAVKNTVSHVSKRVL  546
PabPAL2     DVNSLGLISARKTAEAVEILKLMFATYLVGLCQAVDLRHLEENMRSVVKHIVLQAARKTL  562
PsiPAL      DVNSLGLISARKTAEAVEILKLMFATYLVGLCQAVDLRHLEENMRSVVKHIVLQAARKTL  565
PmePAL2     DVNSLGLISARKTAEAIEILKLMFSTYMVGLCQAIDLRHLEENMRSVVKHIVLQAARKTL  600
PsyPAL      DVNSLGLISARKTAEAVEILKLMFATYLVALCQAIDLRHLEENMRSVVKHVVLQAARKTL  450
PsiBAL      DVNSLGLISARKSAEAVDILKLMLSTYLTALCQAVDLRHLEENMLATVKQIVSQVAKKTL  553
PabBAL1     DVNSLGLISARKSAEAVDILKLMLSTYLTALCQAVDLRHLEENMLATVKQIVSQVAKKTL  581
PmeBAL1     DVNSLGLVSARKSAEAIDILKLMLSTYLTALCQAVDLRHLEENMLATVKQIVSQVAKKTL  586
PsyBAL      DVNSLGLVSARKSAEAIDILKLMLSTYLTALCQAVDLRHLEENMLATVKQIVSQVAKKTL  551
PtaBAL1     DVNSLGLVSARKSAEAIDILKLMLSTYLTALCQAVDLRHLEENMLATVKQIVSQVAKKTL  551
PmeBAL2     DVNSLGLVSARKSAEAIDILNLMVSTYLLAICQAADLRHLEENMLSTVKRVVSQVAKKTL  557
PabBAL2     DVNSLGLISARKSAEAIDILNMMVSTFLLALCQAADLRHLEENMLSTVKRVVSQVAKKTL  551
            *:***.*:*:* : ::::**::* ::.: .:*** ***::*: .   *   :  .

PtaBAL3     -----------LQQSIKEQLISVASGIPVYTYLESPCNPSLPLVSALKQTFLDAIVTSH-  580
PmeBAL4     -----------LPESIKVQLVNVARGIPIYTYLESPCDPSLPLLSAIKQTFLDSILTFH-  580
PtaBAL2     YSTTEQGILVLPFGFYENKLLQVVDCLPVFSYMENPTSASSPLTLQLRHVLVEQALKADT  613
PmeBAL3     YSTTDQGILLSPFRFCEKELLQVVDRLPVFSYIEDPTGPSSPLMLQLRQVLVEQALKGNT  605
PabBAL3     YSTTDQGISLLPFRFCENELMQVVDRLPVFSYVEDPAGPSSPLMLQLRHVLVELALKANS  585
PtaPAL4     VVGPRG--ELL--------LLKAVDREPVFSYIDNPFSATSVLTTVLRQVLFEHALEKTT  590
PtaPAL3     VVGSRG--ELLNSRFCEKDLLKVVDLEAVFSYIDNPCSATSVLTTKLRQVLLEHALENKT  605
PtaPAL1     VVGSRG--ELLNSRFCEKDLLRVVDREAVYSYIDNPCSATSVLTTKLRQVLLEHALESKT  605
PtaPAL2     VVGSRG--ELLNSRFCEKDLLKVVDLEAVFSYIDNPCSATSVLTTKLRQVLLEHALENKT  605
PabPAL1     VVGPQG--ELLDSRFCEKDLLKAVEREPVFSYIDNPCSATSVLTTKLRQVLFEHAVEKTT  605
PmePAL1     AVGPQG--ELLPSRFCEKDLLMAVERETVFSYIDNPCSATSVLTTKLRQVLFEHALEKTT  606
OsPTAL      TMNPTG--DLSSARFSEKNLLTAIDREAVFSYADDPCSANYPLMQKLRAVLVEHALTSGD  593
ZmPTAL      TMNPSG--ELSSARFSEKELISAIDREAVFTYAEDAASGSLPLMQKLRAVLVDHALSSGD  593
BdPTAL      TMNPTG--DLSSARFSEKSLLTAIDREAVFSYADDACSANYPLMQKLRAVLVDHALTSSG  593
BoPTAL      TMNPTG--DLSSARFSEKNLLTAIDREAVFTYADDPCSANYPLMQKLRAVLVDHALTSGD  593
BoPAL       STNSTG--DLHVARFCEKDLLKEIDREAVFAYADDPCSPNYPLMKKMRNVLVERALANGM  603
OsPAL       STNSTG--DLHVARFCEKDLLKEIDREAVFAYADDPCSHNYPLMKKLRNVLVERALANGA  603
BdPAL       SMNAMG--GLHIARFCEKDLLTAIDREAVFAYADDPCSPNYPLMQKLRAVLIEHALANGD  607
ZmPAL       SLNARG--GLHNARFCEKDLQTAIDREAVFAYADDPCSPNYALMQKLRAVLVEHALANGD  608
PcPAL       TMGVNG--ELHPSRFCEKDLLRVVDREYIFAYIDDPCSATYPLMQKLRQTLVEHALKNGD  606
AtPAL       TTGVNG--ELHPSRFCEKDLLKVVDREQVYTYADDPCSATYPLIQKLRQVIVDHALINGE  615
PtPAL       TTGANG--ELHPSRFCEKELLKVVDREDVFAYADDPCSATYPLMQKLRQVLVDHALANGE  604
PabPAL2     YTAEDG--SLLDTRFCEKELLQVIDHQPVFSYIDDPTNPSYALMLQLREVLVDESLRLSC  620
PsiPAL      YTAEDG--SLLDTRFCEKELLQVIDHQPVFSYIDDPTNPSYALMLQLREVLVDESLRLSC  623
PmePAL2     YTAEDG--SLLDTRFCEKELLQVIDHQPVFSYIDDPTNPSYALMLQLREVLVDEALKLNC  658
PsyPAL      CTAEDG--SLQDTGFCEKELLQVIDHQPVFSYIDDPTNPSYALMLQLREVLVDEALKSSC  508
PsiBAL      STGLNG--ELLPGRFCEKDLLQIVDNEHVFSYIDDPCNASYPLTQKLRNVLVEHAFKNTD  611
PabBAL1     STGLNG--ELLPGRFCEKDLLQIVDNEHVFSYIDDPCNASYPLTQKLRNVLVEHAFKNTD  639
PmeBAL1     STGLNG--ELLPGRFCEKDLLQIVDNEHVFAYIDDPCNASYPLTQKLRNVLVEHAFKNTE  644
PsyBAL      STGLNG--ELLPGRFCEKDLLQVVDNEHVFSYIDDPCNASYPLTQKLRNILVEHAFKNGE  609
PtaBAL1     STGLNG--ELLPGRFCEKDLLQVVDNEHVFSYIDDPCNASYPLTQKLRNILVERAFKNAE  609
PmeBAL2     STHNGE--LLTAGRFCEKDLLQAVENLHVFAYVDDPCNENYPLMQQLRQVLVAHALNET-  614
PabBAL2     STHNGE--LLTAGRFCEKDLLQAVENMHVFAYIDDPCNENYPLMQQLRQVLVAHALSES-  608
                               *        :::* :.  . .  *   ::  :.   .
```

```
PtaBAL3        -----------------------DIQIVEQITEFECHLKQRLEEEITAIVLSYEERTN-S   616
PmeBAL4        -----------------------DIQIVEKIKEFESHLKQRLEEEITATRLSYEQRTN-I   616
PtaBAL2        ----------------------EQYSLLSKIPIFEEEVRRKLAIEVPLLRQRY-ERGD-F   649
PmeBAL3        ----------------------EQYSLLGKISMFEDELRRILAIEVARIRQRC-ERGD-F   641
PabBAL3        ----------------------DQYSLLGKISMFEEELRRKLAIEVPLIRQKC-ERGD-F   621
PtaPAL4        ---------------------DNDGSILTRVPAFEEELKARIVADVHETRAAC-EKGT-A   627
PtaPAL3        ---------------------DNDASILTRIPAFEKELKAQIVAGVQETRAAC-EKDT-A   642
PtaPAL1        ---------------------DNDASILARIPEFEEELKAQIVAEVQERRAAC-EKGT-A   642
PtaPAL2        ---------------------DNDASILTRIPAFEEELKAQIVAGVQERRAAC-EKGT-A   642
PabPAL1        ---------------------DNDASILTRIPAFEEELKARMVAEVQETREAF-EKRT-A   642
PmePAL1        ---------------------DNDASLLTKIPAFEAELKARIVAEVHEKRDAY-EKGA-A   643
OsPTAL         RRA----------------RGLRVLQDH---QVRGGAPLCAAPGDR-GRPRRR-RQRT-A   631
ZmPTAL         -------------------AEREPSVFSKITRFEEELRAVLPQEVEAARVAV-AEGT-A   631
BdPTAL         VDN----------------AGESEATVFSKINKFEEELRAALPREIEAARVAF-EKGT-A   635
BoPTAL         -------------------AEREPSVFSKITKFEEELRSALPREIEAARVAV-ADGT-A   631
BoPAL          AEF------------------NAETSVFAKVAQFEEELRATLPRAVEAARAAV-ENGT-A   643
OsPAL          AEF------------------NADTSVFAKVAQFEEELRATLPGAIEAARAAV-ENGT-A   643
BdPAL          GER------------------ALETSIFAKVAEFEQNLRAALPKEVEAARASV-ENGT-P   647
ZmPAL          AER------------------DVDTSIFAKVAEFEQQVRAALPKEVEAARAAV-ENGS-P   648
PcPAL          NER------------------NLSTSIFQKIATFEDELKALLPKEVESARAAL-ESGN-P   646
AtPAL          SEK------------------NAVTSIFHKIGAFEEELKAVLPKEVEAARAAY-DNGT-S   655
PtPAL          NEK------------------NTSTSVFQKITAFEEELKALLPKEVESARAAY-DSGN-S   644
PabPAL2        LGKDGESDHNSEAAD---ASGVLSNWVFGRIPLFQQELKARLEDEVPKARERF-DKGD-F   675
PsiPAL         LGKDGESDHNLEAAD---ASGVLSNWVFGRIPLFQQELKARLEDEVPKARERF-DKGD-F   678
PmePAL2        TVKDAESGHKLEAGDGAGAAGTIPNWVFCKIPIFQQELKARLEEEVPKARERF-DRGD-F   716
PsyPAL         PEGNAESDRNLQAAESAGAAGILPNWVFSRIPIFQEELKARLEEEVPKARERF-DNGD-F   566
PsiBAL         GEK------------------DPNTSIFNKITLFEAELKTQLELQVNLARESY-DKGI-S   651
PabBAL1        GEK------------------DPNTSIFNKITLFEAELKTQLELQVNLARESY-DKGI-S   679
PmeBAL1        GEK------------------DPNTSIFNKITLFEAELKTQLESQVNLARDSY-DKGI-S   684
PsyBAL         GEK------------------DPNTSIFNKIPLFEAELKAQLELQVSLARESY-DKGT-S   649
PtaBAL1        GEK------------------DPNTSIFNKIPVFEAELKAQLEPQVSLARESY-DKGT-S   649
PmeBAL2        ---------------------ESQSSIFDKIPVFEKELKEQMEAEIGRARNDYYEKGIAG   653
PabBAL2        ---------------------EIQSSIFNKIPVFEKELKDQMEAEIGRARNDYYEKGIAG   647


PtaBAL3        H-MLEGSCCRTLIHGSKFFPLYAFIREELNAIMMTPRTDHTPQEGT----------QKVF   665
PmeBAL4        HMMLEGSCCRTLIHGSKFFPLYAFIREELNAKLMTPRTDNTPQEDI----------QKVF   666
PtaBAL2        D-------LPNKIRECRTYPLYKFVREELGTSLLSGPRGRTPGEDI----------DKVF   692
PmeBAL3        D-------FPNKIRECRTYPLYDFVREELGTSLLSGPRGRTPGEDI----------DKVF   684
PabBAL3        D-------LPNKVRECRTYPLYEFVREELGTSLLSGPRGRTPGEDI----------DRVF   664
PtaPAL4        L-------VPNRIKDCRSYPLYEFVRAELGTSLLVGTDSRSPGEDF----------DKVF   670
PtaPAL3        L-------VPNRIKDCCSYPLYEFVRVELGTSLLVGTNSRSPGEDFDKVRSSGEDFEKVF   695
PtaPAL1        P-------VPNRIKDCRSYPLYEFVRVELGTSLLVGTNSHSPGEDF----------DKVF   685
PtaPAL2        L-------VPNRIKDCRSYPLYEFVRVELGTSLLVGTNSRSPGEDF----------DKVF   685
PabPAL1        L-------VPNRIKDCRSYPLYEFVRLELGTSLLVGTNSHSPGEDF----------DKVF   685
PmePAL1        F-------VPNRIKDCRSYPLYEFVRLELGTSLLVGTNSHSPGEDF----------DKVF   686
OsPTAL         P-------VANRIVESRSFPLYRFVREELGCVFLTGEKLKSPGEEC----------NKVF   674
ZmPTAL         P-------VANRIADSRSFPLYRFVREELGCVFLTGERLKSPGEEC----------NKVF   674
BdPTAL         P-------IPNLIKDSRSFPLYRFVREELGCVYLTGEKLLSPGEEC----------NKVF   678
BoPTAL         P-------IANRIKESRSFPVYRFVREELGCVYLTGEKLKSPGEEC----------NKVF   674
BoPAL          A-------TPNRITECRSYPLYRFVREELGTAYLTGEKTRSPGEEL----------NKVL   686
OsPAL          A-------IPSRITECRSYPLYRFVREELGTKYLTGEKTRSPGEEL----------NKVL   686
BdPAL          L-------APNRIKDCRSYPLYRFVREELGTEYLTGEKTRSPGEEL----------NKVL   690
ZmPAL          L-------VPNRIKECRSYPLYRFVREEVGTKYLTGEKTRSPGEEL----------NKVL   691
PcPAL          A-------IPNRIEECRSYPLYKFVRKELGTEYLTGEKVTSPGEEF----------EKVF   689
AtPAL          A-------IPNRIKECRSYPLYRFVREELGTELLTGEKVTSPGEEF----------DKVF   698
PtPAL          A-------IENKIKECRSYPLYKFVREELGTGLLTGEKVRSPGEEF----------DKVF   687
PabPAL2        P-------IANRINKCRTYPIYRFVRSELGTDLLTGPKWRSPGEDI----------EKVF   718
PsiPAL         P-------IANRINKCRTYPIYRFVRSELGTDLLTGPKWRSPGEDI----------EKVF   721
PmePAL2        P-------IANRINKCRTYPIYRFVRSELGTDLLTGPKWRSPGEDI----------EKVY   759
PsyPAL         P-------IANRINKCRTYPIYRFVRSELGTDLLTGPKWRSPGEDI----------EKVF   609
PsiBAL         P-------LPNRIQECRSYPLYEFVRTQLGTKLLSGTRTTSPGEVI----------ELVY   694
PabBAL1        P-------LPNRIQECRSYPLYEFVRTQLGTKLLSGTRTISPGEVI----------ELVY   722
PmeBAL1        P-------LPNRIQECRSYHLYEFVRNQLGTKLLSGARTTSPGEVI----------ELVY   727
PsyBAL         P-------LPNRIQECRSYPLYEFVRKQLGTKLLSGTRTISPGEVI----------EVVY   692
PtaBAL1        P-------LPNRIQECRSYPLYEFVRNQLGTKLLSGTRTISPGEVI----------EVVY   692
PmeBAL2        S-------IPNRIQDCRSFPLYDFARSQLGTQLLSGDRTTSPGEYI----------GKVY   696
PabBAL2        S-------VSNRIQECRSFPLYDFVRSQLGTQLLSGDRVTSPGEYI----------EKVY   690
                         . :  .  : :* * * ::.   :      :* *              *
```

```
PtaBAL3       DAIVDGRITAPLLQCLNGFMN---------------------------------------  686
PmeBAL4       DAIVDGRITVPLLLCLNGFMK---------------------------------------  687
PtaBAL2       IAITEGKLEGRLMECLDGWNESPGPFNDLKKNANHYDILKSHNNNSCVWSWFQQIGGPQV  752
PmeBAL3       IAITEGKLEGRLMECLDGWNESPGPFNDLKKNANNDDVVRTHSKNSCVWSWFQHIGGPPV  744
PabBAL3       IAITEGKLEGRLMECLDGWNESPGPFNGMKKNANNYDIVKTPNNNSCVWSWFQKMGGPQV  724
PtaPAL4       VAINEGKAVAPLFKCLEGWNGAPIPI----------------------------------  696
PtaPAL3       VAINEGKAVEPLFKCLEEWNGAPIPILNLNITN------RNPKESECCRS----------  739
PtaPAL1       VAINEGKTVEPLFKCLEKWNGAPIPI----------------------------------  711
PtaPAL2       VAINEGKAVEPLFKCLEKWNGVPIPI----------------------------------  711
PabPAL1       VAINEGKAVEPLFKCLERWNGAPIPI----------------------------------  711
PmePAL1       VAINEGKAVEPLLKCLERWNGAPIPI----------------------------------  712
OsPTAL        LGISQGKLIDPMLDCLKEWNGEPLPIN---------------------------------  701
ZmPTAL        VGISQGKLVDPMLECLKEWDGKPLPINVK-------------------------------  703
BdPTAL        IGISQGKLIDPMLECLKEWNGEPLPINVV-------------------------------  707
BoPTAL        IGISQGKLIDPMLECLKEWNGEPLPIN---------------------------------  701
BoPAL         LAINQGKHIDPLLECLKEWNGEPLPIN---------------------------------  713
OsPAL         VAINEGKHIDPLLECLKEWNGEPLPIC---------------------------------  713
BdPAL         VAMNQRKHIDPLLECLKEWNGEPLPLC---------------------------------  717
ZmPAL         VAINQRKHIDPLLECLKEWNGEPLPLC---------------------------------  718
PcPAL         IAMSKGEIIDPLLECLESWNGAPLPIC---------------------------------  716
AtPAL         TAICEGKIIDPMMECLNEWNGAPIPIC---------------------------------  725
PtPAL         TAMCQGKIIDPMLECLGEWNGAPLPIC---------------------------------  714
PabPAL2       EGICEGKMGEVILKCLDAWRGCAGPFTPRAYPA------SPAAFNTSYWAWFDNTKSPSA  772
PsiPAL        EGICEGKMGEVILKCLDAWRGCAGPFTPRAYPA------SPAAFNTSYWAWFDNTKSPSA  775
PmePAL2       EGICDGKIGDVILKCLDAWRGCAGPFTPRAYPA------SPAAFNTSYWAWFDSTKSPSA  813
PsyPAL        EGICQGKIGDVILKCLDAWGGCAGPFTPRAYPA------SPAAFNASYWAWFDSTKSPSA  663
PsiBAL        DAISEDKIIGPLLKCVEGWKATPGPF----------------------------------  720
PabBAL1       DAISEDKIIGPLLKCVEGWKATPGPF----------------------------------  748
PmeBAL1       DAISEDKIIGPLFKCLDGWKATPGPF----------------------------------  753
PsyBAL        DAISEDKVIVPLFKCLDGWKGTPGPF----------------------------------  718
PtaBAL1       DAISEDKVIVPLFKCLDGWKGTPGPF----------------------------------  718
PmeBAL2       DGIREGKIIAPLLKCLDGWSGTPGPFPS--------------------------------  724
PabBAL2       VGICEGKIISPLFKCLDGWSGTPGPFQS--------------------------------  718
               .: . .    :: *:  :

PtaBAL3       ---------------     686
PmeBAL4       ---------------     687
PtaBAL2       NGGKGYWLLTIA---     764
PmeBAL3       NGIKGYWLLSIA---     756
PabBAL3       NGGKGYWLLSIA---     736
PtaPAL4       ---------------     696
PtaPAL3       ---------------     739
PtaPAL1       ---------------     711
PtaPAL2       ---------------     711
PabPAL1       ---------------     711
PmePAL1       ---------------     712
OsPTAL        ---------------     701
ZmPTAL        ---------------     703
BdPTAL        ---------------     707
BoPTAL        ---------------     701
BoPAL         ---------------     713
OsPAL         ---------------     713
BdPAL         ---------------     717
ZmPAL         ---------------     718
PcPAL         ---------------     716
AtPAL         ---------------     725
PtPAL         ---------------     714
PabPAL2       TSGRGFWSAQQQQVL     787
PsiPAL        TSGRGFWSAQQQQVL     790
PmePAL2       TSGRGFWSAQQQQIL     828
PsyPAL        TSGRGFWSAQQQQVL     678
PsiBAL        ---------------     720
PabBAL1       ---------------     748
PmeBAL1       ---------------     753
PsyBAL        ---------------     718
PtaBAL1       ---------------     718
PmeBAL2       ---------------     724
PabBAL2       ---------------     718
```

Figure S18 Latitudinal variation in the SNP (Ala228Ala) from PabPAL2 gene in Norway spruce populations across Sweden. (a) Cline in the allele frequencies of Ala228Ala. (b) Cline in the genotype frequencies of Ala228Ala. One-way ANOVA and Tukey's posthoc test was performed with the genotype frequencies. Tukey's posthoc categorization is indicated above the bars.

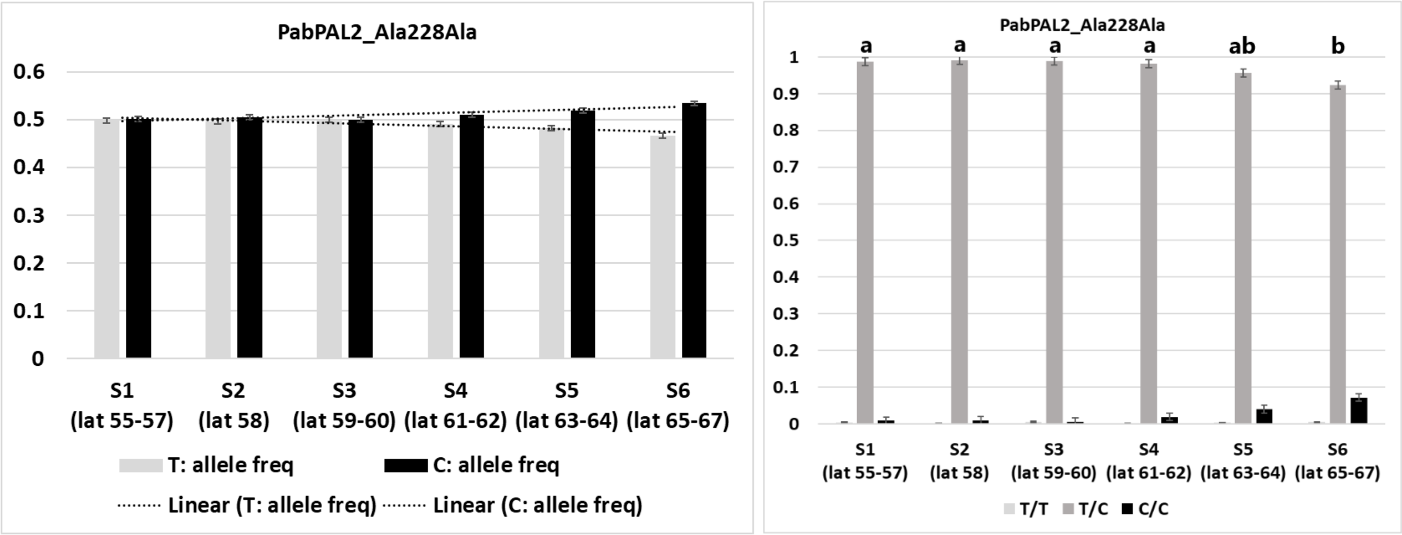

(a) (b)

Figure S19 Latitudinal variation in the SNP (Pro356Pro) from PabPAL2 gene in Norway spruce populations across Sweden. (a) Cline in the allele frequencies of Pro356Pro. (b) Cline in the genotype frequencies of Pro356Pro. One-way ANOVA and Tukey's posthoc test was performed with the genotype frequencies. Tukey's posthoc categorization is indicated above the bars.

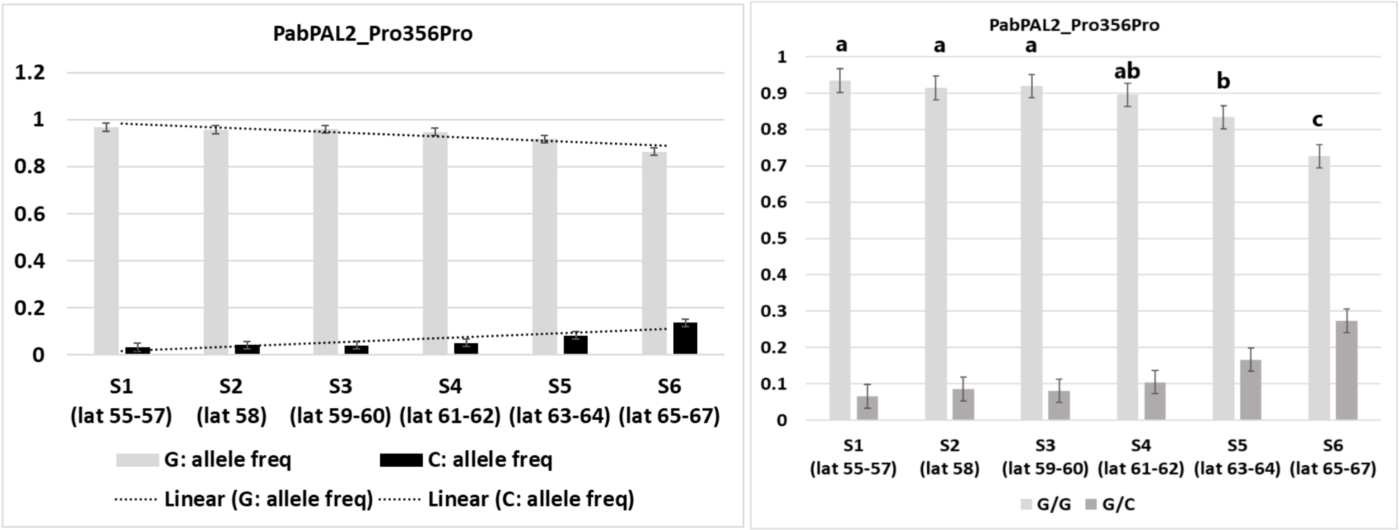

(a) (b)

Figure S20 Latitudinal variation in the SNP (Ala383Thr) from PabPAL2 gene in Norway spruce populations across Sweden. (a) Cline in the allele frequencies of Ala383Thr. (b) Cline in the genotype frequencies of Ala383Thr. One-way ANOVA and Tukey's posthoc test was performed with the genotype frequencies. Tukey's posthoc categorization is indicated above the bars.

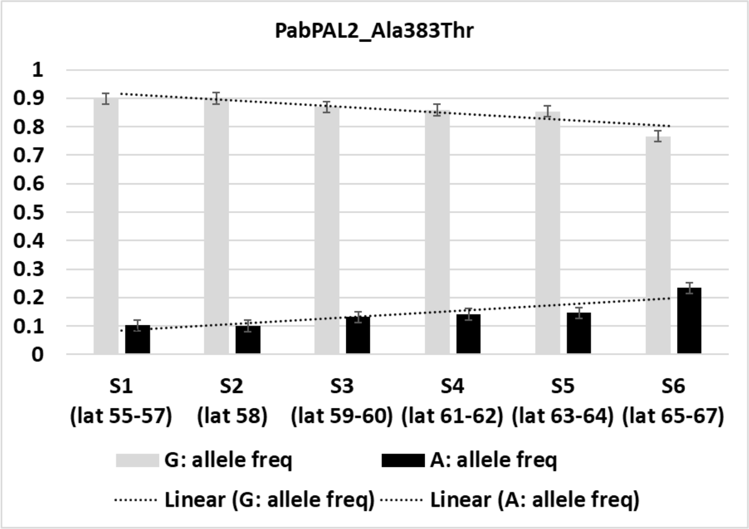

(a)

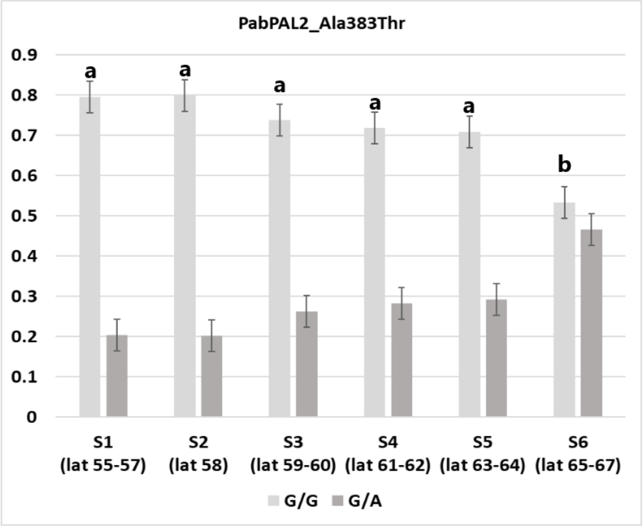

(b)

Figure S21 Latitudinal variation in the SNP (Ala535Val) from PabBAL1 gene in Norway spruce populations across Sweden. (a) Cline in the allele frequencies of Ala535Val. (b) Cline in the genotype frequencies of Ala535Val. One-way ANOVA and Tukey's posthoc test was performed with the genotype frequencies. Tukey's posthoc categorization is indicated above the bars.

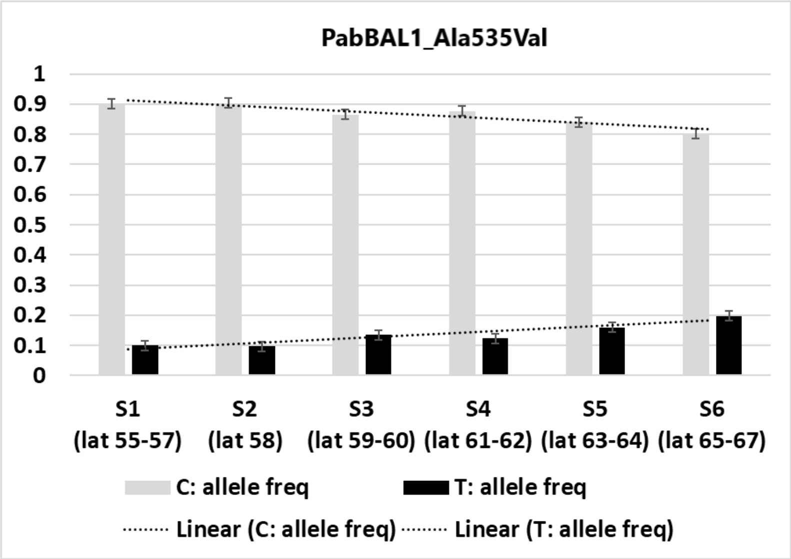

(a)

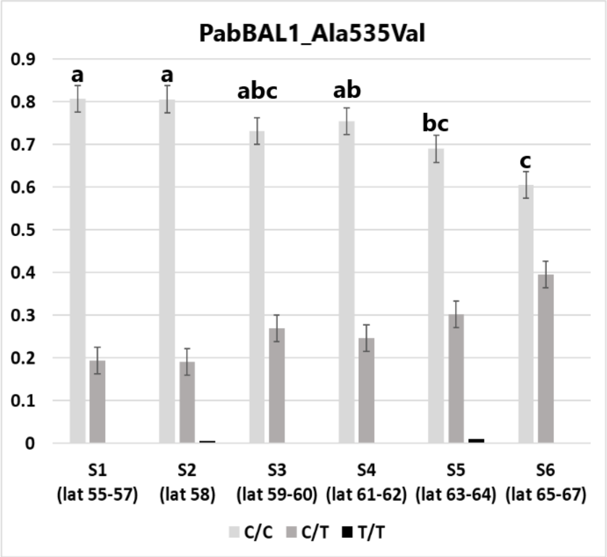

(b)

Figure S22 Latitudinal variation in the SNP (Ser595Phe) from PabBAL3 gene in Norway spruce populations across Sweden. (a) Cline in the allele frequencies of Ser595Phe. (b) Cline in the genotype frequencies of Ser595Phe. One-way ANOVA and Tukey's posthoc test was performed with the genotype frequencies. Tukey's posthoc categorization is indicated above the bars.

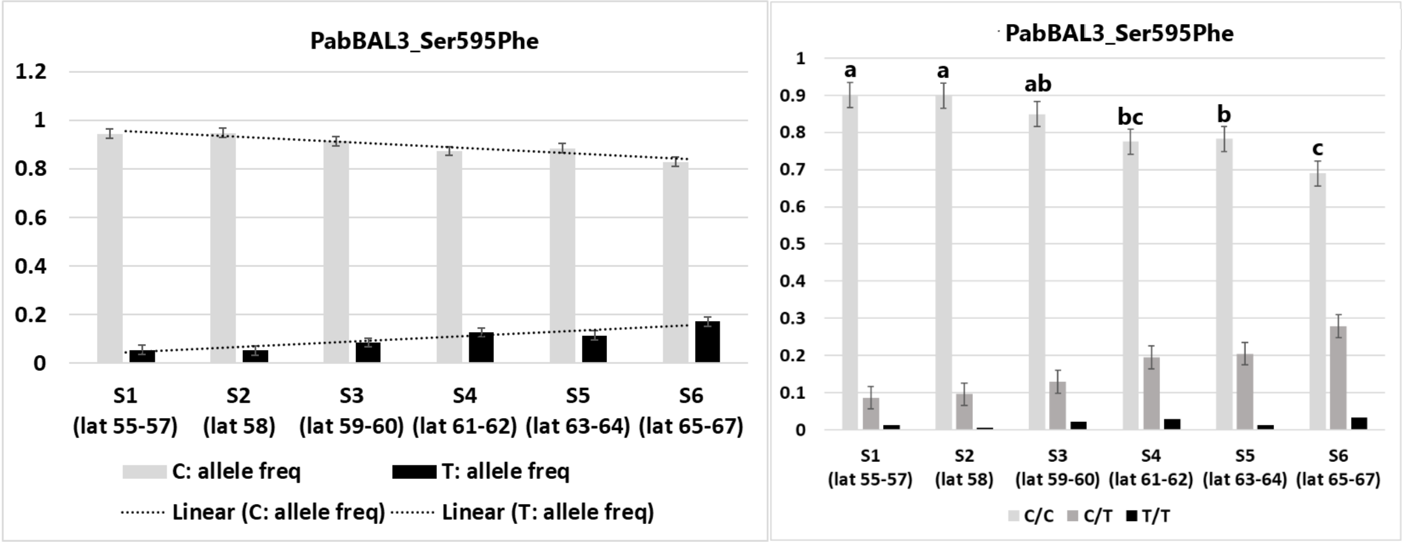

(a) (b)

Figure S23 Latitudinal variation in the SNP (Leu601Phe) from PabBAL3 gene in Norway spruce populations across Sweden. (a) Cline in the allele frequencies of Leu601Phe. (b) Cline in the genotype frequencies of Leu601Phe. One-way ANOVA and Tukey's posthoc test was performed with the genotype frequencies. Tukey's posthoc categorization is indicated above the bars.

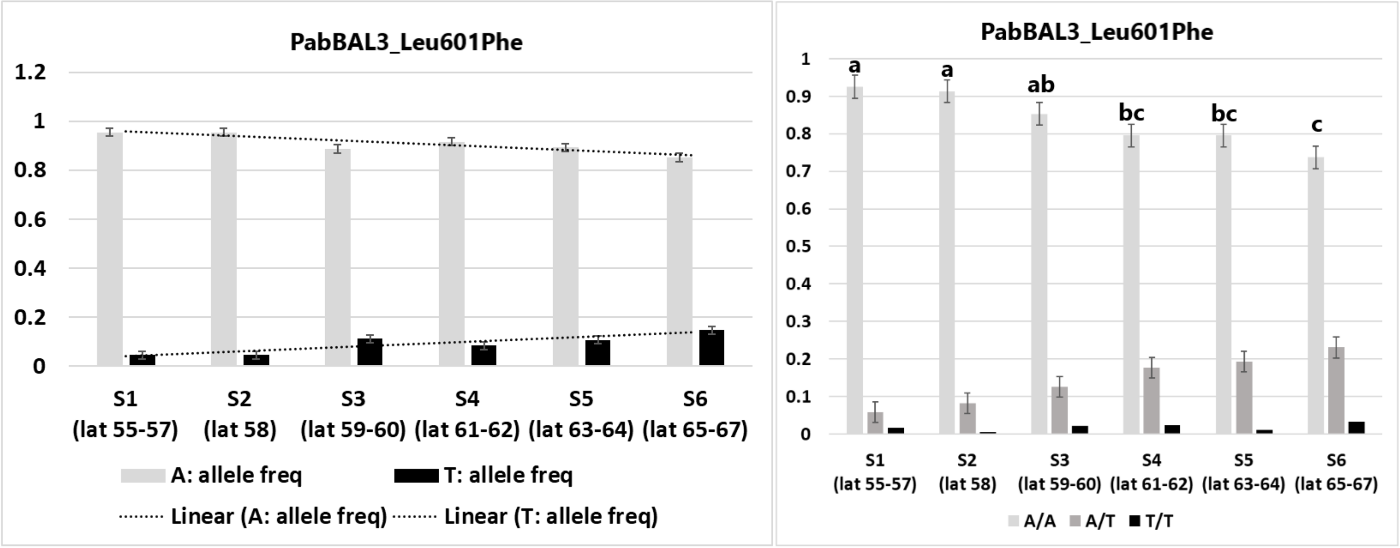

(a) (b)
